# Synthesis and fluorescent properties of boroisoquinolines, a new family of fluorophores†

**DOI:** 10.1039/c8ra08241c

**Published:** 2018-11-15

**Authors:** Dénes Sóvári, Attila Kormos, Orsolya Demeter, András Dancsó, György Miklós Keserű, Mátyás Milen, Péter Ábrányi-Balogh

**Affiliations:** Hungarian Academy of Sciences, Research Centre for Natural Sciences, Institute of Organic Chemistry, Medicinal Chemistry Research Group 1519 Budapest POB 286 Hungary abranyi-balogh.peter@ttk.mta.hu +36 1 3826961; Hungarian Academy of Sciences, Research Centre for Natural Sciences, Institute of Organic Chemistry, Chemical Biology Research Group 1519 Budapest POB 286 Hungary; Egis Pharmaceuticals Plc., Directorate of Drug Substance Development 1475 Budapest POB 100 Hungary

## Abstract

First representatives of a new family of isoquinolines, so called boroisoquinolines, were synthesized and characterized. The synthesis was based on the insertion of the difluoroboranyl group into the 1-methylidene-3,4-dihydroisoquinoline core. The optimization of the 2-difluoroboranyl-3,4-dihydroisoquinoline-1(2*H*)-ylidene core led to efficient fluorescence in a range of 400–600 nm with outstanding (>100 nm) Stokes shifts. The compounds might be suitable for reversible or irreversible labelling of proteins, particularly the cannabinoid receptor CB_2_.

## Abstract 2

First representatives of a new family of isoquinolines, so called boroisoquinolines, were synthesized and characterized.

## Graphical abstract

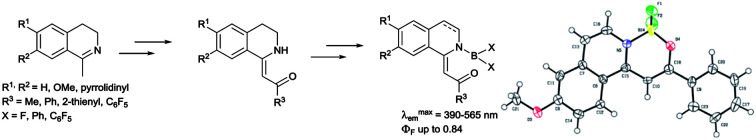

## Introduction

**Fig. 1:**
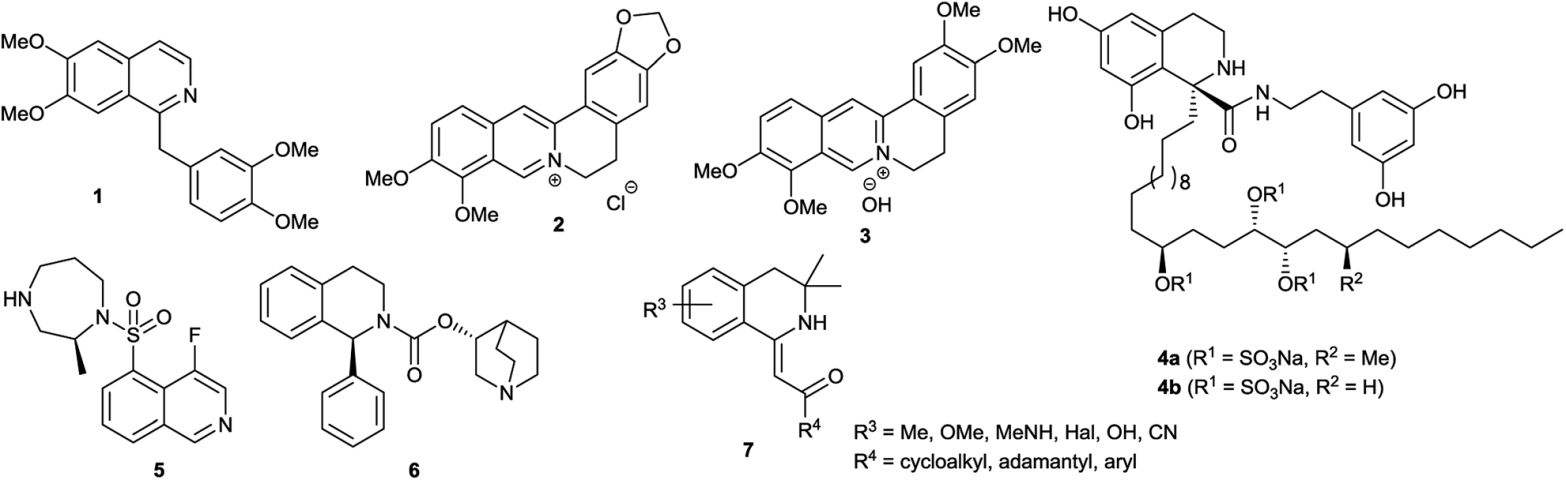
Structures of pharmaceutically active isoquinoline derivatives.

**Fig. 2:**
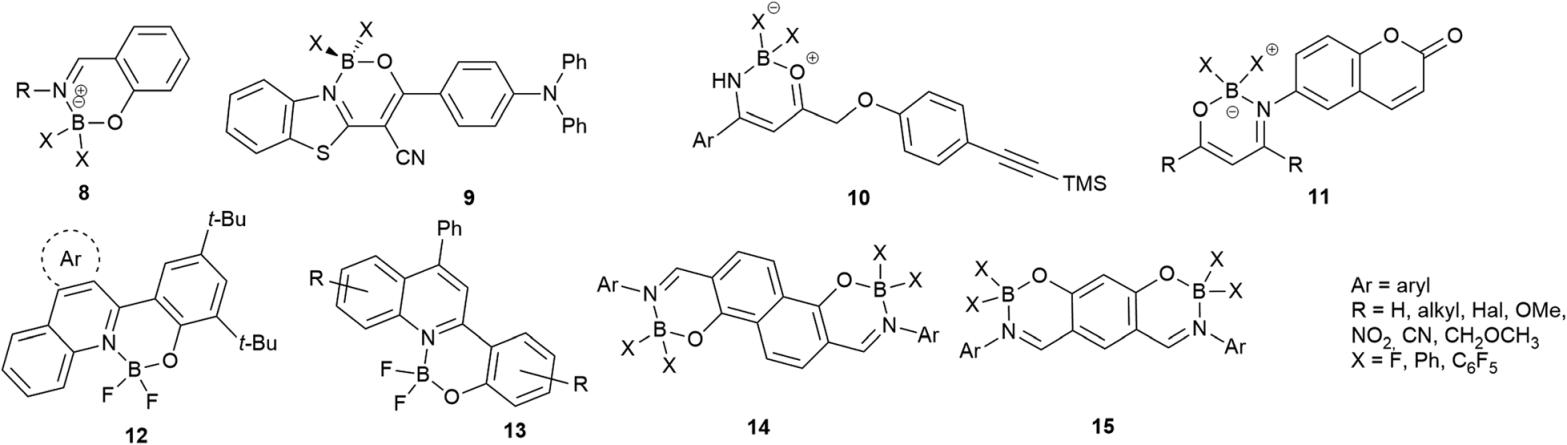
Boranyls (8) and related compounds containing nitrogen–boron–oxygen structural element (NBO-complexes, 9–15).

The isoquinoline core is represented in a large number of biologically active compounds containing alkaloids and also synthetic heterocycles (Fig. 1). The alkaloids can be found widely in various plants^1–4^ and marine invertebrates.^5^ A well-known pharmacologically significant compound is papaverine (1) that was isolated from opium poppies (*Papaver somniferum*) and was used as a smooth muscle relaxant.^6^ Other biologically active representatives are the members of the berberine (2) family that were isolated from various herbs.^7,8^ These plants (*e.g. Coptis chinensis*) have been used in traditional Chinese medicine for the treatment of infections, skin problems and diabetes.^9–12^ Among the latter compounds palmatine (3) should be mentioned as a widely used tetracyclic isoquinoline against, among others, hypertension and inflammation.^13^ From a marine sponge *Jaspis* sp. also, more complex isoquinoline natural products were isolated such as jasisoquinoline A and B (4a and 4b, respectively) that possess anticancer activity.^14^ Ripasudil (5, Glanatec®) activates the Rho-kinase, hence it is used in the treatment of glaucoma and increased eye-pressure.^15,16^ Furthermore, many isolated or synthesized isoquinoline derivatives exhibit a vast spectrum of biological activities including anti-HIV,^17^ antitubercular,^18^ antiplasmodial,^19^ anti-Alzheimer's^20^ and antifungal effects^21^ and some representatives are non-competitive AMPA [2-amino-3-(3-hydroxy-5-methylisoxazol-4-yl)propionic acid] receptor antagonists.^22^ In addition, the commercially available drug solifenacin (Vesicare®, 6), a 1-aryl-tetrahydroisoquinoline derivative shows competitive cholinergic receptor antagonist effect used in the treatment of contraction of overactive bladder.^23,24^ Members of the 2′-(3,3-dimethyl-3,4-dihydroisoquinoline-1-ylidene)-1-ethane-1-one family (7) should be mentioned as non-toxic and specific nanomolar CB_2_ agonist, while other derivatives of this family can inhibit phosphodiesterase 7 (PDE7). On this basis, these compounds might be considered as preventive or treating agents for asthma, autoimmune diseases, rheumatoid arthritis, Crohn's disease or carcinomatous pain.^25–28^ Besides the pharmaceutical applications, isoquinoline derivatives were also utilized as chiral ligands for catalytic asymmetric reactions^29^ or as electrophosphorescent iridium complexes.^30^ Boranyls (8) and related compounds containing nitrogen–boron–oxygen structural element (NBO-complexes) are a recently developed family of fluorescent molecules (Fig. 2). The photophysical properties of several known NBO-complexes are summarized in Table 1. These compounds were designed to overcome the drawbacks of BODIPY's *e.g.* small Stokes-shift, difficult industrial synthesis.^31^ The synthesis of boranyls is usually starting from an imino compound containing a hydroxyl or an enolizable oxo group at the γ-position. The parent compounds are reacted with a trisubstituted borane, mostly BF_3_ or B(aryl)_3_ resulting in a disubstituted borane group inserted between the nitrogen and the oxygen atom. The photophysical characterization of these compounds showed acceptable Stokes-shifts with good *ε* and *Φ* values (Table 1, entry 1).^32^ It was observed that in the case of borobenzothiazoles^33^ (9) a push–pull system between a disubstituted amino and a nitrile group was required for an outstanding Stokes-shift (149–170 nm, entry 2), but the quantum yield of the compounds was weak. Minuti and co-workers designed boranyls for biological application by the ring opening of isoxazoles to 10 equipped with an acetylene tail in order to the click labeling of azidoproteins.^34^ Only one member of the boranyls derived from coumarins (11) showed weak fluorescence but on the contrary, the phosphorescence of those was remarkable (Table 1, entry 3).^35^ Boroquinolines (12, 13) were synthesized from salicylic aldehyde, aniline and phenylacetylene, showing a Stokes-shift of 80–200 nm and green fluorescence (Table 1, entries 4, 5).^36,37^ From naphthalene dialdehyde multiboranyls (14) were synthesized, but the larger molecule size caused lower fluorescence.^38^ Nevertheless, a nice red shift was obtained with a wide range of quantum yield (Table 1, entry 6). Related compounds, so called polyboranyls (15) were designed from *m*-phthalic aldehyde exhibiting large Stokes-shift with medium molar absorptivity and quantum yield (Table 1, entry 7).^39^

**Table 1:** Photophysical properties of NBO-complexes

Entry		Solvent	*λ* ^max^_absz_ [nm]	*λ* ^max^_em_ [nm]	Stokes-shift [nm]	*ε* [M^−1^ cm^−1^]	*Φ*_F_ [−]
1	8^32^	CH_2_Cl_2_	344–387	380–432	14–68	13100–67200	0.22–0.73
2	9^33^	MeCN	442–450	599–612	149–170	—	0.004–0.008
3	11^35^^a^	THF/dioxane	331	420	89	33660	—
4	12^36^	CHCl_3_	385–417	523–543	126–145	8317–11482	0.18–0.32
5	13^37^	CHCl_3_	288–385	467–538	82–201	14300–38500	0–0.22
6	14^38^	CH_2_Cl_2_	495–589	457–609	37–183	17300–148700	0–0.56
7	15^39^	CH_2_Cl_2_	495–589	533–683	38–101	13100–44500	0–0.81

a Only one member showed fluorescence.

As a continuation of our efforts on the field of developing biologically interesting isoquinoline derivatives,^40^ the objective of our work was the synthesis of new fluorescent members of the 2′-(3,4-dihydroisoquinoline-1-ylidene)-1-ethan-1-one (7) family equipped with BR_2_ groups.

## Results and discussion

**Scheme 1:**
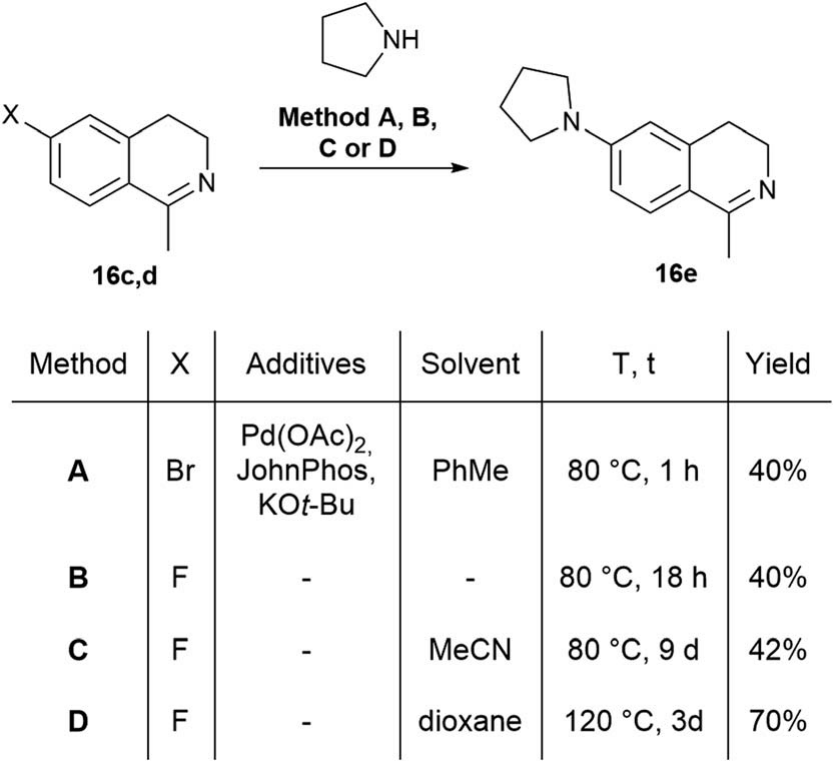
The synthesis of 1-methyl-7-pyrrolidine-1-yl-3,4-dihydroisoquinoline (16e).

The fluorescent molecules were envisaged to be obtained by the acylation of dihydroisoquinolines followed by the incorporation of a boranyl group. Initially, the starting 7-methoxy (16a), 6,7-dimethoxy (16b), 7-bromo (16c) and 7-fluoro-3,4-dihydroisoquinoline (16d) have been synthesized by slight modification of literature procedures.^41,42^ In most cases, the corresponding phenylacetonitriles were transformed to phenylethylamines using borane in THF,^43^ followed by the acylation with acetyl chloride. The ring closure was achieved with a modified Bischler–Napieralski reaction after Larsen using oxalyl chloride in the presence of FeCl_3_ and the subsequent reaction with sulphuric acid in MeOH.^44^ In the case of 7-pyrrolidinyl-1-methyl-3,4-dihydroisoquinoline (16e), the pyrrolidine group was inserted either with Buchwald–Hartwig reaction of 16c or with the aromatic nucleophilic substitution of 16d (Scheme 1).

The Buchwald–Hartwig coupling was performed in the presence of Pd(OAc)_2_, JohnPhos and KO*t-*Bu in toluene leading to 16e in a yield of 40% (Scheme 1, Method A).^45^ In order to improve the outcome, several substitution reactions were tried using 16d (Method B, C and D).^46,47^ The best result was obtained reacting 16d with 6 equiv. of pyrrolidine in dioxane at 120 °C in a sealed tube for 3 days resulting in 16e in a yield of 70% (Method D). Notably, for the purification of 16e steam distillation and normal phase flash chromatography was required.

**Scheme 2:**
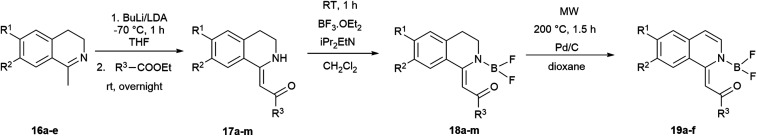
Synthesis of 1-methylidene-1,2,3,4-tetrahydroisoquinolines (17) and boroisoquinolines 18 and 19.

The synthesis of the 1-methylidene-1,2,3,4-tetrahydroisoquinolines (17) was accomplished by the slight modification of the method of Dannhardt *et al.* using BuLi or lithium-diisopropylamide (LDA) to deprotonate the methyl group of the 1-methyl-3,4-dihydroisoquinolines (16a–e) derivatives followed by the addition of the appropriate ester (Scheme 2).^48^ Heterocycles 17a–h were isolated in moderate yields (12–65%, Table 2, entries 1–8). In the case of 4-nitrobenzoic acid ethyl ester, the products (17i,j) could be isolated in traces with BuLi (3–5%), but using LDA the yield of 17j was raised to 54% (Table 2, entries 9, 10). Changing the methoxy group on the isoquinoline to pyrrolidine also lowered the yields (16–27%) possibly due to the interaction of the pyrrolidine nitrogen with the BuLi. The use of a larger amount of BuLi, or changing the base to *sec*-BuLi or BuLi–TMEDA did not give better results, but again, the application of LDA led to a significant raise in the isolated yields (20–72%, Table 2, entries 11–13).

**Table 2:** Results for the synthesis of 1-methylidene-1,2,3,4-tetrahydroisoquinolines (17) and boroisoquinolines 18, 21

#		R^1^	R^2^	R^3^	17^a^ [%]	18^a^ [%]	19^a^ [%]
1	a	MeO	H	Me	46	77	73
2	b	MeO	MeO	Me	50	78	70
3	c	MeO	H	Ph	45	70	77
4	d	MeO	MeO	Ph	53	88	89
5	e	MeO	H	2-Thienyl	58	50	52
6	f	MeO	MeO	2-Thienyl	48	85	48
7	g	MeO	H	C_6_F_5_	12	85	—
8	h	MeO	MeO	C_6_F_5_	57	89	—
9	i	MeO	H	4-O_2_N–C_6_H_4_	5	—	—
10	j	MeO	MeO	4-O_2_N–C_6_H_4_	54 (3) b	49	—
11	k	Py^c^	H	Me	41 (26) b	78	—
12	l	Py^c^	H	Ph	72 (27) b	97	—
13	m	Py^c^	H	C_6_F_5_	20 (16) b	81	—

a Isolated yields.

b Yields with LDA, results with BuLi are in parentheses.

c Pyrrolidine-1-yl.

The difluoro-boroisoquinoline structure (18) was constructed by reacting compounds 17 with boron trifluoride diethyl etherate in the presence of 5 equiv. of diisopropylethylamine (DIPEA) in dichloromethane (Scheme 2).^32^ In order to synthesize fully aromatic derivatives with respect to its expected advantageous effect on the fluorescence, compounds 18a–f were dehydrogenated with Pd/C in dioxane at 200 °C for 90 min (Scheme 2). Boroisoquinolines 19a–f were isolated in good yields (48–89%, Table 2, entries 1–6). In the case of 18k–m, the pyrrolidine ring was aromatized during the reaction, a selective oxidation could not be developed. Dehydrogenating derivatives with C_6_F_5_ substituent produced only degradation products that could be detected by HPLC-MS with loss of various fluorine atoms. In the case of 18j, the nitro group was converted to amino function, and decomposition products were observed.

**Scheme 3:**
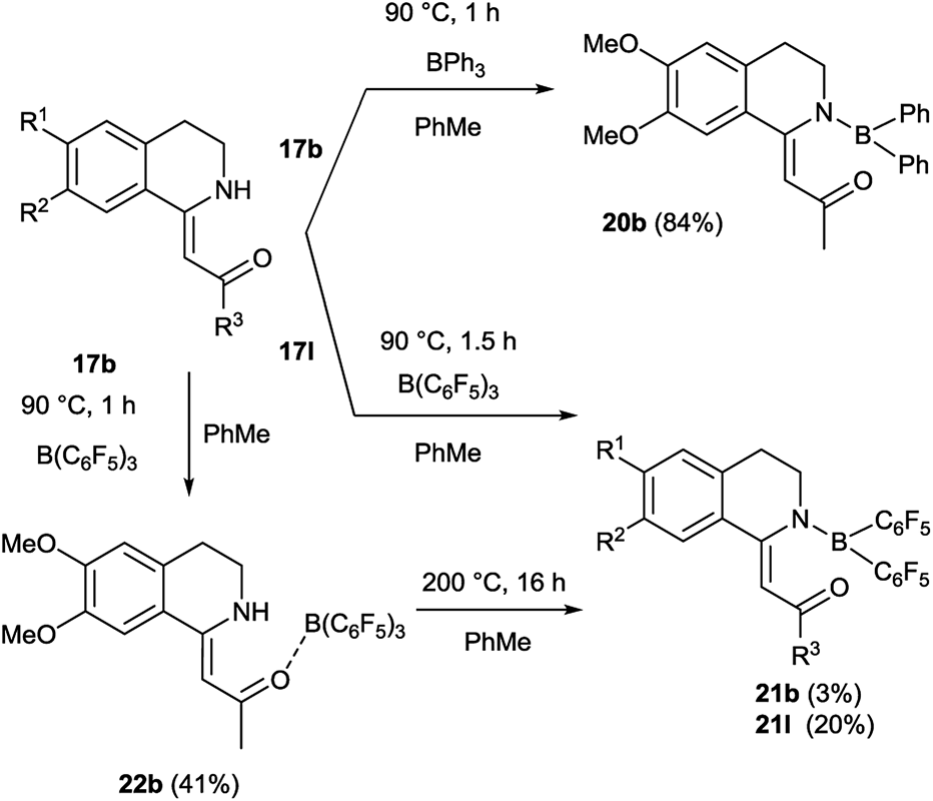
Synthesis of boroisoquinolines 20, 21, 22.

We aimed to synthesize further boranyl derivatives using triaryl boranes. A diphenyl-boroisoquinoline (20b) was prepared in a yield of 84% by refluxing 17b with triphenylborane in toluene at 90 °C (Scheme 3).^31^ In the case of B(C_6_F_5_)_3_ as the borylating agent, the formation of the desired bis(perfluorophenyl)-boroisoquinoline was observed depending on the starting compound either in a one-step reaction in toluene at 90 °C leading to 21l in a yield of 20%, or in a two-step procedure through the heating of tris(perfluorophenyl)-borosisoquinoline intermediate 22b at 200 °C resulting in 21b in a poor yield (3%) (Scheme 3).

**Fig. 3:**
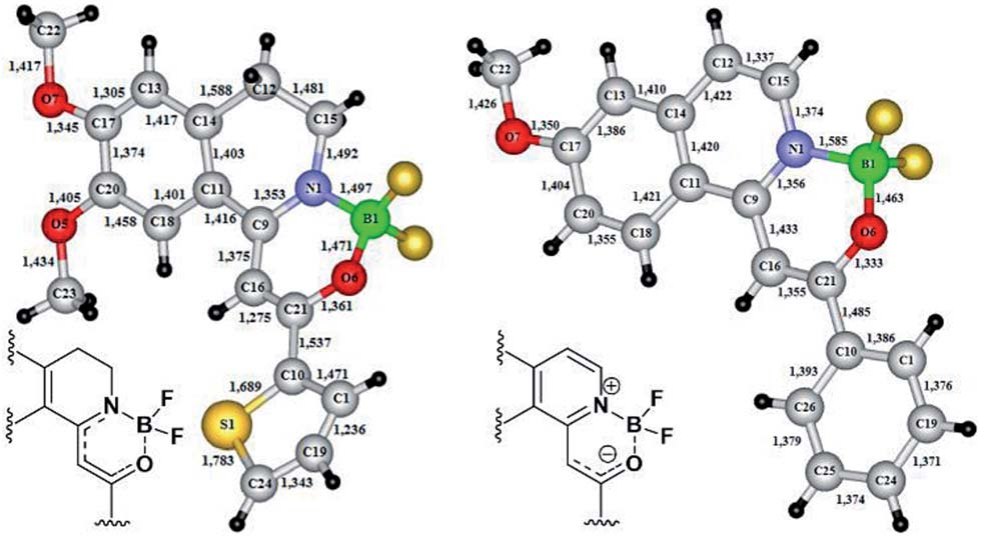
X-ray structures of boroisoquinolines 18f and 19c.

In the cases of 18f and 19c the exact structure of the new heterocyclic family members was elucidated by single crystal X-ray diffraction measurements (Fig. 3). From the crystal structures it could be concluded, that until the nitrogen atom of 18f has pyramidal conformation, in 19c the nitrogen is planar. The bond lengths proved the boron–nitrogen bonding and the boron–oxygen coordination. The proposed electron-distribution and bonding in the NBO containing ring is shown on the figures next to the crystal structures. Representative bond lengths and bond angles are shown in Table 3. The detailed XRD data and ORTEP figures can be found in the ESI.†

**Table 3:** Representative bond lengths and bond angles

18f		19c	
Bond length (Å)	Bond angle (°)	Bond length (Å)	Bond angle (°)
B1–N1 = 1.497	C21–O6–B1 = 119.0		C21–O6–B1 = 119.0
B1–O6 = 1.471	C9–N1–C15 = 125.4		C9–N1–C15 = 125.4
N1–C9 = 1.353	C15–N1–B1 = 114.6	B1–N1 = 1.585	C15–N1–B1 = 114.6
C9–C16 = 1.375	N1–C9–C11 = 112.8	B1–O6 = 1.463	N1–C9–C11 = 112.8
C16–C21 = 1.275	O6–C21–C10 = 107.8	N1–C9 = 1.365	O6–C21–C10 = 107.8
O6–C21 = 1.361	C10–C21–C16 = 128.6	N1–C15 = 1.374	C10–C21–C16 = 128.6
N1–C15 = 1.492	F3–B1–F4 = 107.5	C15–C12 = 1.337	F3–B1–F4 = 107.5
	F3–B1–N1 = 108.6	C9–C16 = 1.433	F3–B1–N1 = 108.6
	C9–N1–B1 = 119.5	C16–C21 = 1.355	C9–N1–B1 = 119.5
	N1–C9–C16 = 120.6		N1–C9–C16 = 120.6
	N1–C15–C12 = 110.8		N1–C15–C12 = 110.8
	C9–C16–C21 = 121.1		C9–C16–C21 = 121.1
	O6–C21–C16 = 123.5		O6–C21–C16 = 123.5
	F3–B1–O6 = 105.8		F3–B1–O6 = 105.8
	F4–B1–O6 = 109.8		F4–B1–O6 = 109.8
	F4–B1–N1 = 114.4		F4–B1–N1 = 114.4
	O6–B1–N1 = 110.3		O6–B1–N1 = 110.3

The synthesis was followed by the extensive photophysical analysis of the new boroisoquinolines. The absorbance and fluorescence spectra were measured for compounds 18a–h,k–m, 19a–f, 20b, 21b,l and 22b (Table 4) in acetonitrile. It should be noted, that the nitro group of 18j was disadvantageous for the fluorescence, as the intensity was measured in the range of the noise.

**Table 4:** Photophysical properties of the boroisoquinolines

Entry		R^1^^a^	R^2^	R^3^	BX	*λ* ^max^_abs_ [nm]	*λ* ^max^_exc_ [nm]	*λ* ^max^_em_ [nm]	Δ*λ* [nm]
1	18a	MeO	H	Me	BF_2_	328		390	62
2	18b	MeO	MeO	Me	BF2	321	339	431	110
3	18k	Py	H	Me	BF_2_	383	379	450	67
4	18c	MeO	H	Ph	BF_2_	357		411	54
5	18d	MeO	MeO	Ph	BF_2_	361	380	483	122
6	18l	Py	H	Ph	BF_2_	408	406	537	129
7	21l	Py	H	Ph	B(C_6_F_5_)_2_	417	415	544	127
8	18e	MeO	H	2-Thienyl	BF_2_	374		437	63
9	18f	MeO	MeO	2-Thienyl	BF_2_	383		448	65
10	18g	MeO	H	C_6_F_5_	BF_2_	334	343	435	101
11	18h	MeO	MeO	C_6_F_5_	BF_2_	334	356	537	203
12	18m	Py	H	C_6_F_5_	BF_2_	411	410	565	154
13	20b	MeO	MeO	Me	BPh_2_	337	387	453	116
14	21b	MeO	MeO	Me	B(C_6_F_5_)_2_	346	372	440	94
15	22b	MeO	MeO	Me	B(C_6_F_5_)_3_	357	368	465	108
16	19a	MeO	H	Me	BF_2_	355		410	55
17	19b	MeO	MeO	Me	BF_2_	371		415	45
18	19c	MeO	H	Ph	BF_2_	378	372	487	109
19	19d	MeO	MeO	Ph	BF_2_	396		458	62
20	19e	MeO	H	2-Thienyl	BF_2_	401		471	70
21	19f	MeO	MeO	2-Thienyl	BF_2_	410		480	70

a Py = pyrrolidine-1-yl.

Regarding the different boranyls, although *λ*^max^-s of the di- or triarylboranyl derivatives (20b, 21b,l, 22b, Table 4, entries 7, 13–15) were uniformly higher than those of the difluoroboranyls, the Stokes-shift did not raise or even decreased. This observation has led to the conclusion that the fluoro derivatives should be developed further and this was supported by sterical factors too, namely it is presumed, that the extensive diarylboranyl group would be less favoured in a binding pocket of a protein than the smaller difluoroboranyl group.

**Fig. 4:**
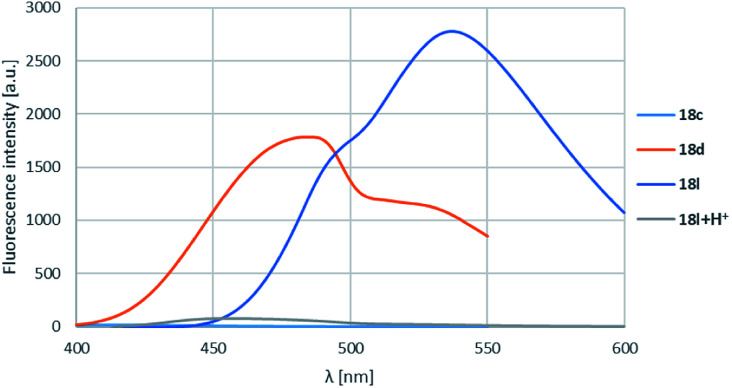
R^1^, R^2^ substituent effects for boroisoquinoline 18.

On the isoquinoline ring electron donating substituents were chosen for the substitution envisioning that the electron donation will raise the fluorescence. This assumption was confirmed as the 6-methoxy derivatives showed the weakest fluorescence intensity and the lowest *λ*^max^-s, while the insertion of a second methoxy group or changing the methoxy to pyrrolidinyl initiated significant increase in intensity and also *λ*^max^-s (Table 4, entries 1–3, 4–6 and 10–12; representative spectra for 18c,d,l shown on Fig. 4) leading the fluorescence into the >500 nm range. In order to prove the electron donating effect of the pyrrolidine substituent, the fluorescence spectrum of 18l was measured in the presence of HCl (1 equiv.) (Fig. 4, grey spectrum) and a significant decrease of the fluorescence intensity was observed.

**Fig. 5:**
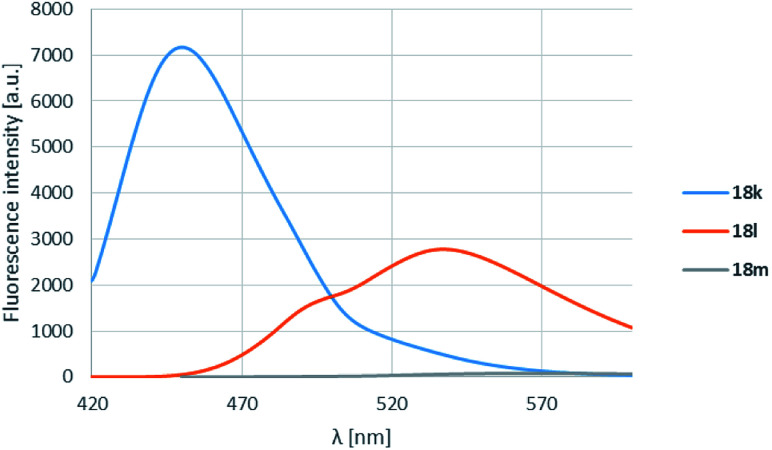
R^3^ substituent effect for boroisoquinolines 18.

The pyrrolidine substituent caused strikingly good fluorescence λ^max^-s depending on the substituent on the carbonyl group. It was found that the electron donating methyl group (18k) performed best, and as the electron withdrawal was increasing from the phenyl (18l) to the perfluorophenyl (18m) substituent, the fluorescence was shifted to the yellow range, but the intensity decreased significantly (Table 4, entries 3, 6, 12, Fig. 5).

The effect of the dehydrogenation was also studied, and it was found that in the case of the 6-methoxy derivatives (18a,c,e and 19a,c,e) the extension of the aromatic system was advantageous for the λ^max^-s and the Stokes-shifts (Table 4, entries 1 and 16, 4 and 18, 8 and 20). It should be noted, that by 19c a remarkable Stokes-shift (115 nm) was measured (Table 4, entry 18). Meanwhile, in the case of the 6,7-dimethoxyboroisoquiolines, the oxidized species were better only for the thienyl derivative (18f and 19f, Table 4, entries 9 and 21).

**Fig. 6:**
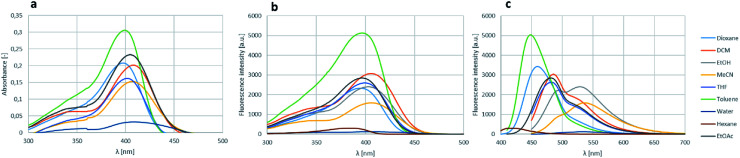
Solvent screen for the absorbance (a), excitation (b) and fluorescence (c) of 18l.

The effect of various solvents for the absorbance, excitation and fluorescence was also investigated on boroisoquinoline 18l (Fig. 6). The solvent screen contained hexane, toluene, ethyl acetate, dichloromethane, dioxane, tetrahydrofuran, ethanol, acetonitrile and water. It could be concluded, that the absorbance and excitation spectra remained similar in the different solvents, the absorption maximum was not shifting significantly (Fig. 6a and b, respectively). It should be noted, that the largest intensity was reached in toluene, ethanol, ethyl acetate and dioxane, while in water (or PBS buffer) the absorbance was practically ceased because of aggregation of the molecules. Meanwhile the solvents seemed to influence the fluorescence more significantly (Fig. 6b).

The measured intensities were similar for the different solvents than those by the absorbance measurements, but in most solvents except MeCN and EtOH the fluorescence maximum decreased to the range under 500 nm narrowing the room between the excitation and the fluorescence. The loss of absorbance and fluorescence in aqueous medium might be an advantage targeting a protein in an aprotic environment *e.g.* in the cell membrane, if the fluorescence gets enhanced reaching the target, while stay hidden in the aqueous environment outside the membrane.

**Fig. 7:**
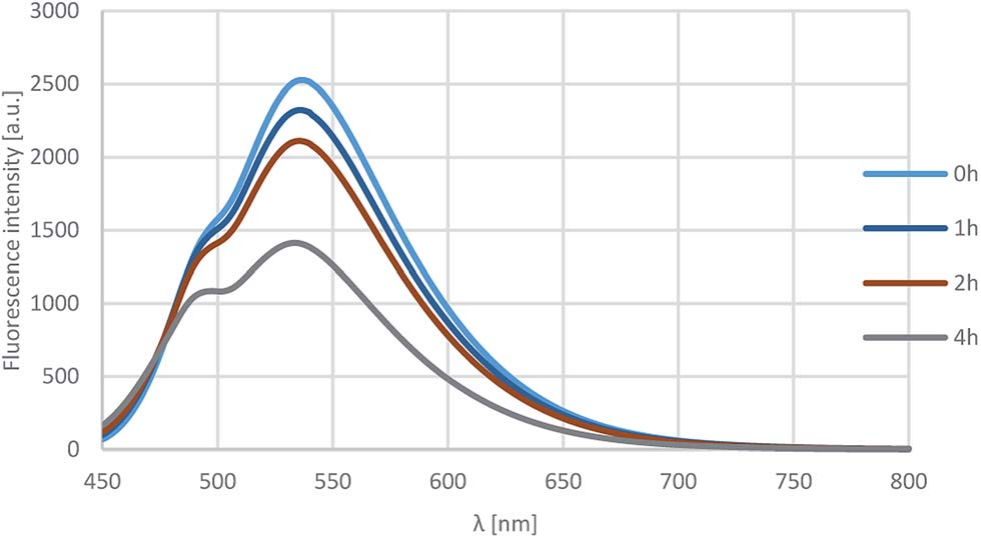
Results of the photostability test.

Furthermore, the photostability of compound 18l was investigated by measuring the fluorescence in acetonitrile after 0, 1, 2 and 4 h of irradiation with 360 nm light. As shown on Fig. 7, the fluorescence intensity was still around 60% after 4 h, that corresponds to outstanding photostability.

Compounds showing outstanding Stokes-shift were studied forward measuring molar absorptivity (*ε*) and quantum yield (*Φ*_F_) (Table 5). It was found, that some of the boroisoquinolines, particularly 18b and 18l showed more than 0.3 (entries 1, 4) and a remarkable quantum yield of 0.84 belonged to 18k (entry 2). On the contrary, the fully aromatic 19c (entry 10) and the perfluorophenyl derivatives (18g,h,m; entries 6–8) has lower *Φ*_F_ than 0.1, that might be explained with the dissipation of the electrons on the extended aromatic system, and the large, shielding substituents on the boron atom, respectively. Notably, the pyrrolidine substituent was advantageous for the *ε* and *Φ*_F_, but the larger substituents on either the carbonyl group or the boron atom decreased the above values, presumably because of the too extended electron system.

**Table 5:** Molar absorptivity and quantum yield for selected boroisoquinolines

Entry		*λ* ^max^_abs_ [nm]	*λ* ^max^_exc_ [nm]	*λ* ^max^_em_ [nm]	Δ*λ* [nm]	*ε* (*λ*^max^_abs_) [×10^4^ M^−1^ cm^−1^]	*Φ*_F_ a [−]
1	18b	321	339	431	110	2.20	0.32
2	18k	383	379	450	67	5.47	0.84
3	18d	361	380	483	122	3.06	0.18
4	18l	408	406	537	129	3.58	0.35
5	21l	417	415	544	127	3.71	0.17
6	18g	334	343	435	101	2.75	0.0024
7	18h	334	356	537	203	2.31	0.022
8	18m	411	410	565	154	2.55	0.037
9	22b	357	368	465	108	3.16	0.11
10	19c	378	372	487	109	2.47	0.016

a Quantum yields relative to quinin sulfate (dyes 18b,g, 22b) or to Coumarin 153 (dyes 18k,d,l,g,h,m, 19c, 21l, 22b).

**Scheme 4:**
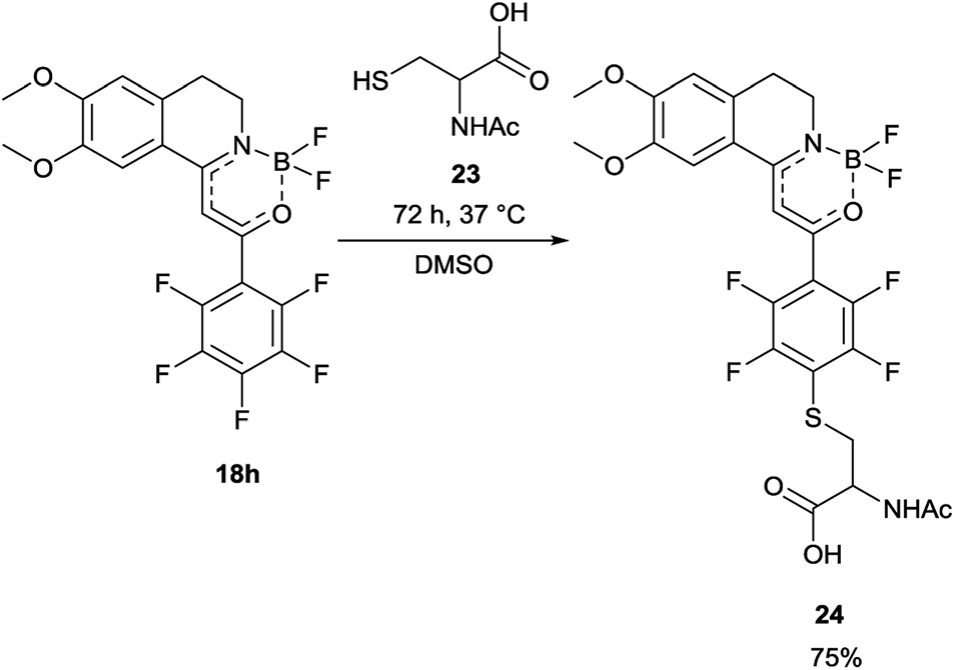
Reaction of 18h with *N*-acetylcysteine (23), as thiol-surrogate.

**Fig. 8:**
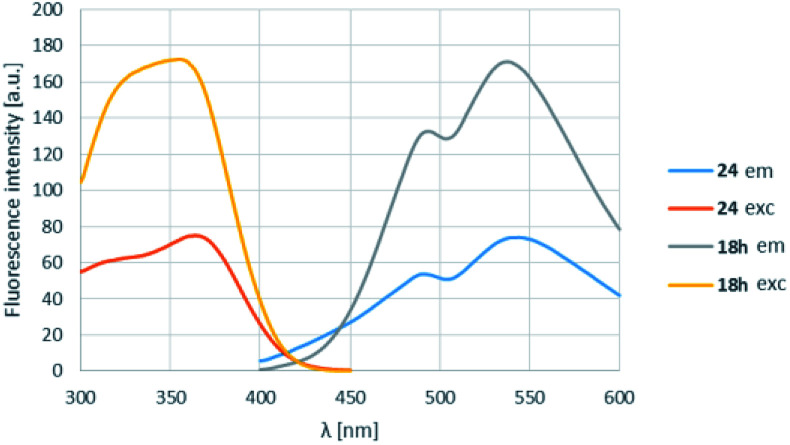
The change of the excitation and fluorescence spectra in the reaction of 18h with *N*-acetylcysteine (23).

In order to find a possible biological application, compound 18h was reacted with *N*-acetylcysteine (23), as a thiol-surrogate in DMSO at 37 °C. The full conversion was reached after 72 h and the adduct was isolated in a yield of 75% (Scheme 4). It was proven, that the thiol group reacted with a fluorine atom on the aromatic ring. The photophysical properties of the conjugate were investigated, and although the intensity of the fluorescence was decreased, the shape of the excitation and fluorescence curve and the Stokes-shift remained similar (Fig. 8). Although the reaction conditions are not yet compatible with the conditions in human cells, this preliminary result projects that after careful optimization these compounds might be applicable for the covalent labelling of cysteines in proteins.

## Conclusions

A new family of fluorophores, namely boroisoquinolines was developed, synthesized from 1-methyl-3,4-dihydroisoquinolines in a two or three step synthesis. The photophysical properties of the small library were extensively studied, and outstanding Stokes shifts with good molar absorptivity values were observed. Some of the dyes (18b,k,l) showed appreciable to excellent quantum yields with good photostability. The solvent screening results indicated low fluorescence in aqueous medium that might serve as an advantage using the compounds in aprotic conditions *e.g.* in cell membranes. We believe that upon further optimization these fluorescent dyes might be good choices for visualization of the labelling of the appropriate proteins (*e.g.* CB_2_).

## Conflicts of interest

There are no conflicts to declare.

## Supplementary Material

RA-008-C8RA08241C-s001

RA-008-C8RA08241C-s002
