# Supplementary material for: Synthesis and fluorescent properties of boroisoquinolines, a new family of fluorophores
Source: RSC Adv. 2018 Nov 15;8(67):38598–605. doi: 10.1039/c8ra08241c (PMC9090577; doi:10.1039/c8ra08241c)
Supplement: RA-008-C8RA08241C-s001 [file RA-008-C8RA08241C-s001.pdf]

Electronic Supplementary Material (ESI) for RSC Advances.
This journal is © The Royal Society of Chemistry 2018

Synthesis and fluorescence properties of boroisoquinolines, a new family of fluorophores

Dénes Sóvári,[1] Attila Kormos,[2] Orsolya Demeter,[2] András Dancsó,[3] Mátyás Milen,[3] Péter Ábrányi-Balogh[1,*]

[1]Hungarian Academy of Sciences, Research Centre for Natural Sciences, Institute of Organic Chemistry, Medicinal Chemistry Research Group, 1519 Budapest, POB 286, Hungary

[2]Hungarian Academy of Sciences, Research Centre for Natural Sciences, Institute of Organic Chemistry, Chemical Biology Research Group, 1519 Budapest, POB 286, Hungary

[3]Egis Pharmaceuticals Plc., Directorate of Drug Substance Development, 1475 Budapest, P.O.B. 100, Hungary

*To whom correspondence should be addressed. Tel.: +36 1 3826961; e-mail: abranyi-balogh.peter@ttk.mta.hu

Table of Contents

Synthesis and fluorescence properties of boroisoquinolines, a new family of fluorophores 1

1. Experimental 2
2. Optical spetroscopy data for boroisoquinolines 25
3. X-Ray diffraction data for boroisoquinolines **18f** and **19c** 26
4. $^{1}H/^{13}C$ NMR spectra 27
4. References 58

1. Experimental

General

All melting points were determined on a Jasco SRS OptiMelt apparatus and are uncorrected. ^{1}H and ^{13}C NMR spectra were recorded in $CDCl_3$ solution at room temperature, on a Varian Unity Inova 500 spectrometer (500 and 125 MHz for ^{1}H and APT NMR spectra, respectively), and on a Varian Unity Inova 300 spectrometer (282 MHz for ^{19}F NMR spectra), with the deuterium signal of the solvent as the lock and TMS as the internal standard. Chemical shifts (δ) and coupling constants (J) are given in ppm and Hz, respectively. High resolution mass spectra were recorded on a Waters Q-TOF Premier mass spectrometer in positive ESI ionization mode. The microwave reactions were conducted in an Anton Paar Monowave 300 microwave reactor. The reactions were followed by analytical thin layer chromatography on silica gel 60 F_{254} and HPLC–MS chromatography with a Shimadzu LCMS-2020 device using a Reprospher 100 C18 (5 µm; 100x3mm) column and positive-negative double ion source (DUIS±) with a quadrupole MS analysator in a range of 50−1000 m/z. All reagents were purchased from commercial sources. Analytical samples of new compounds were obtained by trituration or recrystallization from the solvents or solvent mixtures given below in parentheses. Single crystal X-ray measurements were carried on a Rigaku R-Axis Rapid imaging plate area detector with graphite monochromated Cu-Kα radiation.

Photophysical measurements

The fluorescence and absorbance measurements were carried out on a Jasco FP8300 spectrofluorometer, in a standard cell quartz cuvette with 1 cm light path length. The widths of the excitation slit and the emission slit were both set to 2.5 nm with the scanning speed at 1000 nm/min. Pure solvents were used as blank correction. We used acetonitrile as solvent if not mentioned else, the concentration was 5 µM or 0.5 µM.

The quantum yields were determined by recording the fluorescence spectra of a series of different concentrations. The gradient of the integrated fluorescence intensities plotted against absorbance at the excitation wavelength was used for the calculation of the quantum yields:

$$\Phi_x = \Phi_{St} \cdot \left(\frac{Grad_x}{Grad_{St}}\right) \cdot \left(\frac{\eta_x^2}{\eta_{St}^2}\right)$$

The **18d**,**h**,**l**,**k**,**m** and **21l** compounds were excited at 380 nm, Coumarin-153 was used as reference (Φ_F^{EtOH}=0.55). The **18b** and **22b** compounds were excited at 339 nm, quinine sulfate was used as reference ($\Phi_{F(0.5\ M\ H_2SO_4)}$=0.54) During the computations we used refraction coefficients from literature.[1]

The solvent screen measurement was carried out with 5 μM solutions of the **18l** boroisoquinoline in acetonitrile, 1,4-dioxane, water, toluene, dichloromethane, tetrahydrofuran, ethanol, hexane and ethyl acetate.

The photostability measurement was carried out with 5 μM solutions of compound **18l** in acetonitrile. We used 8 W, 366 nm emitting UV lamp, and measured the emission spectrum in every hour for 4 hours. During the photophysical measurments the solutions were excited at 380 nm.

General procedure for the preparation of *N*-phenethylacetamides

In a three-necked round bottom flask 5.0 g 2-(3,4-dimethoxyphenyl)ethyl-amine or 4.2 g 2-(3-dimethoxyphenyl)ethan-1-amine or 5.6 g 2-(3-bromophenyl)ethan-1-amine or 3.9 g 2-(3-fluorophenyl)ethan-1-amine (28 mmol) and 5.1 mL triethyl-amine (3.8 g, 36.4 mmol, 1.3 equiv) was dissolved in 50 mL dichloromethane. To this solution was added dropwise 2.4 mL acetyl chloride (2.6 g, 34.0 mmol, 1.2 equiv) and the mixture was stirred at room temperature for 30 minutes. The reaction mixture was quenched with 50 mL water. The layers were separated, and the organic layer was washed with 40 mL 1 M HCl and then 40 mL 5% Na_2CO_3 solution, dried over $MgSO_4$, filtered and evaporated under reduced pressure.

N-[2-(3,4-Dimethoxyphenyl)ethyl]acetamide[2]

Yield: 6.1 g (98%), light yellow crystals, m.p. 98−99 °C (EtOAc-hexane) (lit. 100−101 °C), ^{1}H NMR (500 MHz, $CDCl_3$): δ 6.81 (d, *J* = 7.8 Hz, 1H, ArH), 6.73 (d, *J* = 8.2 Hz, 2H, ArH), 5.56 (s, 1H, NH), 3.87 (s, 3H, OCH_3), 3.86 (s, 3H, OCH_3), 3.49 (q, *J* = 6.7 Hz, 2H, CH_2), 2.76 (t, *J* = 7.0 Hz, 2H, CH_2), 1.94 (s, 3H, CH_3) ppm.

N-[2-(3-Methoxyphenyl)ethyl]acetamide[3]

Yield: 4.6 g (86%); yellow oil; ^{1}H NMR (500 MHz, $CDCl_3$): δ 7.21 (t, *J* = 7.8 Hz, 1H, ArH), 6.79−6.74 (m, 2H, ArH), 6.72 (s, 1H, ArH), 5.65 (s, 1H, NH), 3.78 (s, 3H, OCH_3), 3.49 (q, *J* = 6.8 Hz, 2H, CH_2), 2.77 (t, *J* = 7.0 Hz, 2H, CH_2), 1.92 (s, 3H, CH_3) ppm.

N-[2-(3-Bromophenyl)ethyl]acetamide[4]

Yield: 5.6 g (83%), brown oil; ^{1}H NMR (500 MHz, $CDCl_3$) δ 7.34 (d, *J* = 11.3 Hz, 1H, ArH), 7.19 – 7.14 (m, 1H, ArH), 7.11 (d, *J* = 7.6 Hz, 1H, ArH), 5.65 (s, 1H, NH), 3.47 (q, *J* = 13.2. 6.8 Hz, 1H, CH_2), 2.78 (t, *J* = 7.0 Hz, 1H, CH_2), 1.93 (s, 3H, CH_3) ppm.

N-[2-(3-Fluorophenyl)ethyl]acetamide[3]

Yield: 4,2 g (83%), yellow oil; ^{1}H NMR (500 MHz, $CDCl_3$) δ 7.25 (dd, *J* = 13.9. 7.9 Hz, 1H, ArH), 6.96 (d, *J* = 7.6 Hz, 1H, ArH), 6.90 (d, *J* = 6.5 Hz, 1H, ArH), 5.63 (s, 1H, NH), 3.49 (dd, *J* = 13.1. 6.8 Hz, 2H, CH_2), 2.80 (t, *J* = 7.0 Hz, 2H, CH_2), 1.93 (s, 3H, CH_3) ppm.

General procedure for the preparation of 1-methyl-3,4-dihydroisoquinolines (16a,b)

Method A

In a three-necked round bottom flask 5.5 g *N*-[2-(3,4-dimethoxyphenyl)ethyl]acetamide or 4.8 g of *N*-[2-(3-methoxyphenyl)ethyl]acetamide (24.7 mmol) was dissolved in 50 mL toluene and 2.3 mL phosphorus oxychloride (24.7 mmol, 1 equiv.) was added dropwise. The solution was stirred at reflux temperature for 1 h. The reaction mixture was cooled to room temperature, and the solvent was removed *in vacuo*. The brown crystallic hydrochloride salt was triturated with diethyl ether, and filtered. The remaining solid was suspended in 50 mL of dichloromethane, and 200 mL concd. $NaHCO_3$ solution was added. The layers were separated, and the aqueous phase was washed with 2 × 30 mL dichloromethane. The combined organic phases were dried (Na_2SO_4), filtered and the solvent was removed *in vacuo*.

6-Methoxy-1-methyl-3,4-dihydroisoquinoline (**16a**)[5]

Yield: 3.2 g (73%); brown oil; ^{1}H NMR (500 MHz, $CDCl_3$): δ 7.41 (d, *J* = 8,5 Hz, 1H, ArH), 6.77 (dd, J_1 = 8.4 Hz, J_2 = 2.1 Hz, 1H, ArH), 6.69 (s, 1H, ArH), 3.82 (s, 3H, OCH_3), 3.62 (t, *J* = 6.9 Hz, 2H, CH_2), 2.67 (t, *J* = 7.4 Hz, 2H, CH_2), 2.33 (s, 3H, CH_3) ppm.

6,7-Dimethoxy-1-methyl-3,4-dihydroisoquinoline (**16b**)[6]

Yield: 4.8 g (95%); pale brown crystals; m.p. 101–102 °C (Et_2O) (lit. 102–103°C); ^{1}H NMR (500 MHz, $CDCl_3$): δ 7.00 (s, 1H, ArH), 6.69 (s, 1H, ArH), 3.92 (s, 3H, OCH_3), 3.91 (s, 3H, OCH_3), 3.63 (t, *J* = 7.6 Hz, 2H, CH_2), 2.64 (t, *J* = 7.5 Hz, 2H, CH_2), 2.36 (s, 3H, CH_3) ppm.

Method B

In a three-necked round bottom flask 4.6 g *N-[2-(3-bromophenyl)ethyl]acetamide* or 3.4 g *N-[2-(3-fluorophenyl)ethyl]acetamide* (18.9 mmol) was dissolved in 100 mL dichloromethane and 1.8 mL oxalyl chloride (2.6 g, 20.7 mmol, 1.1 equiv) was added dropwise under nitrogen atmosphere. The solution was stirred at room temperature for 30 minutes. Then the reaction mixture was cooled below –10 °C, and 3.7 g $FeCl_3$ (22.7 mmol, 1.2 equiv) was added. The mixture was stirred at room temperature overnight. Aqueous 2M HCl (10 mL, 20.0 mmol) was added to quench the reaction. The mixture was stirred at room temperature for 1 h, then the layers were separated. The organic layer was washed with brine, and dried over $MgSO_4$),

filtered, and the solvent was removed *in vacuo*. The oxalyl adduct was dissolved in 180 mL methanol, 9.5 mL concentrated sulphuric acid was added, and stirred at reflux overnight. The next morning the reaction mixture was allowed to cool room temperature, and the solvent was removed *in vacuo*. The crude material was dissolved in ethyl acetate, and washed with water. The aqueous layer was basified with 10 % sodium hydroxide solution, and extracted with ethyl acetate. The combined organic phases were dried ($MgSO_4$), filtered and the solvent was removed *in vacuo*.

6-Bromo-1-methyl-3,4-dihydroisoquinoline (**16c**)[7]

Yield: 3.5 g (82%), brown oil; ^{1}H NMR (500 MHz, $CDCl_3$) δ 7.45 – 7.37 (m, 2H, ArH), 7.32 (d, *J* = 8.4 Hz, 2H, ArH), 3.64 (s, *J* = 7.5. 1.3 Hz, 2H, CH_2), 2.72 – 2.60 (m, 2H, CH_2), 2.35 (s, 3H, CH_3) ppm; ^{13}C NMR (125 MHz, $CDCl_3$) δ 163.4 (C=), 139.5 (C=), 130.4 (=CH), 130.0 (=CH), 128.3 (C=), 126.8 (=CH), 124.6 (C=), 46.7 (CH_2), 25.8 (CH_2), 23.2 (CH_3) ppm.

6-Fluoro-1-methyl-3,4-dihydroisoquinoline (**16d**)[8]

Yield: 2.3 g (76%), tawny oil; ^{1}H NMR (500 MHz, $CDCl_3$) δ 7.56 (dd, *J* = 8.5. 5.5 Hz, 1H, ArH), 7.01 (td, *J* = 8.5. 2.5 Hz, 1H, ArH), 6.93 (dd, *J* = 8.6. 2.2 Hz, 1H, ArH), 3.73 (dd, *J* = 12.8. 5.2 Hz, 2H, CH_2), 2.83 – 2.76 (m, 2H, CH_2), 2.49 (s, 3H, CH_3) ppm; ^{13}C NMR (125 MHz, $CDCl_3$) δ 165.1 (=CF), 140.0 (C=), 135.8 (C=), 128.4 (d, *J* =9.6 Hz, =CH), 124.6 (C=), 114.4 (d, *J* =22.1 Hz, =CH), 113.7 (d, *J* =22.1 Hz, =CH), 44.3 (CH_2), 25.5 (CH_2), 21.6 (CH_3) ppm; ^{19}F NMR (282 MHz, $CDCl_3$) δ -130.01 (s, 1F, ArF) ppm.

Synthesis of 1-methyl-6-(pyrrolidin-1-yl)-3,4-dihydroisoquinoline (16e)

Method A: Buchwald-Hartwig coupling

Into a sealed tube 10 mL anhydrous toluene, 0.5 g 6-bromo-1-methyl-3,4-dihydroisoquinoline (**16c**) (2.3 mmol), 0.025 g $Pd(OAc)_2$ (0.1 mmol, 0.05 equiv), 0.067 g JohnPhos ligand (0.2 mmol, 0.1 equiv) was measured. The tube was flushed with argon, then 0.31 g potassium *tert*-butoxide (2.8 mmol, 1.25 equiv) was added. The tube was flushed again with argon and 0.55 mL pyrrolidine (0.5 g, 7.0 mmol, 3 equiv) was added. The tube was heated to 80 °C, then all of the reagents except potassium *tert*-butoxide were added again. After 30 minutes at 80 °C, the reaction mixture was filtered through celite. The crude product was purified by flash column chromatography on alumina using dichloromethane/methanol.

Yield: 0.34 g (40%), brown oil.

Method B

In a round bottom flask 0.1 g 6-fluoro-1-methyl-3,4-dihydroisoquinoline (**16d**) was dissolved in 2 mL pyrrolidine (1.7 g, 123 mmol, 205 equiv.). The reaction mixture was refluxed for 18h,

then concentrated *in vacuo*, and the residue was purified by flash column chromatography on alumina using dichloromethane/methanol.

Yield: 0.05 g (40%), brown oil.

Method C

In a sealed tube 0.1 g 6-fluoro-1-methyl-3,4-dihydroisoquinoline (**16d**) (0.6 mmol) and 2 mL pyrrolidine (1.7 g, 123 mmol, 205 equiv) were dissolved in 6 mL acetonitrile. The reaction mixture was heated at 80 °C for 9 days. The residue was purified by flash column chromatography on alumina using dichloromethane/methanol.

Yield: 0.05 g (42%), brown oil.

Method D

In a sealed tube 4.4 g 6-fluoro-1-methyl-3,4-dihydroisoquinoline (**16d**) (27.0 mmol) and 10 mL pyrrolidine (11.5 g, 162 mmol, 6 equiv) were dissolved in 10 mL dioxane. The reaction mixture was heated at 80 °C for 3 days. The residue concentrated under reduced pressure, and purified with steam distillation. After all of the excess pyrrolidine was distilled, the leftover was dissolved in aqueous HCl, then extracted with dicholormethane (5x50 mL). For NMR purpose the crude product purified with reverse phase column chromatography using acetonitrile/water.

Yield: 4.0 g (70 %), yellow crystals, m. p. 47-48 °C (water-MeCN); ^{1}H NMR (500 MHz, $CDCl_3$) δ 7.45 (d, *J* = 8.9 Hz, 1H, ArH), 6.41 (dd, *J* = 8.9. 2.3 Hz, 1H, ArH), 6.28 (s, 1H, ArH), 3.66 (t, *J* = 6.8 Hz, 2H, CH_2), 3.38 – 3.28 (m, 4H, CH_2), 2.84 (t, *J* = 7.6 Hz, 2H, CH_2), 2.59 (s, 3H, CH_3), 1.97 (dt, *J* = 17.0. 6.7 Hz, 4H, CH_2) ppm; ^{13}C NMR (125 MHz, $CDCl_3$) δ 170.0 (C=), 152.6 (C=), 139.7 (C=), 131.4 (=CH), 113.3 (C=), 110.8 (=CH), 110.4 (=CH), 47.9 (CH_2), 41.4 (CH_2), 26.6 (CH_2), 25.2 (CH_2), 19.2 (CH_3) ppm. $[M+H]^{+}_{measured}$ = 215.1560, calcd. for $C_{14}H_{19}N_2$: 215.1543.

General procedure for the preparation of 1-methylidene-3,4-dihydroisoquinolines (17)

Method A

In a three-necked round bottom flask 20 mL anhydrous tetrahydrofurane was cooled below – 70 °C, under nitrogen atmosphere. Then 6.25 mmol (2.5 equiv.) *n*-BuLi was added dropwise [except **17g**,**h** when 3 mmol (1.2 equiv.) was used], then the corresponding 2.5 mmol isoquinoline in tetrahydrofuran solution (0.44 g **16a**, 0.51 g **16b**) was added dropwise. The reaction mixture was then stirred for 30 minutes, and the appropriate ester (6.25 mmol, 2.5 equiv.) was added dropwise in 10 mL tetrahydrofuran [**17a**,**b**: ethyl acetate (0.55 g, 0.62 ml); **17c**,**d**: ethyl benzoate (0.94 g, 0.89 ml); **17i**: ethyl 4-nitrobenzoate (1.2 g); **17e**,**f**: ethyl 2-

thiophenecarboxylate (0.98 g, 0.84 ml); **17g,h**: ethyl pentafluorbenzoate (1.5 g, 1.1 ml)]. Then the reaction mixture was allowed to warm to room temperature, and stirred for 1h. The solution was quenched with 30 mL water, and extracted with 80 mL ethyl acetate. The aqueous layer was washed with ethyl acetate (2x30 mL) and dichloromethane (2x30 mL). The combined organic phases were dried ($MgSO_4$), filtered and evaporated under reduced pressure. The residue was purified by flash column chromatography on silica gel using hexane/ethyl acetate.

(Z)-1-(6-methoxy-3,4-dihydroisoquinolin-1(2H)-ylidene)propan-2-one (**17a**)[9]

Yield: 0.25 g (46%); tawny crystals; m. p. 87–88 °C (hexane-EtOAc); ^{1}H NMR (500 MHz, $CDCl_3$): δ 12.23 (s, 1H, NH), 7.61 (d, J = 8.7 Hz, 1H, ArH), 6.80 (dd, J_1 = 8.7 Hz, J_2 = 2.3 Hz, 1H, ArH), 6.70 (d, J = 2.5 Hz, 1H, ArH), 5.56 (s, 1H, HC=), 3.83 (s, 3H, OCH_3), 3.44 (td, J_1 = 6.25 Hz, J_2 = 2.9 Hz, 2H, CH_2), 2.87 (t, J = 6.4 Hz, 2H, CH_2), 2.12 (s, 3H, CH_3) ppm.

(Z)-1-(6,7-dimethoxy-3,4-dihydroisoquinolin-1(2H)-ylidene)propan-2-one (**17b**)[9]

Yield: 0.32 g (50%); yellow crystals; m. p. 137–138 °C (hexane-EtOAc); ^{1}H NMR (500 MHz, $CDCl_3$): δ 11.23 (s, 1H, NH), 7.13 (s, 1H, ArH), 6.67 (s, 1H, ArH), 5.52 (s, 1H, HC=), 3.93 (s, 3H, OCH_3), 3.91 (s, 3H, OCH_3), 3.44 (td, J_1 = 6.5 Hz, J_2 = 3.3 Hz, 2H, CH_2), 2.83 (t, J = 6.5. 2H, CH_2), 2.14 (s, 3H, CH_3) ppm.

(Z)-2-(6-methoxy-3,4-dihydroisoquinolin-1(2H)-ylidene)-1-phenylethan-1-one (**17c**)[10]

Yield: 0.35 g (51%); brown oil; ^{1}H NMR (500 MHz, $CDCl_3$): δ 11.81 (s, 1H, NH), 7.93 (dd, J_1 = 6.4 Hz, J_2 = 3.0 Hz, 2H, ArH), 7.76 (d, J = 8.7 Hz, 1H, ArH), 7.48–7.39 (m, 3H, ArH), 6.86 (dd, J = 8.6 Hz, 1H, ArH), 6.75 (d, J = 2.2 Hz, 1H, ArH), 6.26 (s, 1H, HC=), 3.86 (s, 3H, OCH_3), 3.54 (td, J_1 = 6.5 Hz, J_2 = 3.0 Hz, 2H, CH_2), 2.94 (t, J = 6.4 Hz, 2H, CH_2) ppm.

(Z)-2-(6,7-dimethoxy-3,4-dihydroisoquinolin-1(2H)-ylidene)-1-phenylethan-1-one (**17d**)[9]

Yield: 0.41 g (53%); yellow crystalles; m. p. 137-139 °C (lit.19 135–136 >°C); ^{1}H NMR (500 MHz, $CDCl_3$): δ 11.81 (s, 1H, NH), 7.92 (d, J = 3.3 Hz, 2H, ArH), 7.45 – 7.42 (m, 3H, ArH), 7.25 (s, 1H, ArH), 6.71 (s, 1H, ArH), 6.19 (s, 1H, HC=), 3.95 (s, 3H, OCH_3), 3.93 (s, 3H, OCH_3), 3.53 (td, J_1 = 6.5 Hz, J_2 = 3.3 Hz, 2H, CH_2), 2.89 (d, J = 6.5 Hz, 2H, CH_2) ppm.

(Z)-2-(6-methoxy-3,4-dihydroisoquinolin-1(2H)-ylidene)-1-(thiophen-2-yl)ethan-1-one (**17e**) [9]

Yield: 0.41 g (58%); brown oil; ^{1}H NMR (500 MHz, $CDCl_3$): δ 11.47 (s, 1H, NH), 7.75 (d, J = 8.8 Hz, 1H, ArH), 7.64 (d, J = 3.6 Hz, 1H, ArH), 7.45 (d, J = 4.9 Hz, 1H, ArH), 7.09 (dd, J_1 = 4.7 Hz, J_2 = 4.0 Hz, 1H, ArH), 6.87 (dd, J_1 = 8.7 Hz, J_2 = 2.5 Hz, 1H, ArH), 6.75 (d, J = 2.0 Hz, 1H, ArH), 6.16 (s, 1H, HC=), 3.87 (s, 3H, CH_3), 3.53 (td, J_1 = 6.8 Hz, J_2 = 2.5 Hz, 2H, CH_2), 2.93 (t, J = 6.5 Hz, 2H, CH_2) ppm.

(Z)-2-(6,7-dimethoxy-3,4-dihydroisoquinolin-1(2H)-ylidene)-1-(thiophen-2-yl)ethan-1-one **(17f)** [9]

Yield: 0.37 g (48%); yellow crystals; m. p. 151–152 °C (hexane-EtOAc); ^{1}H NMR (500 MHz, $CDCl_3$): δ 11.45 (s, 1H, NH), 7.62 (s, 1H, ArH), 7.44 (d, *J* = 3.5 Hz, 1H, ArH), 7.23 (s, 1H, ArH), 7.08 (t, *J* = 4.3 Hz, 1H, ArH), 6.70 (s, 1H, ArH), 6.09 (s, 1H, HC=), 3.96 (s, 3H, OCH_3), 3.96 (s, 3H, OCH_3), 3.50 (t, *J* = 7.0 Hz, 2H, CH_2), 2.88 (t, *J* = 6.3 Hz, 2H, CH_2) ppm.

(Z)-2-(6-methoxy-3,4-dihydroisoquinolin-1(2H)-ylidene)-1-(perfluorophenyl)ethan-1-one **(17g)**

Instead of 2.5 equiv. we used 1.2 equiv. reagents.

Yield: 0.28 g (12%); white crystals; m. p. 126–128 °C (water-MeCN); ^{1}H NMR (500 MHz, $CDCl_3$) δ 11.55 (s, 1H, NH), 7.62 (d, *J* = 8.7 Hz, 1H, ArH), 6.80 (t, *J* = 14.0 Hz, 1H, ArH), 6.75 (s, 1H, ArH), 5.72 (s, 1H, HC=), 3.85 (s, 3H, OCH_3), 3.56 (s, 2H, CH_2), 2.96 (t, *J* = 6.1 Hz, 2H, CH_2) ppm; ^{13}C NMR (125 MHz, $CDCl_3$) δ 177.0 (C=O), 162.7 (C=), 159.9 (C=), 144.9 (C=), 143.8 (d, J = 241.6 Hz, =CF), 140.1 (=CF), 138.9 (C=), 136.5 (=CF), 128.0 (=CH), 120.5 (C=), 113.4 (=CH), 113.1 (=CH), 91.8 (=CH), 55.4 (OCH_3), 38.7 (CH_2), 28.2 (CH_2) ppm; ^{19}F NMR (282 MHz, $CDCl_3$) δ -142.47 (dd, *J* = 23.4, 8.2 Hz, 2F, ArF), -155.13 (t, *J* = 20.7 Hz, 1F, ArF), -162.18 (dt, *J* = 23.5, 8.3 Hz, 2F, ArF) ppm; $[M+H]^+_{measured}$ = 370.0858. calcd, $C_{18}H_{13}F_5NO_2$: 370.0861.

(Z)-2-(6,7-dimethoxy-3,4-dihydroisoquinolin-1(2H)-ylidene)-1-(perfluorophenyl)ethan-1-one **(17h)**

Instead of 2.5 equiv. we used 1.2 equiv. reagents.

Yield: 0.58 g (57%), yellow crystals; m. p. 178–180 °C (MeCN); ^{1}H NMR (500 MHz, $CDCl_3$) δ 11.54 (s, 1H, NH), 7.09 (s, 1H, ArH), 6.71 (s, 1H, ArH), 5.66 (s, 1H, HC=), 3.93 (s, 3H, OCH_3), 3.89 (s, 3H, OCH_3), 3.57-3.54 (m, 2H, CH_2), 2.92 (t, *J* = 14.0. 7.3 Hz, 2H, CH_2) ppm; ^{13}C NMR (125 MHz, $CDCl_3$) δ 177.1 (C=O), 159.8 (C=), 152.5 (C=), 148.2 (C=), 144.85 (C=), 143.8 (d, *J* = 256.9 Hz, =CF), 141.1 (d, *J* = 255.0 Hz, =CF), 137.5 (d, *J* =253.1 Hz, =CF), 130.9 (C=), 120.3 (C=), 120.1 (C=), 117.7 (C=), 110.8 (=CH), 108.7 (=CH), 91.8 (=CH), 56.2 (OCH_3), 56.1 (OCH_3), 38.8 (CH_2), 27.5 (CH_2) ppm; ^{19}F NMR (282 MHz, $CDCl_3$) δ -142.49 (dd, *J* = 23.5, 8.0 Hz, 2F, ArF), -155.14 (t, *J* = 20.7 Hz, 1F, ArF), -161.89 – -162.35 (m, 2F, ArF) ppm.; $[M+H]^+_{measured}$ = 400.0970. calcd. for $C_{19}H_{15}F_5NO_3$: 400.0972.

(Z)-2-(6-methoxy-3,4-dihydroisoquinolin-1(2H)-ylidene)-1-(4-nitrophenyl)ethan-1-one **(17i)**

Yield: 0.043 g (5%); orange crystals; m. p. 152–153 °C (hexane); ^{1}H NMR (500 MHz, $CDCl_3$): δ 11.97 (s, 1H, NH), 8.27 (d, *J* = 8.6 Hz, 2H, ArH), 8.06 (d, *J* = 8.6 Hz, 2H, ArH),

7.77 (d, J = 8.7 Hz, 1H, ArH), 6.89 (dd, J_1 = 8.7 Hz, J_2 = 2.2 Hz, 1H, ArH), 6.79 (d, J = 2.1 Hz, 1H, ArH), 6.24 (s, 1H, HC=), 3.89 (s, 3H, OCH_3), 3.60 (td, J_1 = 6.6 Hz, J_2 = 3.3 Hz, 2H, CH_2), 2.98 (t, J = 6.6 Hz, 2H, CH_2) ppm.

Method B

In a three-necked round bottom flask 20 mL anhydrous tetrahydrofuran and 0.88 mL diisopropylamine (0.63 g, 6.25 mmol, 2.5 equiv.) was cooled below –70 °C, under nitrogen atmosphere. Then 6.25 mmol (2.5 equiv.) *n*-BuLi was added dropwise [except **17m** when 3 mmol (1.2 equiv.) was used], the reaction mixture was stirred for 1h. Then the corresponding 2.5 mmol isoquinoline in tetrahydrofuran solution (0.51 g **16b**, 0.54 g **16e**) was added dropwise. After it the reaction mixture was stirred for 1h, then the appropriate ester (6.25 mmol, 2.5 equiv.) was added dropwise in 10 mL tetrahydrofuran [**17k**: ethyl acetate (0.55 g, 0.62 ml); **17l**: ethyl benzoate (0.94 g, 0.89 ml); **17j**: ethyl 4-nitrobenzoate (1.2 g); **17m**: ethyl pentafluorbenzoate (1.5 g, 1.1 ml)]. Then the reaction mixture was allowed to warm to room temperature, and stirred for 1h. The solution was quenched with 30 mL water, and extracted with 80 mL ethyl acetate. The aqueous layer was washed with ethyl acetate (2x30 mL) and dichloromethane (2x30 mL). The combined organic phases were dried ($MgSO_4$), filtered and evaporated under reduced pressure. The residue was purified by flash column chromatography on silica gel using hexane/ethyl acetate.

(Z)-2-(6,7-dimethoxy-3,4-dihydroisoquinolin-1(2H)-ylidene)-1-(4-nitrophenyl)ethan-1-one (**17j**)

Yield: 0.54 g (54%) [0.030 g (3%)]; orange crystals; m. p. 187–188 °C (MeCN); ^{1}H NMR (500 MHz, $CDCl_3$): δ 11.96 (s, 1H, NH), 8.26 (d, J = 5.8 Hz, 2H, ArH), 8.04 (d, J = 6.1 Hz, 2H, ArH), 7.23 (s, 1H, ArH), 6.73 (s, 1H, ArH), 6.15 (s, 1H, HC=), 3.96 (s, 3H, OCH_3), 3.95 (s, 3H, OCH_3), 3.57 (s, 2H CH_2), 2.92 (s, 2H, CH_2) ppm.

(Z)-1-(6-(pyrrolidin-1-yl)-3,4-dihydroisoquinolin-1(2H)-ylidene)propan-2-one (**17k**)

Yield: 0.27 g (41%) [0.17 g (26%)]; yellow crystals; m. p. 108–110 °C ($CDCl_3$); ^{1}H NMR (500 MHz, $CDCl_3$): δ 11.25 (s, 1H, NH), 7.54 (d, J = 8.8 Hz, 1H, ArH), 6.43 (dd, J = 8.8, 2.1 Hz, 1H, ArH), 6.29 (s, 1H, ArH), 5.52 (s, 1H, HC=), 3.44 (td, J = 6.6, 3.0 Hz, 2H, CH_2), 3.35-3.31 (m, 4H, CH_2), 2.84 (t, J = 6.5 Hz, 2H, CH_2), 2.11 (s, 3H, CH_3), 2.04-2.00 (m, 4H, CH_2) ppm; ^{13}C NMR (125 MHz, $CDCl_3$) δ 193.4 (C=O), 157.8 (C=), 149.1 (C=), 137.9 (C=), 126.7 (=CH), 115.2 (C=), 109.7 (=CH), 87.5 (=CH), 47.0 (CH_2), 38.2 (CH_2), 28.7 (CH_3), 28.6 (CH_2), 24.9 (CH_2) ppm; $[M+H]^+_{measured}$ = 257.1660, calcd. for $C_{16}H_{21}N_2O$: 257.1650

(Z)-1-phenyl-2-(6-(pyrrolidin-1-yl)-3,4-dihydroisoquinolin-1(2H)-ylidene)ethan-1-one (**17l**)

Yield: 0.56 g (72%) [0.21 g (27%)]; yellow crystlas; m. p. 137–138 °C (MeCN); ^{1}H NMR (500 MHz, $CDCl_3$): δ 11.82 (s, 1H, NH), 7.94 (dd, *J* = 6.4, 2.8 Hz, 2H, ArH), 7.68 (d, *J* = 8.8 Hz, 1H, ArH), 7.41 (dd, *J* = 6.3, 2.9 Hz, 3H, ArH), 6.47 (dd, *J* = 8.4, 2.1 Hz, 1H, ArH), 6.32 (d, *J* = 1.6 Hz, 1H, ArH), 6.22 (s, 1H, HC=), 3.53 (t, *J* = 5.6 Hz, 2H, CH_2), 3.35 (t, *J* = 6.5 Hz, 4H, CH_2), 2.89 (t, *J* = 6.6 Hz, 2H, CH_2), 2.04 – 2.00 (m, 4H, CH_2) ppm; ^{13}C NMR (125 MHz, $CDCl_3$) δ 187.2 (C=O), 159.9 (C=), 159.5 (C=), 149.8 (C=), 141.7 (C=), 138.4 (C=), 129.9 (=CH), 127.61 (=CH), .128.0 (=CH), 127.4 (=CH), 126.8 (=CH), 110.27 (=CH), 110.24 (=CH), 85.4 (=CH), 47.1 (CH_2), 47.5 (CH_2), 38.9 (CH_2), 29.0 (CH_2), 25.4 (CH_2) ppm; $[M+H]^+_{measured}$ = 319.1800, calcd. for $C_{21}H_{23}N_2O$: 319.1805.

(Z)-1-(perfluorophenyl)-2-(6-(pyrrolidin-1-yl)-3,4-dihydroisoquinolin-1(2H)-ylidene)ethan-1-one (**17m**)

Instead of 2.5 equiv. we used 1.2 equiv. reagents.

Yield: 0.2 g (20%) [0.16 g (16%)]; yellow crystals; m. p. 191–192 °C ($CDCl_3$); ^{1}H NMR (500 MHz, $CDCl_3$): δ 11.49 (s, 1H, NH), 7.52 (d, *J* = 8.8 Hz, 1H, ArH), 6.43 (dd, *J* = 8.8, 2.4 Hz, 1H, ArH), 6.32 (d, *J* = 2.1 Hz, 1H, ArH), 5.65 (s, 1H, HC=), 3.55 (td, *J* = 6.8, 3.3 Hz, 2H, CH_2), 3.36 (t, *J* = 6.6 Hz, 4H, CH_2), 2.92 (t, *J* = 6.7 Hz, 2H, CH_2), 2.10 – 1.96 (m, 4H, CH_2) ppm; ^{13}C NMR (125 MHz, $CDCl_3$) δ 181.3 (C=O), 165.4 (CF), 160.9 (C=), 150.4 (C=), 145.0 (CF), 138.6 (C=), 127.9 (=CH), 114.6 (C=), 110.4 (=CH), 110.2 (=CH), 105.1 (CF), 96.9 (C=), 91.0 (=CH), 47.5 (CH_2), 39.0 (CH_2), 28.6 (CH_2), 25.4 (CH_2) ppm; ^{19}F NMR (282 MHz, $CDCl_3$) δ -142.49 (dd, *J* = 23.7, 8.3 Hz, 2F, ArF), -155.93 (t, *J* = 20.8 Hz, 1F, ArF), -162.48 (dt, *J* = 23.9, 8.5 Hz, 2F, ArF) ppm; $[M+H]^+_{measured}$ = 409.1337, calcd. for $C_{21}H_{18}F_5N_2O$: 409.1334.

General procedure for the preparation of boroisoquinolines

Into a round bottom flask 1 mmol appropriate 1-methylidene-3,4-dihydroisoquinoline (**17**) was measured {(*Z*)-1-(6-methoxy-3,4-dihydroisoquinolin-1(2H)-ylidene)propan-2-one (**17a**) (0.22 g), (*Z*)-1-(6,7-dimethoxy-3,4-dihydroisoquinolin-1(2H)-ylidene)propan-2-one (**17b**) (0.25 g), (*Z*)-2-(6-methoxy-3,4-dihydroisoquinolin-1(2H)-ylidene)-1-phenylethan-1-one (**17c**) (0.28 g), (*Z*)-2-(6,7-dimethoxy-3,4-dihydroisoquinolin-1(2H)-ylidene)-1-phenylethan-1-one (**17d**) (0.31 g), (*Z*)-2-(6-methoxy-3,4-dihydroisoquinolin-1(2H)-ylidene)-1-(thiophen-2-yl)ethan-1-one (**17e**) (0.29 g), (*Z*)-2-(6,7-dimethoxy-3,4-dihydroisoquinolin-1(2H)-ylidene)-1-(thiophen-2-yl)ethan-1-one (**17f**) (0.32 g), (*Z*)-2-(6-methoxy-3,4-dihydroisoquinolin-1(2H)-ylidene)-1-(perfluorophenyl)ethan-1-one (**17g**) (0.32 g), (*Z*)-2-(6,7-dimethoxy-3,4-dihydroisoquinolin-1(2H)-ylidene)-1-(perfluorophenyl)ethan-1-one (**17h**) (0.40 g), (*Z*)-2-(6,7-

dimethoxy-3,4-dihydroisoquinolin-1(2H)-ylidene)-1-(4-nitrophenyl)ethan-1-one (**17j**) (0.35 g), (*Z*)-1-(6-(pyrrolidin-1-yl)-3,4-dihydroisoquinolin-1(2H)-ylidene)propan-2-one (**17k**) (0.26 g), (*Z*)-1-phenyl-2-(6-(pyrrolidin-1-yl)-3,4-dihydroisoquinolin-1(2H)-ylidene)ethan-1-one (**17l**) (0.32 g), (*Z*)-1-(perfluorophenyl)-2-(6-(pyrrolidin-1-yl)-3,4-dihydroisoquinolin-1(2H)-ylidene)ethan-1-one (**17m**) (0.41 g)}, and dissolved in 5 mL dichloromethane. The solution was cooled to 0 °C, 0.91 mL diisopropylethylamine (0.65 g, 5.0 mmol, 5.0 equiv) was added. 0.64 mL trifluorborane diethyl etherate (0.71 g, 5.0 mmol, 5.0 equiv) was added dropwise. The reaction mixture was stirred for 1h at room temperature. The reaction mixture was concentrated and purified by flash column chromatography on silica gel using hexane/dichloromethane.

(Z)-1-(2-(difluoroboranyl)-6-methoxy-3,4-dihydroisoquinolin-1(2H)-ylidene)propan-2-one (**18a**)

Yield: 0.20 g (77%), white crystals, m. p. 164–165 °C (EtOAc); ^{1}H NMR (500 MHz, $CDCl_3$) δ 7.64 (d, *J* = 8.8 Hz, 1H, ArH), 6.87 (dd, *J* = 8.7, 2.3 Hz, 1H, ArH), 6.78 (d, *J* = 1.9 Hz, 1H, ArH), 5.86 (s, 1H, HC=), 3.88 (s, 3H, OCH_3), 3.71 (t, *J* = 6.9 Hz, 2H, CH_2), 2.97 – 2.89 (m, 2H, CH_2), 2.18 (s, 3H, CH_3) ppm; ^{13}C NMR (125 MHz, $CDCl_3$) δ 174.7 (C=O), 163.8 (C=), 161.7 (C=), 140.2 (C=), 129.3 (=CH), 119.3 (C=), 113.3 (=CH), 92.4 (=CH), 55.6 (OCH_3), 40.2 (CH_2), 27.3 (CH_2), 23.1 (CH_3) ppm; ^{19}F NMR (282 MHz, $CDCl_3$) δ -139.33 (d, *J* = 17.1 Hz, 1F, BF), -139.45 (d, *J* = 17.0 Hz, 1F, BF) ppm; $[M+Na]^{+}_{measured}$ = 288.0990, calcd. for $C_{13}H_{14}BF_2NO_2Na$: 288.0983.

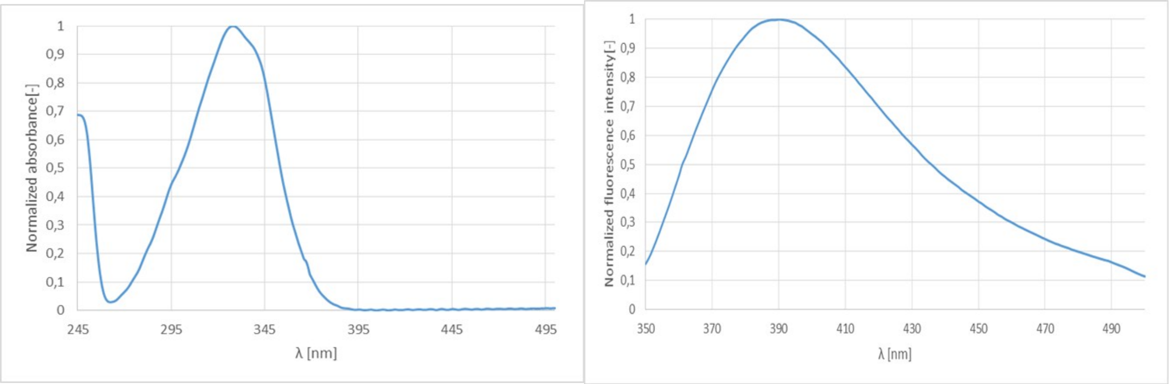

(Z)-1-(2-(difluoroboranyl)-6,7-dimethoxy-3,4-dihydroisoquinolin-1(2H)-ylidene)propan-2-one (**18b**)

Yield: 0.23 g (78%), white crystals, m. p. 228–230 °C (EtOAc); ^{1}H NMR (500 MHz, $CDCl_3$) δ 7.11 (s, 1H, ArH), 6.75 (s, 1H, ArH), 5.82 (s, 1H, HC=), 3.95 (s, 3H, OCH_3), 3.93 (s, 3H, OCH_3), 3.69 (t, *J* = 7.1 Hz, 2H, CH_2), 2.94 – 2.83 (m, 2H, CH_2), 2.19 (s, 3H, CH_3) ppm; ^{13}C

NMR (125 MHz, $CDCl_3$) δ 174.4 (C=O), 162.8 (C=), 161.1 (C=), 153.2 (C=), 147.8 (C=), 132.0 (C=), 118.3 (C=), 111.2 (=CH), 109.3 (=CH), 91.9 (=CH), 55.9 (OCH_3), 56.7 (OCH_3), 39.9 (CH_2), 26.1 (CH_2), 22.6 (CH_3) ppm; ^{19}F NMR (282 MHz, $CDCl_3$) δ -139.21 (d, J = 16.5 Hz, 1F, BF), -139.33 (d, J = 16.8 Hz, 1F, BF) ppm; $[M+Na]^+_{measured}$ = 318.1094, calcd. for $C_{14}H_{16}BF_2NO_3Na$: 318.1089.

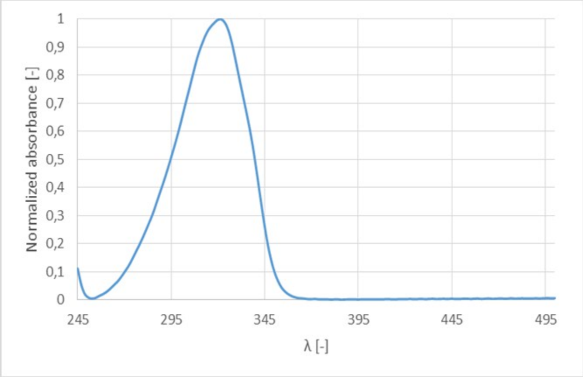

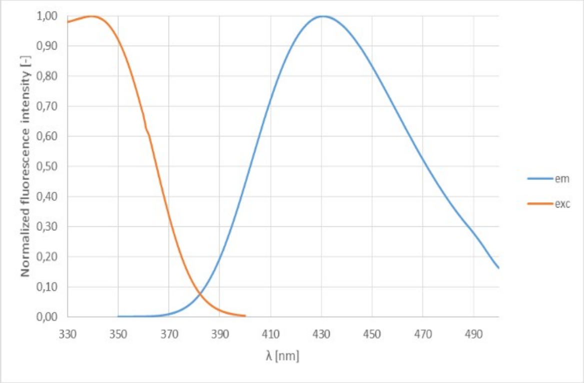

(Z)-2-(2-(difluoroboranyl)-6-methoxy-3,4-dihydroisoquinolin-1(2H)-ylidene)-1-phenylethan-1-one (**18c**)

Yield: 0.23 g (70%), yellow crystals, m. p. 183°C ($CDCl_3$); ^{1}H NMR (500 MHz, $CDCl_3$) δ 7.98 (d, J = 7.3 Hz, 2H, ArH), 7.79 (d, J = 8.7 Hz, 1H, ArH), 7.54 – 7.40 (m, J = 24.5, 7.2 Hz, 3H, ArH), 6.91 (dd, J = 8.7, 2.3 Hz, 1H, ArH), 6.81 (s, 1H, ArH), 6.55 (s, 1H, HC=), 3.89 (s, 3H, OCH_3), 3.85 – 3.73 (m, 2H, CH_2), 2.97 (t, J = 7.0 Hz, 2H, CH_2) ppm; ^{13}C NMR (125 MHz, $CDCl_3$) δ 169.3 (C=O), 163.8 (C=), 162.1 (C=), 140.2 (C=), 134.1 (C=), 131.8. (=CH), 129.4 (=CH), 128.6 (=CH), 127.1 (=CH), 119.8 (C=), 113.41 (CH_2), 113.39 (CH_2), 89.7 (=CH), 55.7 (OCH_3), 40.4 (CH_2), 27.3 (CH_2) ppm; ^{19}F NMR (282 MHz, $CDCl_3$) δ -139.70 (d, J = 16.6 Hz, 1F, BF), -139.82 (d, J = 16.6 Hz, 1F, BF) ppm ; $[M+H]^+_{measured}$ = 328.1322, calcd. for $C_{18}H_{17}BF_2NO_2$: 328.1320.

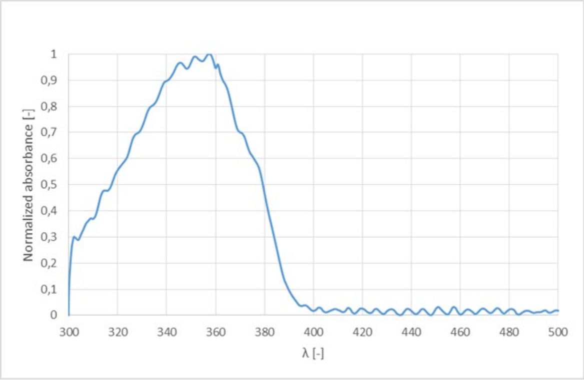

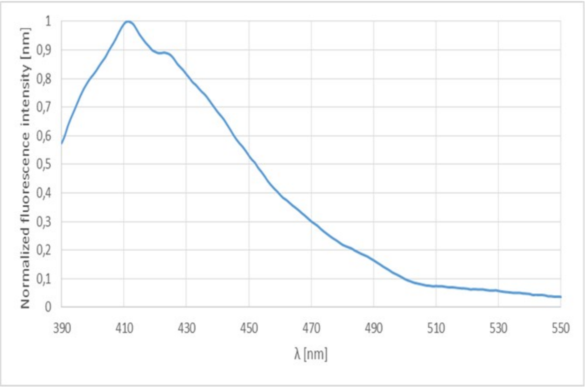

(Z)-2-(2-(difluoroboranyl)-6,7-dimethoxy-3,4-dihydroisoquinolin-1(2H)-ylidene)-1-phenylethan-1-one (**18d**)

Yield: 0.31 g (88%), yellow crystals, m. p. 219–221°C (EtOAc); ^{1}H NMR (500 MHz, $CDCl_3$) δ 8.00 – 7.91 (m, 2H, ArH), 7.51 – 7.39 (m, 3H, ArH), 7.25 (s, 1H, ArH), 6.77 (s, 1H, ArH), 6.49 (s, 1H, HC=), 3.98 (s, 3H, OCH_3), 3.97 (s, 3H, OCH_3), 3.75 (t, J = 7.1 Hz, 2H, CH_2), 2.93 – 2.89 (m, 2H, CH_2) ppm; ^{13}C NMR (125 MHz, $CDCl_3$) δ 169.5 (C=O), 162.0 (C=), 153.8 (C=), 148.4 (C=) 134.1 (C=), 132.4 (C=), 131.7 (=CH), 128.5 (=CH), 127.1 (=CH), 119.3 (C=), 110.7 (=CH), 110.0 (=CH), 87.8 (=CH), 56.5(OCH_3), 56.2 (OCH_3), 40.5 (CH_2), 26.6 (CH_2) ppm; ^{19}F NMR (282 MHz, $CDCl_3$) δ -139.46 (d, J = 16.0 Hz, 1F, BF), -139.57 (d, J = 16.1 Hz, 1F, BF) ppm; $[M+Na]^{+}_{measured}$ = 380.1248, calcd. for $C_{19}H_{18}BF_2NO_2Na$: 380.1246.

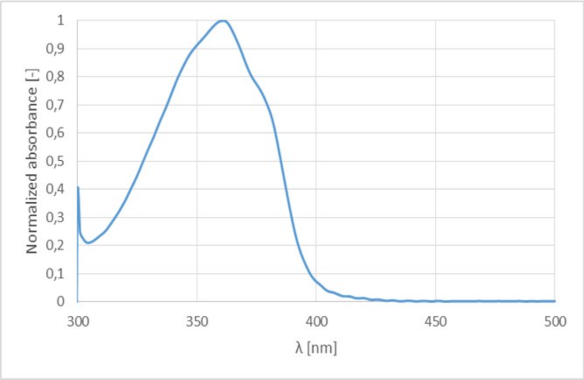

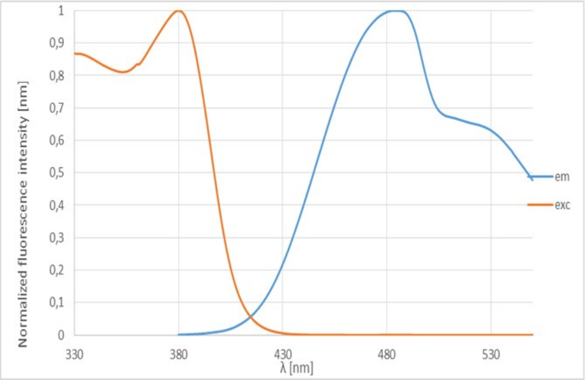

(Z)-2-(2-(difluoroboranyl)-6-methoxy-3,4-dihydroisoquinolin-1(2H)-ylidene)-1-(thiophen-2-yl)ethan-1-one (**18e**)

Yield: 0.17 g (50%), yellow crystals, m. p. 175−178°C ($CDCl_3$); ^{1}H NMR (500 MHz, $CDCl_3$) δ 7.79 – 7.77 (m, 1H, ArH), 7.75 (d, *J* = 8.8 Hz, 1H, ArH), 7.53 (dd, J = 4.9, 0.8 Hz, 1H, ArH), 7.15-7.11 (m, 1H, ArH), 6.94-6.90 (m, 1H, ArH), 6.81 (d, *J* = 2.3 Hz, 1H, ArH), 6.39 (s, 1H, HC=), 3.90 (s, 3H, OCH_3), 3.78 (t, *J* = 7.0 Hz, 2H, CH_2), 3.00 – 2.95 (m, 2H, CH_2) ppm; ^{13}C NMR (125 MHz, $CDCl_3$) δ 163.8 (C=O), 161.8 (C=), 140.1 (C=), 136.0 (C=), 130.7 (=CH), 129.6 (=CH), 129.3 (=CH), 128.2 (=CH), 114.1 (C=), 113.41 (=CH), 113.36 (=CH), 111.0 (C=), 89.1 (=CH), 55.6 (OCH_3), 40.4 (CH_2), 27.4 (CH_2) ppm; ^{19}F NMR (282 MHz, $CDCl_3$) δ -140.33 (d, *J* = 16.6 Hz, 1F, BF), -140.45 (d, *J* = 16.6 Hz, 1F, BF) ppm; $[M+Na]^+_{measured}$ = 356.0700, calcd. for $C_{16}H_{14}BF_2NO_2SNa$: 356.0704.

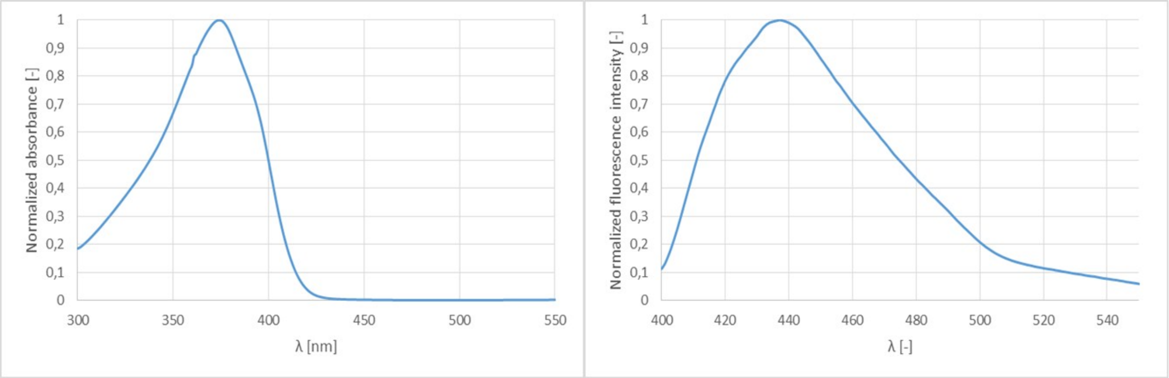

(Z)-2-(2-(difluoroboranyl)-6,7-dimethoxy-3,4-dihydroisoquinolin-1(2H)-ylidene)-1-(thiophen-2-yl)ethan-1-one (**18f**)

Yield: 0.17 g (46%), yellow crystals, m. p. 238–239 °C (EtOAc); ^{1}H NMR (500 MHz, DMSO-d_6) δ 8.20 (d, *J* = 3.8 Hz, 1H, ArH), 7.93 (d, *J* = 4.9 Hz, 1H, ArH), 7.59 (s, 1H, ArH), 7.31 – 7.25 (m, 1H, ArH), 7.07 (s, 1H, ArH), 6.95 (s, 1H, HC=), 3.92 (s, 3H, OCH_3), 3.92 (s, 3H, OCH_3), 3.88 (s, 3H, OCH_3), 3.61 (t, *J* = 7.1 Hz, 2H, CH_2), 2.91 (t, *J* = 7.1 Hz, 2H, CH_2) ppm; ^{13}C NMR (125 MHz, $CDCl_3$) δ 163.5 (C=O), 162.1 (C=), 154.2 (C=), 148.4 (C=), 138.8 (C=), 132.9 (C=), 132.8 (=CH), 131.0 (=CH), 129.2 (=CH), 118.8 (C=), 111.8 (=CH), 89.7 (=CH), 56.8 (OCH_3), 56.4 (OCH_3), 40.7 (CH_2) 26.2 (CH_2) ppm; ^{19}F NMR (282 MHz, DMSO-d_6) δ - -137.09 (d, *J* = 14.6 Hz, 1F, BF), -137.21 (d, *J* = 13.4 Hz, 1F, BF) ppm; $[M+Na]^+_{measured}$ = 386.0807, $C_{17}H_{16}BF_2NO_3SNa$: 386.081.

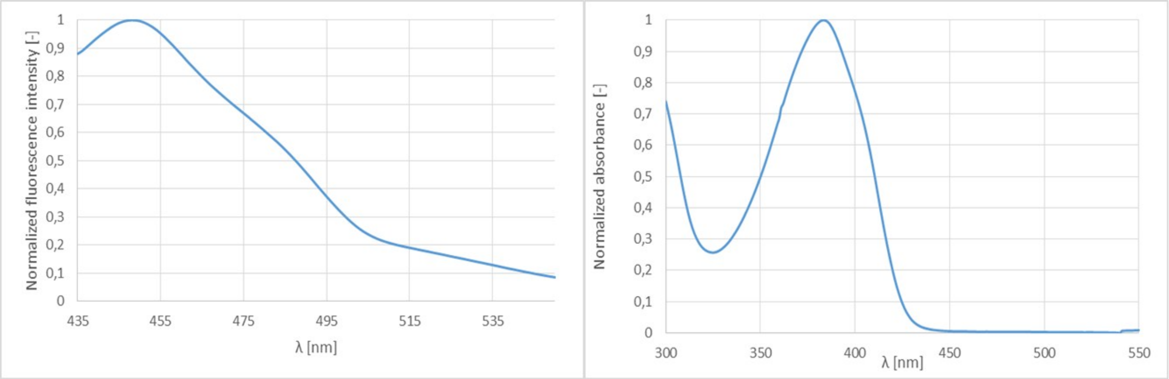

(Z)-2-(2-(difluoroboranyl)-6-methoxy-3,4-dihydroisoquinolin-1(2H)-ylidene)-1-(perfluorophenyl)ethan-1-one (**18g**)

Yield: 0,35 g (85%), white crystals, m. p. 144-145 °C (MeCN), ^{1}H NMR (500 MHz, $CDCl_3$) δ 7.69 (d, J = 8.8 Hz, 1H, ArH), 6.89 (dd, J = 8.8, 2.5 Hz, 1H, ArH), 6.81 (s, 1H, ArH), 6.32 (s, 1H, CH), 3.89 (s, 3H, OCH_3), 3.80 (t, J = 7.2 Hz, 2H, CH_2), 3.05–2.97 (m, 2H, CH_2) ppm; ^{13}C NMR (125 MHz, $CDCl_3$) δ 171.0 (C=O), 168.5 (=CF), 161.9 (C=), 158.6 (=CF), 157.2 (=CF), 154.5 (C=), 148.6 (C=), 132.8 (C=), 120.7 (=CH), 118.4 (C=), 110.8 (=CH), 109.9 (=CH), 97.6 (=CH), 56.3 (OCH_3) 40.9 (CH_2), 26.3 (CH_2) ppm; ^{19}F NMR (282 MHz, $CDCl_3$) δ -138.64 – -138.91 (m, 2F, ArF), -139.23 (d, J = 15.5 Hz, 1F, BF), -139.34 (d, J = 15.5 Hz, 1F, BF), -150.50 – -150.84 (m, J = 22.5, 19.4 Hz, 1F, ArF), -160.97 – -161.39 (m, 2F, ArF) ppm; HRMS $[M+NH_4]^{+}_{measured}$ = 435.1100, calcd. for $C_{18}H_{15}BF_7N_2O_2$: 435.1109.

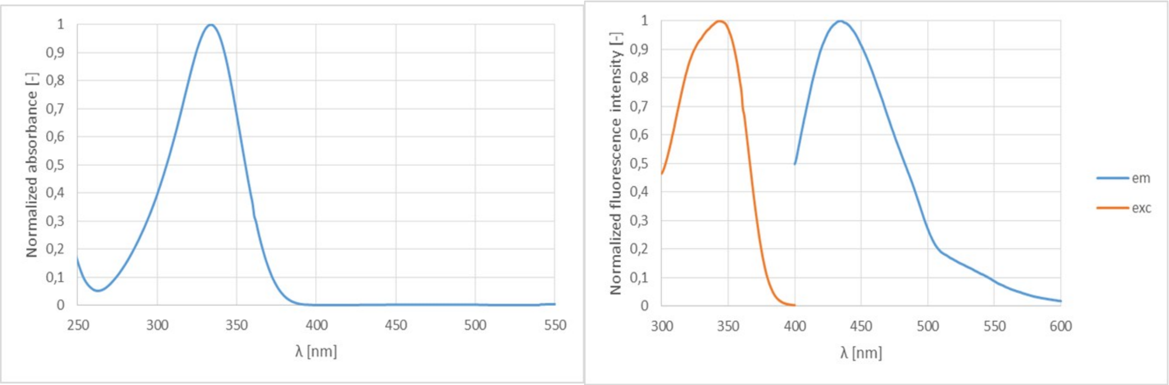

(Z)-2-(2-(difluoroboranyl)-6,7-dimethoxy-3,4-dihydroisoquinolin-1(2H)-ylidene)-1-(perfluorophenyl)ethan-1-one (**18h**)

Yield: 0.40 g (89%), yellow crystals, m. p. 217 °C (EtOAc); ^{1}H NMR (500 MHz, $CDCl_3$) δ 7.10 (s, 1H, ArH), 6.81 (s, 1H, ArH), 6.23 (s, 1H, HC=), 3.98 (s, 3H, CH_3), 3.92 (s, 3H, CH_3), 3.84 – 3.79 (m, 2H, CH_2), 3.01 – 2.95 (m, 2H, CH_2) ppm; ^{13}C NMR (125 MHz, $CDCl_3$) δ

161.9 (C=O), 158.6(C=), 154.5 (C=), 148.6 (C=), 146.0 (C=), 143.8 (=CF), 137.8 (d, J = 255.0 Hz, =CF), 132.8 (C=), 130.3 (=CF), 118.4 (C=), 110.8 (=CH), 109.8 (=CH), 97.6 (=CH), 56.34 (OCH_3), 56.28 (OCH_3), 40.9 (CH_2), 26.3 (CH_2) ppm; ^{19}F NMR (282 MHz, $CDCl_3$) δ -138.70 – -138.95 (m, 2F, ArF), -139.02 (d, J = 15.1 Hz, 1F, BF), -139.13 (d, J = 14.9 Hz, 1F, BF), -150.60 (t, J = 20.2 Hz, 1F, ArF), -160.97 – -161.35 (m, 2F, ArF). ppm; $[M+H]^+_{measured}$ = 470.0769, calcd. for $C_{19}H_{13}BF_7NO_3$: 470.0774.

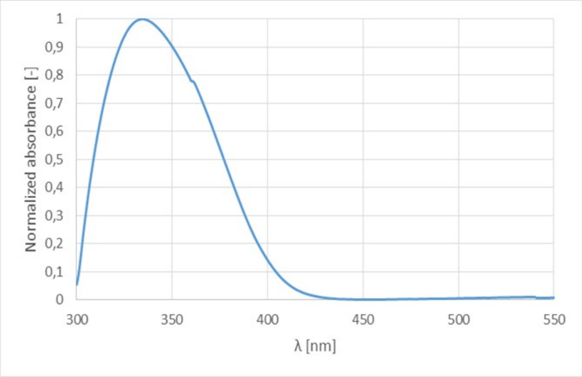

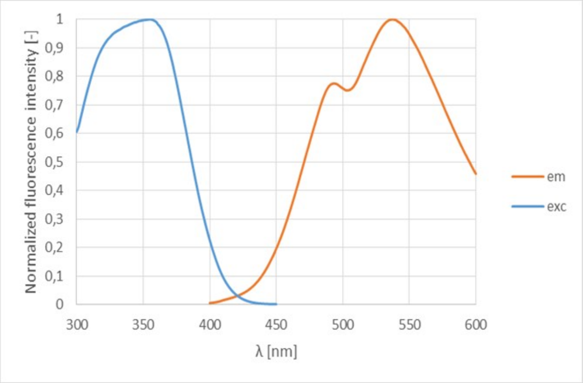

(Z)-2-(2-(difluoroboranyl)-6,7-dimethoxy-3,4-dihydroisoquinolin-1(2H)-ylidene)-1-(4-nitrophenyl)ethan-1-one (**18j**)

Yield: 0.19 g (49%), yellow crystals, m. p. 275-276 °C (MeCN); ^1H NMR (500 MHz, DMSO-d_6) δ 8.34 (s, 4H, ArH), 4.66 (s, 1H, ArH), 7.17 (s, 1H, ArH), 7.08 (s, 1H, HC=), 3.92 (s, 3H, OCH_3), 3.88 (s, 3H, OCH_3), 3.67 (t, J = 6.2 Hz, 2H, CH_2), 2.93 (t, J = 6.2 Hz, CH_2) ppm; ^{13}C NMR (125 MHz, $CDCl_3$) δ 169.9 (C=O), 164.8 (C=), 162.6 (C=), 154.6 (C=), 149.5 (C=), 148.5 (C=), 139.9 (C=), 133.3 (C=), 128.7 (=CH), 124.2 (=CH), 112.2 (=CH), 111.7 (=CH), 93.4 (=CH), 56.9 (OCH_3), 56.5 (OCH_3), 41.0 (CH_2), 26.0 (CH_2) ppm; ^{19}F NMR (282 MHz, DMSO-d_6) δ -136.26 – -136.84 (m, 2F, BF) ppm; $[M+H]^+_{measured}$ = 403.1271, calcd. for $C_{19}H_{18}BF_2N_2O_5$: 403.1271.

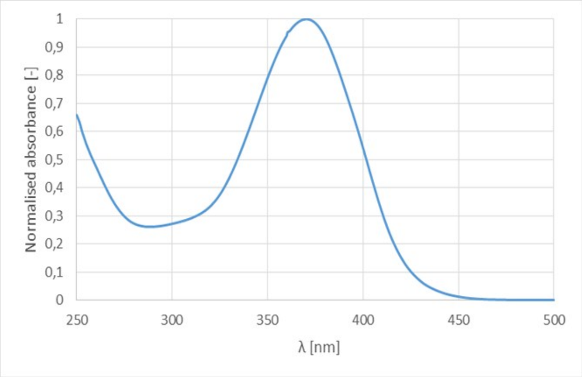

(Z)-1-(2-(difluoroboranyl)-6-(pyrrolidin-1-yl)-3,4-dihydroisoquinolin-1(2H)-ylidene)propan-2-one (**18k**)

Yield: 0.24 g (78%); yellow crystals; m. p. 196-198 °C ($CDCl_3$); ^{1}H NMR (500 MHz, $CDCl_3$): δ 7.52 (d, *J* = 8.8 Hz, 1H, ArH), 6.45 (dd, *J* = 8.8, 2.4 Hz, 1H, ArH), 6.33 (d, *J* = 1.9 Hz, 1H, ArH), 5.80 (s, 1H,), 3.67 (t, *J* = 6.9 Hz, 2H, CH_2), 3.38 (t, *J* = 6.6 Hz, 4H, CH_2), 2.91 – 2.83 (m, 2H, CH_2), 2.15 (s, 3H, CH_3), 2.07 – 2.03 (m, 4H, CH_2) ppm; ^{13}C NMR (125 MHz, $CDCl_3$) δ 172.5 (C=O), 161.5 (C=), 151.1 (C=), 139.9 (C=), 129.3 (=CH), 110.4 (=CH), 110.1 (=CH), 92.1 (=CH), 47.6 (CH_2), 40.4 (CH_2), 27.8 (CH_2), 25.4 (CH_2), 23.0 (CH_2) ppm; ^{19}F NMR (282 MHz, $CDCl_3$) δ -140.27 (d, *J* = 17.1 Hz, 1F, BF), -140.40 (d, *J* = 17.4 Hz, 1F, BF) ppm; $[M+H]^+_{measured}$ = 305.1640, calcd. for $C_{16}H_{20}BF_2N_2O$: 305.1631.

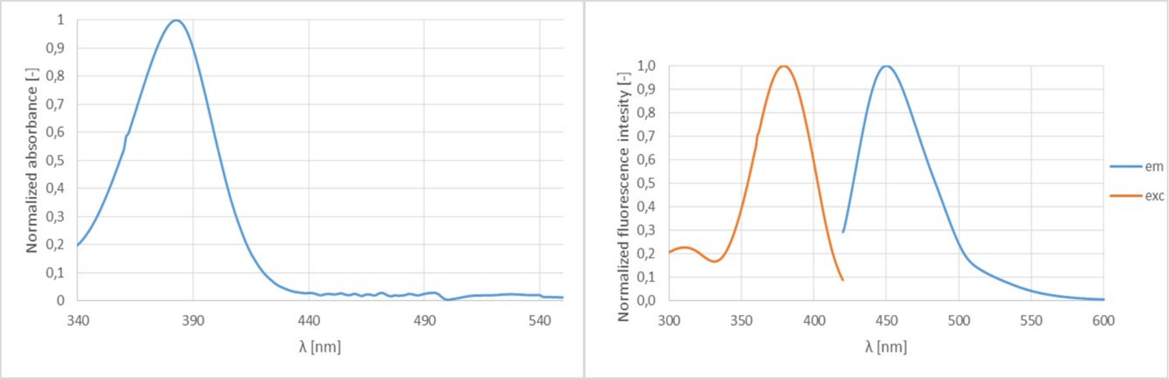

(Z)-2-(2-(difluoroboranyl)-6-(pyrrolidin-1-yl)-3,4-dihydroisoquinolin-1(2H)-ylidene)-1-phenylethan-1-one (**18l**)

Yield: 0.36 g (97%); orange crystals; m. p. 233-235 °C ($CDCl_3$); ^{1}H NMR (500 MHz, $CDCl_3$): δ 7.99 – 7.96 (m, 2H, ArH), 7.67 (d, *J* = 8.8 Hz, 1H, ArH), 7.46 – 7.41 (m, 2H, ArH), 6.51 – 6.47 (m, 2H, ArH), 6.36 (d, *J* = 2.1 Hz, 1H, HC=), 3.76 (t, *J* = 7.0 Hz, 2H, CH_2), 3.40 (t, *J* = 6.6 Hz, 4H, CH_2), 2.94 – 2.88 (m, 2H, CH_2), 2.10 – 2.00 (m, 4H, CH_2) ppm; ^{13}C NMR (125 MHz, $CDCl_3$) δ 173.5 (C=O), 167.4 (C=), 161.3 (C=), 150.7 (C=), 139.4 (C=), 130.7 (=CH), 128.8 (=CH), 127.9 (=CH), 126.4 (=CH), 109.9 (=CH), 109.7 (=CH), 109.5 (=C), 89.2 (=CH), 47.2 (CH_2), 40.0 (CH_2), 27.4 (CH_2), 24.9 (CH_2) ppm; ^{19}F NMR (282 MHz, $CDCl_3$) δ -140.65 (d, *J* = 16.8 Hz, 1F, BF), -140.77 (d, *J* = 17.2 Hz, 1F, BF) ppm; $[M+H]^+_{measured}$ = 367.1780, calcd. for $C_{21}H_{22}BF_2N_2O$: 367.1788.

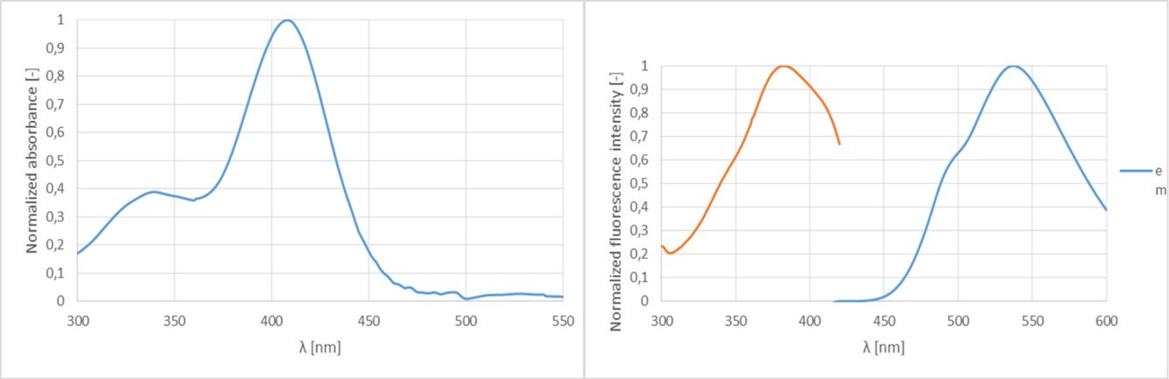

(Z)-2-(2-(difluoroboranyl)-6-(pyrrolidin-1-yl)-3,4-dihydroisoquinolin-1(2H)-ylidene)-1-(perfluorophenyl)ethan-1-one (**18m**)

Yield: 0.37 g (81%); yellow crystals; m. p. 214-216 °C ($CDCl_3$); ^{1}H NMR (500 MHz, $CDCl_3$): δ 7.52 (d, J = 8.9 Hz, 1H, ArH), 6.47 (dd, J = 8.9. 2.4 Hz, 1H, ArH), 6.37 (d, J = 2.0 Hz, 1H, ArH), 6.22 (s, 1H, HC=), 3.78 (t, J = 7.1 Hz, 2H, CH_2), 3.43 – 3.37 (m, 4H, CH_2), 2.98 – 2.89 (m, 2H, CH_2), 2.07 (t, J = 6.6 Hz, 4H, CH_2) ppm; ^{13}C NMR (125 MHz, $CDCl_3$) δ 181.0 (C=O), 169.7 (C=), 161.4 (C=), 160.2 (C=), 156.4 (=CF), 151.7 (C=), 140.0 (C=), 136.8 (=CF), 135.3 (=CF), 129.7 (=CH), 110.6 (=CH), 110.3 (=CH), 97.7 (=CH), 47.7 (CH_2), 40.8 (CH_2), 27.5 (CH_2), 25.4 (CH_2) ppm; ^{19}F NMR (282 MHz, $CDCl_3$) δ -138.97 (dd, J = 23.8, 6.5 Hz, 2F, ArF), -140.40 (d, J = 16.3 Hz, 1F, BF), -140.51 (d, J = 16.4 Hz, 1F, BF), -151.63 (t, J = 21.0 Hz, 1F, ArF), -161.59 (qd, J = 10.6, 4.4 Hz, 2F, ArF) ppm; $[M+NH_4]^+_{measured}$ = 474.1584, calcd. for $C_{21}H_{20}BF_7N_3O$: 474.1582.

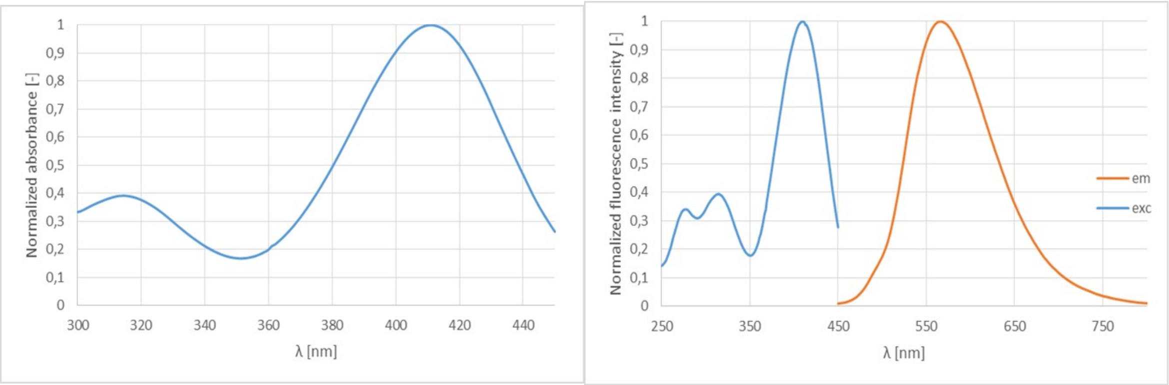

General procedure of the dehydrogenation

Into a microwave reactor tube 0.1 g boroisoquinoline (Z)-1-(2-(difluoroboranyl)-6-methoxy-3,4-dihydroisoquinolin-1(2H)-ylidene)propan-2-one (**18a**) (0.38 mmol), (Z)-1-(2-

(difluoroboranyl)-6,7-dimethoxy-3,4-dihydroisoquinolin-1(2H)-ylidene)propan-2-one (**18b**) (0.34 mmol), (*Z*)-2-(2-(difluoroboranyl)-6-methoxy-3,4-dihydroisoquinolin-1(2H)-ylidene)-1-phenylethan-1-one (**18c**) (0.31 mmol), (*Z*)-2-(2-(difluoroboranyl)-6,7-dimethoxy-3,4-dihydroisoquinolin-1(2H)-ylidene)-1-phenylethan-1-one (0.28 mmol) (**18d**), (*Z*)-2-(2-(difluoroboranyl)-6-methoxy-3,4-dihydroisoquinolin-1(2H)-ylidene)-1-(thiophen-2-yl)ethan-1-one (0.30 mmol) (**18e**), (*Z*)-2-(2-(difluoroboranyl)-6,7-dimethoxy-3,4-dihydroisoquinolin-1(2H)-ylidene)-1-(thiophen-2-yl)ethan-1-one (**18f**) (0.75 mmol)}was measured. 0.15 g Selcat Q6 10% Pd/C catalyst and 2 mL dioxane was added. Then the reaction mixture was heated 200 °C for 1.5h. The suspension was filtered through celite. The product was purified by flash column chromatography on silica gel using hexane/dichloromethane.

(Z)-1-(2-(difluoroboranyl)-6-methoxyisoquinolin-1(2H)-ylidene)propan-2-one (**19a**)

Yield: 0.072 g (73%), white crystals, m. p. 183-184 °C (hexane-EtOAc); ^{1}H NMR (500 MHz, DMSO-d_6) δ 8.47 (d, J = 9.3 Hz, 1H, ArH), 8.03 (d, J = 6.7 Hz, 1H, ArH), 7.66 (d, J = 6.8 Hz, 1H, ArH), 7.47 (d, J = 2.1 Hz, 1H, HC=), 7.39 (dd, J = 9.2, 2.3 Hz, 1H, HC=), 6.81 (s, 1H, HC=), 3.97 (s, 3H, OCH_3), 2.20 (s, 3H, CH_3) ppm; ^{13}C NMR (125 MHz, DMSO-d_6) δ 169.2 (C=O), 163.8 (C=), 151.4 (C=), 139.4 (C=), 132.0 (=CH), 128.9 (=CH), 121.2 (=CH), 118.4 (=CH), 117.4 (C=), 107.1 (=CH), 92.2 (=CH), 56.5 (OCH_3), 23.0 (CH_3) ppm; ^{19}F NMR (282 MHz, DMSO-d_6) δ -135.38 (d, J = 16.6 Hz, 1F, BF), -135.50 (d, J = 16.5 Hz, 1F, BF) ppm; $[M+Na]^+_{measured}$ = 286.0826, calcd. for $C_{13}H_{12}BF_2NO_2Na$: 286.0827.

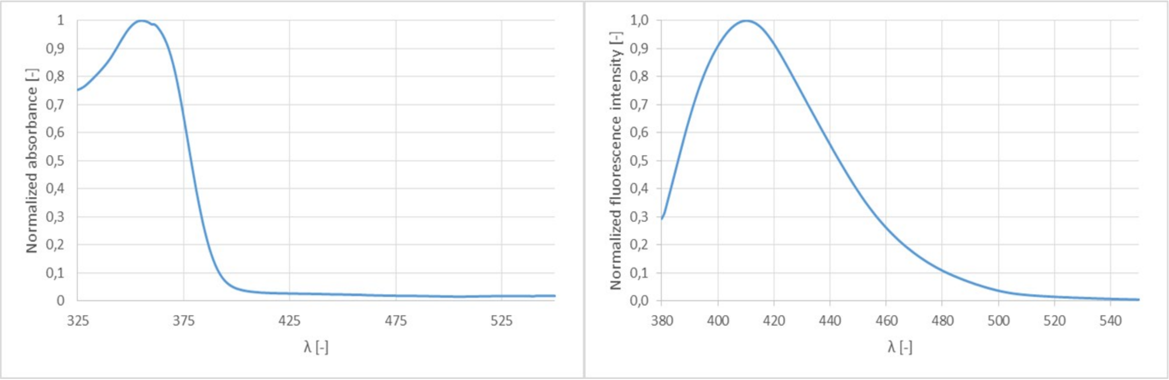

(Z)-1-(2-(difluoroboranyl)-6,7-dimethoxyisoquinolin-1(2H)-ylidene)propan-2-one (**19b**)

Yield: 0.070 g (70%), white crystals, m. p. 201–202 °C (EtOAc); ^{1}H NMR (500 MHz, DMSO-d_6) δ 7.96 (t, J = 12.1 Hz, 1H, ArH), 7.72 (s, 1H, ArH), 7.62 (d, J= 6.6 Hz, 1H, HC=), 7.47 (s, 1H, HC=), 6.83 (s, 1H, HC=), 3.98 (s, 3H, OCH_3), 3.97 (s, 3H, OCH_3), 2.19 (s, 3H,

CH_3) ppm; ^{13}C NMR (125 MHz, DMSO-d_6) δ 167.9 (C=O), 155.6 (C=), 151.5 (C=), 149.6 (C=), 134.0 (C=), 130.2 (=CH), 118.02 (C=), 117.99 (=CH), 106.9 (=CH), 105.4 (=CH), 92.4 (=CH), 56.7 (OCH_3), 56.6 (OCH_3), 22.8 (CH_3) ppm; ^{19}F NMR (282 MHz, DMSO-d_6) δ -135.87 (d, J = 16.1 Hz, 1F, BF), -135.99 (d, J = 16.3 Hz, 1F, BF) ppm; $[M+H]^+_{measured}$ = 294.1113, calcd. for $C_{14}H_{15}BF_2NO_2$: 294.1113.

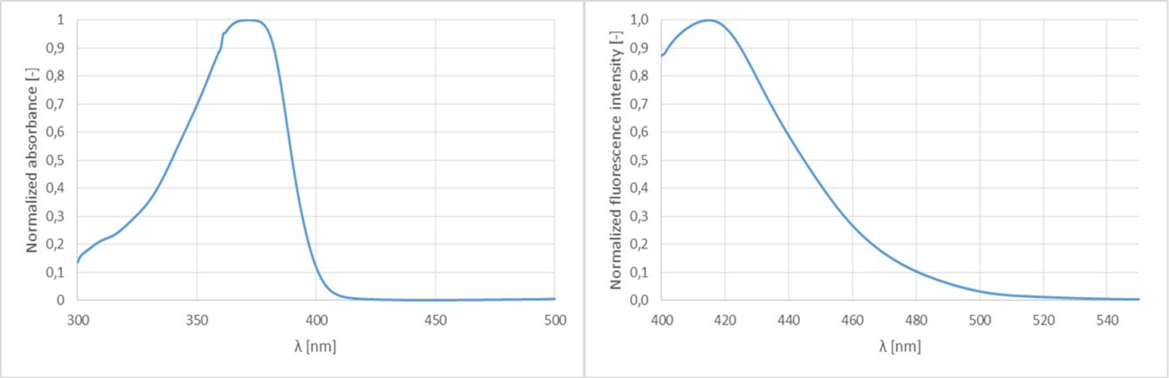

(Z)-2-(2-(difluoroboranyl)-6-methoxyisoquinolin-1(2H)-ylidene)-1-phenylethan-1-one (**19c**)
Yield: 0.077 g (77%), yellow crystals, m. p. 228-234 °C (EtOAc); ^1H NMR (500 MHz, DMSO-d_6) δ 8.90 (d, J = 9.3 Hz, 1H, ArH), 8.22 – 8.17 (m, 2H, ArH), 8.16 (d, J = 6.7 Hz, 1H, ArH), 7.76 (d, J = 6.8 Hz, 1H, ArH), 7.60 (s, 1H, ArH), 7.54 (dd, J = 13.4, 4.3 Hz, 4H, 2xArH, 2xHC=), 7.46 (dd, J = 9.3. 2.1 Hz, 1H, HC=), 3.99 (s, 3H, OCH_3) ppm; ^{13}C NMR (125 MHz, DMSO-d_6) δ 163.9 (C=O), 163.5 (C=), 151.5 (C=), 139.6 (C=), 134.3 (C=), 132.1 (=CH), 131.8 (=CH), 129.5 (=CH), 129.2 (=CH), 127.1 (=CH), 121.4 (=CH), 119.1 (=CH), 118.5 (C=), 107.0 (=CH), 90.5 (=CH), 56.5 (OCH_3) ppm; ^{19}F NMR (282 MHz, DMSO-d_6) δ 136.77 (d, J = 15.3 Hz, 1F, BF), -136.88 (d, J = 14.5 Hz, 1F, BF) ppm; $[M+Na]^+_{measured}$ = 348.0990, $C_{18}H_{14}BF_2NO_2Na$: 348.0983.

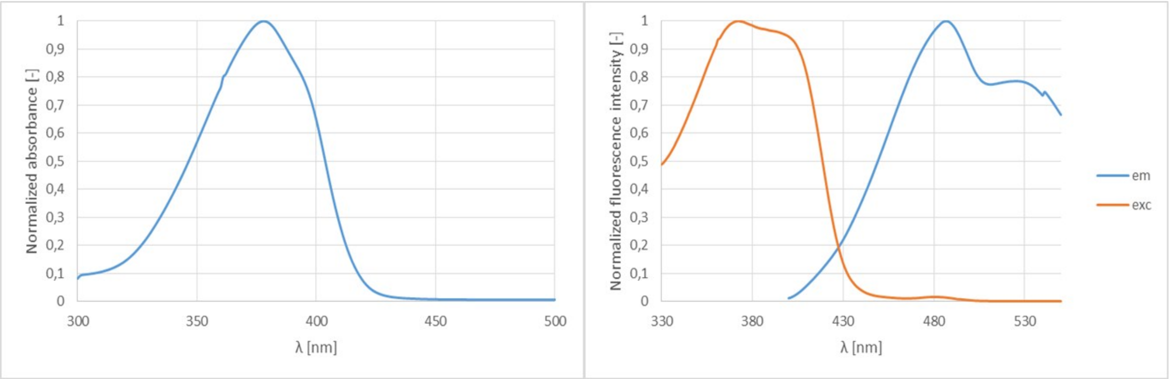

(Z)-2-(2-(difluoroboranyl)-6,7-dimethoxyisoquinolin-1(2H)-ylidene)-1-phenylethan-1-one **(19d)**

Yield: 0. 87 g (87%), yellow crystals, m. p. 240–245 °C (EtOAc); ^{1}H NMR (500 MHz, DMSO-d_6) δ 8.18 (dd, *J* = 6.4. 2.7 Hz, 2H, ArH), 8.10 (d, *J* = 6.5 Hz, 1H, ArH), 8.00 (s, 1H, ArH), 7.74 (d, *J* = 6.7 Hz, 1H, HC=), 7.57 - 7.52 (m, 5H, 3xArH, 2xHC=), 4.08 (s, 3H, OCH_3), 3.99 (s, 3H, OCH_3) ppm; ^{13}C NMR (125 MHz, DMSO-d_6) δ 162.5 (C=O), 155.7 (C=), 151.8 (C=), 149.4 (C=), 138.4 (C=), 134.3 (C=), 131.6 (=CH), 130.4 (=CH), 129.1 (=CH), 127.1 (=CH), 119.2 (C=), 118.8 (=CH), 107.0 (=CH), 105.8 (=CH), 90.8 (=CH), 57.1 (OCH_3), 56.7 (OCH_3) ppm; ^{19}F NMR (282 MHz, DMSO-d_6) δ -137.46 (d, *J* = 14.9 Hz, 1F, BF), -137.57 (d, *J* = 14.8 Hz, 1F, BF) ppm; $[M+Na]^{+}_{measured}$ = 378.1090, calcd. for $C_{19}H_{16}BF_2NO_3Na$: 378.1089.

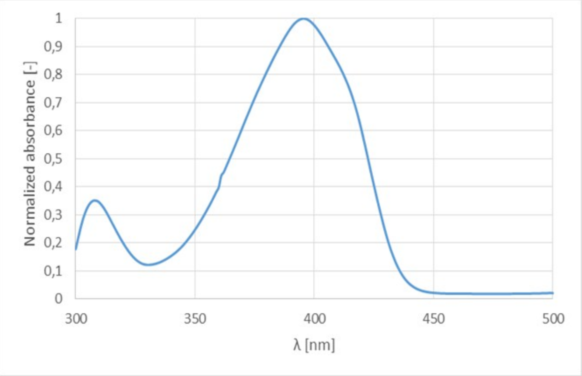

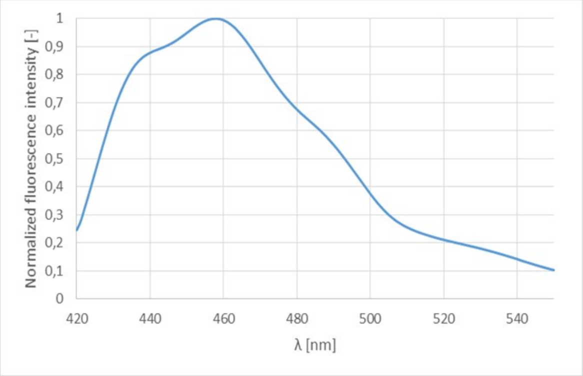

(Z)-2-(2-(difluoroboranyl)-6-methoxyisoquinolin-1(2H)-ylidene)-1-(thiophen-2-yl)ethan-1-one **(19e)**

After 1.5h, further 0.15 g Selcat Q6 was added, and the reaction mixture was heated 200 °C for 1.5h in MW reactor.

Yield: 0.052 g (52%), yellow crystals, m. p. 226–228°C (EtOAc); ^{1}H NMR (500 MHz, DMSO-d_6) δ 8.78 (t, *J* = 17.3 Hz, 1H, ArH), 8.18 (d, *J* = 3.7 Hz, 1H, ArH), 8.09 (d, *J* = 6.7 Hz, 1H, ArH), 7.87 (d, *J* = 4.9 Hz, 1H, ArH), 7.70 (d, *J* = 6.6 Hz, 1H, HC=), 7.50 (s, 2H, ArH, HC=), 7.44 (d, *J* = 9.3 Hz, 1H, HC=), 7.30 – 7.23 (m, 1H, HC=), 3.97 (s, 3H, OCH_3) ppm; ^{13}C NMR (125 MHz, DMSO-d_6) δ 163.9 (C=O), 159.2 (C=), 151.4 (C=), 139.5 (C=), 132.2 (=CH), 131.8 (=CH), 129.8 (=CH), 129.4 (=CH), 129.4 (=CH), 128.8 (C=), 121.3 (=CH), 118.6 (=CH), 118.3 (C=), 107.1 (=CH), 89.3 (=CH), 56.5 (OCH_3) ppm; ^{19}F NMR

(282 MHz, DMSO-d_6) δ -137.21 (d, J = 16.0 Hz, 1F, BF), -137.33 (d, J = 14.9 Hz, 1F, BF) ppm; $[M+H]^+_{measured}$ = 332.0724, calcd. for $C_{16}H_{13}BF_2NO_2S$: 332.0728.

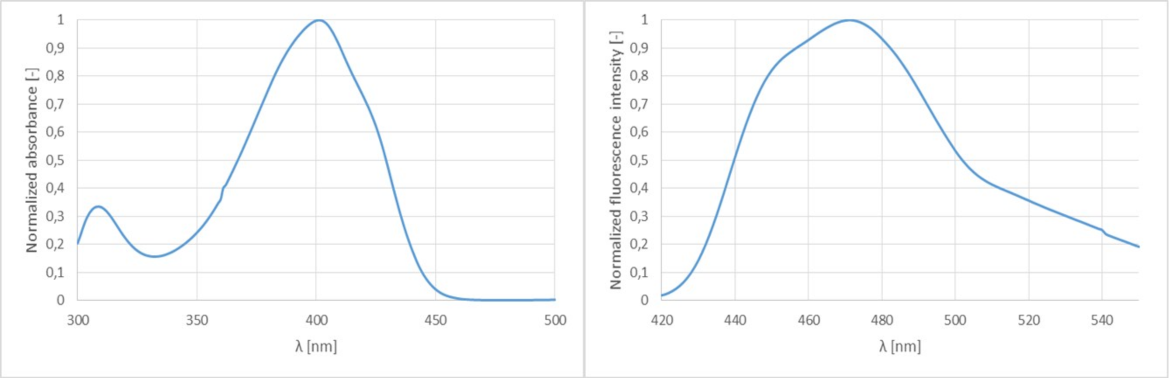

(Z)-2-(2-(difluoroboranyl)-6,7-dimethoxyisoquinolin-1(2H)-ylidene)-1-(thiophen-2-yl)ethan-1-one (**19f**)

Yield: 0.048 g (48%), yellow crystals, m. p. 270–272 °C (DMSO-d_6); ^{1}H NMR (500 MHz, DMSO-d_6) δ 8.20 (d, J = 3.6 Hz, 1H, ArH), 8.05 (d, J = 6.4 Hz, 1H, ArH), 7.92 (s, 1H, ArH), 7.85 (d, J = 4.8 Hz, 1H, HC=), 7.68 (d, J = 6.6 Hz, 1H, HC=), 7.52 (s, 1H, ArH), 7.42 (s, 1H, ArH), 7.27 (t, J = 4.1 Hz, 1H, HC=), 4.06 (s, 3H, OCH_3), 3.98 (s, 3H, OCH_3) ppm; ^{13}C NMR (125 MHz, DMSO-d_6) δ 158.3 (C=O), 155.7 (C=), 151.8 (C=), 149.3 (C=), 139.5 (C=), 134.2 (C=), 131.3 (=CH), 130.5 (=CH), 129.5 (=CH), 129.1 (=CH), 119.0 (C=), 118.4 (=CH), 107.0 (=CH), 105.7 (=CH), 89.6 (=CH), 57.0 (OCH_3), 56.7 (OCH_3) ppm; ^{19}F NMR (282 MHz, DMSO-d_6) δ -137.84 (d, J = 14.7 Hz, 1F, BF), -137.96 (d, J = 14.7 Hz, 1F, BF) ppm; $[M+H]^+_{measured}$ = 362.0829, calcd. for $C_{17}H_{15}BF_2NO_3S$: 362.0834.

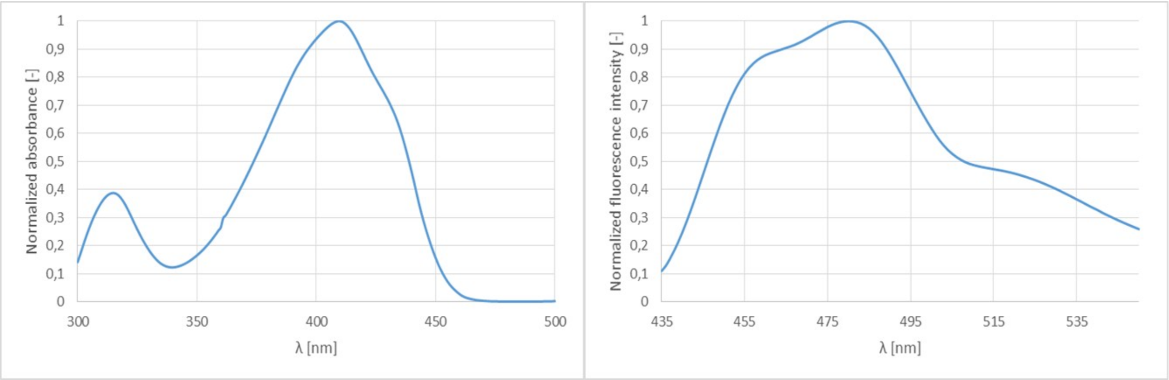

(Z)-1-(2-(diphenylboranyl)-6,7-dimethoxy-3,4-dihydroisoquinolin-1(2H)-ylidene)propan-2-one (**20b**)

In a sealed tube 0.25 g (*Z*)-1-(6-methoxy-3,4-dihydroisoquinolin-1(2H)-ylidene)propan-2-one (**17a**) (1.0 mmol) was dissolved in 3 mL anhydrous toluene. The tube was flushed with argon, then heated 90 °C for 1h. The reaction mixture was concentrated under reduced pressure. The residue was purified by flash column chromatography on silica gel using hexane/dichloromethane.

Yield: 0.35 g (84%), yellow crystals, m. p. 207-208 °C (hexane); ^{1}H NMR (500 MHz, $CDCl_3$) δ 7.48 – 7.45 (m, 4H, ArH), 7.29 (t, J = 7.2 Hz, 4H, ArH), 7.25 – 7.21 (m, 2H, ArH), 7.15 (s, 1H, ArH), 6.70 (s, 1H, ArH), 5.67 (s, 1H, HC=), 3.94 (s, 6H, OCH_3), 3.34 – 3.29 (m, 2H, CH_2), 2.84 – 2.79 (m, 2H, CH_2), 2.14 (s, 3H, CH_3) ppm; ^{13}C NMR (125 MHz, $CDCl_3$) δ 176.9 (C=O), 159.9 (C=), 148.0 (C=), 133.0 (=CH), 131.8 (C=), 127.1 (=CH), 126.2 (=CH), 120.1 (C=), 110.4 (=CH), 109.7 (=CH), 92.9 (=CH), 56.3 (OCH_3), 56.1 (OCH_3), 44.4 (CH_2), 26.9 (CH_2), 23.7 (CH_3) ppm; $[M+H]^{+}_{measured}$ = 412.2078, calcd. for $C_{26}H_{27}BNO_3$: 412.2079.

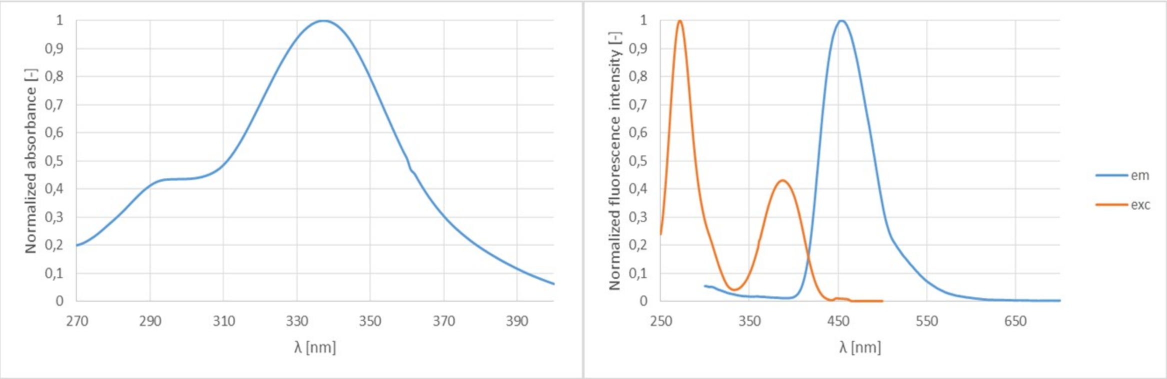

(Z)-1-(6,7-dimethoxy-3,4-dihydroisoquinolin-1(2H)-ylidene)propan-2-one tris(perfluorophenyl)borane salt (**22b**)

In a sealed tube 0.25 g (*Z*)-1-(6-methoxy-3,4-dihydroisoquinolin-1(2H)-ylidene)propan-2-one (**17a**) (1.0 mmol) was dissolved in 3 mL anhydrous toluene. The tube was flushed with argon, then heated 90 °C for 1h. The reaction mixture was concentrated under reduced pressure. The residue was purified by flash column chromatography on silica gel using hexane/dichloromethane.

Yield: 0.31 g (41%), yellow crystals, m. p. 100-101°C (MeCN); ^{1}H NMR (500 MHz, acetone-d_6) δ 10.71 (s, 1H, NH), 7.53 (s, 1H, ArH), 7.08 (s, 1H, ArH), 6.18 (s, 1H, HC=), 3.96 (s, 3H, OCH_3), 3.89 (s, 3H, OCH_3), 3.69 (td, J = 7.4, 3.7 Hz, 2H, CH_2), 3.07 (t, J = 7.3 Hz, 2H, CH_2), 2.02 (s, 3H, CH_3) ppm; ^{13}C NMR (125 MHz, acetone-d_6) δ 177.9 (C=O), 163.7 (C=), 155.1 (C=), 152.8 (=CF), 148.8 (C=), 143.8 (=CF), 138.0 (=CF), 133.2 (C=), 118.0 (C=), 111.5

(=CH), 110.8 (=CH), 94.2 (=CH), 55.7 (OCH_3), 55.6 (OCH_3), 39.5 (CH_2), 25.9 (CH_2), 23.0 (CH_3) ppm; ^{19}F NMR (282 MHz, $CDCl_3$) δ -134.79 – -135.11 (m, 3F, ArF), -136.07 – -136.41 (m, 3F, ArF), -158.33 (t, *J* = 20.3 Hz, 3F, ArF), -164.27 – -164.56 (m, 6F, ArF). ppm; In the HRMS the isoquinolinium cation could be detected: $[M+H]^+_{measured}$ = 248.1291, calcd. for $C_{14}H_{18}NO_3$: 248.1281. The LRMS spectrum of the salt is presented in the Supplementary Material showing the full molecular mass in negative ionization.

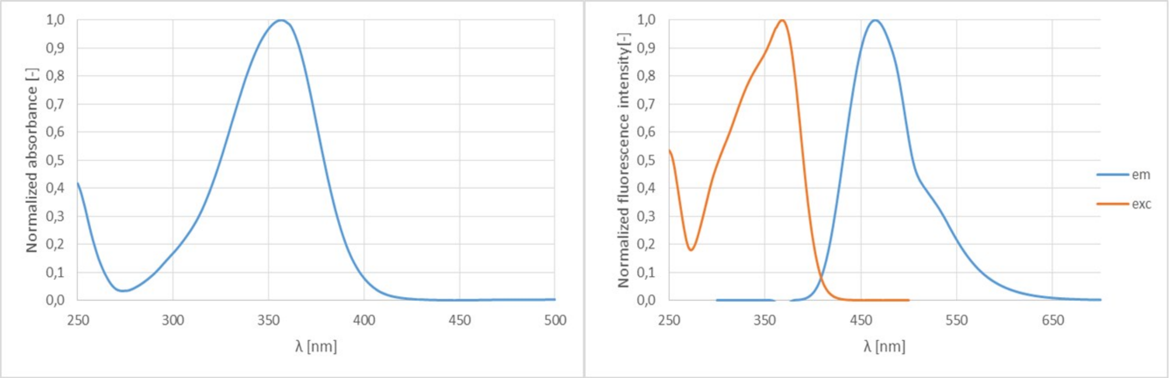

(Z)-1-(2-(bis(perfluorophenyl)boranyl)-6,7-dimethoxy-3,4-dihydroisoquinolin-1(2H)-ylidene)propan-2-one (**21b**)

In a sealed tube 0.76 g (*Z*)-1-(6,7-dimethoxy-3,4-dihydroisoquinolin-1(2H)-ylidene)propan-2-one tris(perfluorophenyl)borane salt (**22b**) (1.0 mmol) was dissolved in 3 mL anhydrous toluene. The tube was flushed with argon, then heated 200 °C for 16h. The reaction mixture was concentrated under reduced pressure. The residue was purified by preparative thin lyar chromatography on silica gel using dichloromethane.

Yield: 0.018g (3%), yellow crystals, m. p. 171-173 °C (MeCN); ^{1}H NMR (500 MHz, $CDCl_3$) δ 7.15 (s, 1H, ArH), 6.71 (s, 1H, ArH), 5.80 (s, 1H, HC=), 3.95 (s, 6H, 2xOCH_3), 3.37 (t, *J* = 7.1 Hz, 2H, CH_2), 2.88 (t, *J* = 7.2 Hz, 2H, CH_2), 2.12 (s, 3H, OCH_3) ppm; ^{13}C NMR (125 MHz, $CDCl_3$) δ 176.3 (C=O), 161.1 (C=), 153.9 (C=), 149.4 (=CF), 148.2 (C=), 147.5 (=CF), 143.7 (=CF), 132.2 (C=), 119.1 (C=), 110.4 (=CH), 109.9 (=CH), 93.2 (=CH), 56.3 (OCH_3), 56.2 (OCH_3), 43.5 (CH_2), 26.3 (CH_2), 23.1 (CH_3) ppm; ^{19}F NMR (282 MHz, $CDCl_3$) δ -135.51 (dd, *J* = 24.1, 9.2 Hz, 4F, ArF), -157.38 (t, *J* = 20.3 Hz, 2F, ArF), -164.08 (td, *J* = 23.9, 9.2 Hz, 4F, ArF) ppm; $[M+H]^+_{measured}$ = 592.1136, calcd. for $C_{26}H_{17}BF_{10}NO_3$: 592.1142.

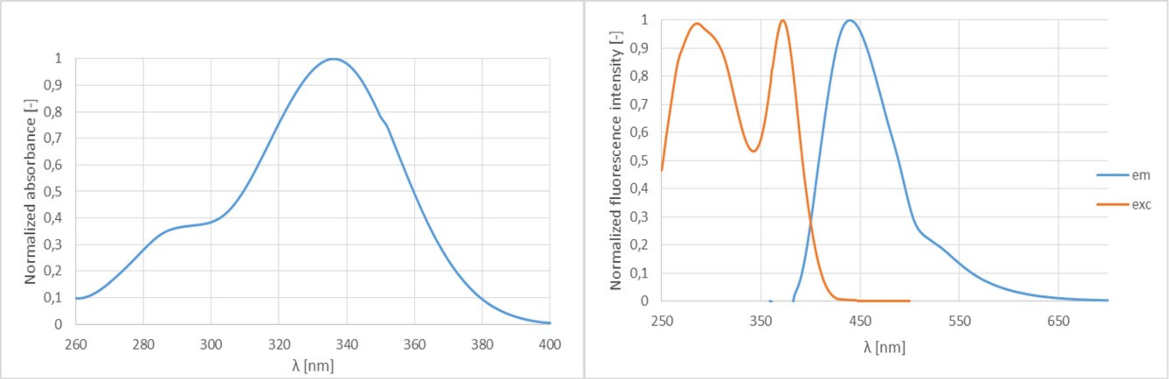

(Z)-2-(2-(bis(perfluorophenyl)boranyl)-6-(pyrrolidin-1-yl)-3,4-dihydroisoquinolin-1(2H)-ylidene)-1-phenylethan-1-one (**21l**)

In a sealed tube 0.32 g (*Z*)-1-phenyl-2-(6-(pyrrolidin-1-yl)-3,4-dihydroisoquinolin-1(2H)-ylidene)ethan-1-one (**17l**) (1.0 mmol) was dissolved in 3 mL anhydrous toluene. The tube was flushed with argon, then heated 200 °C for 9h. The reaction mixture was concentrated under reduced pressure. The residue was purified by flash column chromatography on silica gel using hexane/dichloromethane.

Yield: 0.013 g (2%), yellow crystals, m. p. 280-281 °C (MeCN); ^{1}H NMR (500 MHz, $CDCl_3$) δ 7.88 (d, J = 7.8 Hz, 2H, ArH), 7.73 (d, J = 8.9 Hz, 1H, ArH), 7.48 – 7.38 (m, 3H, ArH), 6.54 – 6.47 (m, 2H, ArH), 6.33 (s, 1H, HC=), 3.40 (q, J = 6.6 Hz, 4H, CH_2), 2.93 (t, J = 7.0 Hz, 2H, CH_2), 2.07 (t, J = 6.4 Hz, 2H, CH_2), 1.31 – 1.21 (m, 4H, CH_2) ppm; ^{13}C NMR (125 MHz, $CDCl_3$) δ 172.5 (C=O), 151.3 (C=), 147.5 (=CF), 144.5 (=CF), 143.9 (=CF) 140.5 (C=), 139.5 (C=), 139.0 (=CF), 134.6 (C=), 132.2 (C=), 131.3 (=CH), 129.5 (=CH), 128.5 (=CH), 127.0 (=CH), 110.4 (=CH), 110.1 (=CH), 90.2 (=CH), 47.8 (CH_2), 43.6 (CH_2), 29.7 (CH_2), 25.4 (CH_2) ppm; ^{19}F NMR (282 MHz, $CDCl_3$) δ -135.61 (dd, *J* = 24.0, 8.1 Hz, 4F, ArF), -158.02 (t, *J* = 20.3 Hz, 2F, ArF), -164.27 – -164.58 (m, 4F, ArF). ppm; $[M+H]^{+}_{measured}$ = 663.1657, calcd. for $C_{33}H_{22}BF_{10}N_2O$: 663.1665.

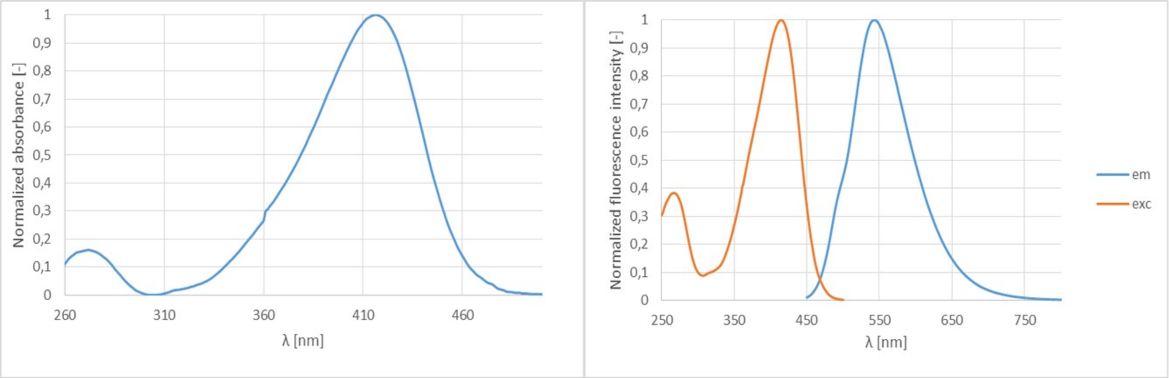

Procedure for synthesis of acetyl cysteine adducts

0.01 g (*Z*)-2-(2-(difluoroboranyl)-6,7-dimethoxy-3,4-dihydroisoquinolin-1(2H)-ylidene)-1-(perfluorophenyl)ethan-1-one (**18h**) (0.022 mmol) was dissolved in 1 mL DMSO. 0.0015 g K_2CO_3 was added, and the reaction mixture was heated 36 °C for 2 days. The product was purified by preparative HPLC using MeCN/water.

(Z)-N-acetyl-S-(4-(2-(2-(difluoroboranyl)-6,7-dimethoxyisoquinolin-1(2H)-ylidene)acetyl)-2,3,5,6-tetrafluorophenyl)cysteine (**24**)

Yield: 0.013 g (81%); yellow crystals; m. p. 189-199 °C (MeCN); ^{1}H NMR (500 MHz, $CDCl_3$): δ 7.365 (s, 1H, COOH), 7.355 (s, 1H, ArH), 7.13 (s, 1H, ArH), 6.80 (s, 1H, HC=), 6.30 (s, 1H, HC=), 4.70 (s, 1H, NH), 3.97 (s, 3H, OCH_3), 3.93 (s, 3H, OCH_3), 3.83-3.77 (m, 2H, CH_2), 3.52 (d, J = 4.4 Hz, 2H, CH_2), 3.00-2.91 (m, 2H, CH_2), 2.02 (s, 3H, CH_3) ppm; ^{13}C NMR (125 MHz, $CDCl_3$) δ 171.7 (C=O), 170.4 (COOH), 161.5 (C=O), 158.5 (C=), 154.0 (C=), 148.1 (C=), 147.8 (CF), 154.9 (CF), 144.7 (CF), 142.8 (CF), 140.3 (C=), 132.3 (C=), 128.5 (=CH), 126.8 (d, J=82.4 Hz, =CH), 117.9 (C=), 115.7 (C=), 109.8 (d, J=105.4 Hz, =CH), 97.3 (=CH), 55.8 (OCH_3), 51.8 (OCH_3), 40.4 (CH_2), 29.2 (CH_2), 25.8 (CH_2), 22.4 (CH_3) ppm; ^{19}F NMR (282 MHz, $CDCl_3$) δ -132.45 (dd, J = 23.7, 12.0 Hz, 2F, ArF), -138.22 – -138.60 (m, 2F, BF), -138.61 – -138.80 (m), -139.27 (dd, J = 23.3, 11.5 Hz, 2F, ArF) ppm; $[M+NH_4]^+_{measured}$ = 608.1451, calcd. for $C_{24}H_{25}BF_6N_3O_6S$: 608.1456.

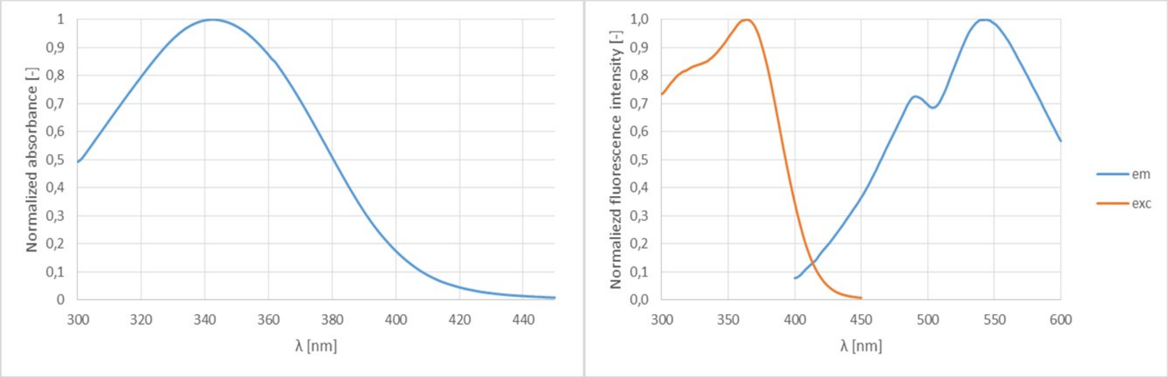

2. Optical spetroscopy data for boroisoquinolines

Entry		R^{1*}	R^2	R^3	BX	λ_{abs}^{max} [nm]	λ_{exc}^{max} [nm]**	λ_{em}^{max} [nm]	Δλ [nm]	FWMH [nm]
1	**18a**	MeO	H	Me	BF_2	328		390	62	53
2	**18b**	MeO	MeO	Me	BF2	321	339	431	110	45
3	**18k**	Py	H	Me	BF_2	383	379	450	67	40
4	**18c**	MeO	H	Ph	BF_2	357		411	54	60
5	**18d**	MeO	MeO	Ph	BF_2	361	380	483	122	57
6	**18l**	Py	H	Ph	BF_2	408	406	537	129	51
7	**21l**	Py	H	Ph	$B(C_6F_5)_2$	417	415	544	127	62

8	**18e**	MeO	H	2- thienyl	BF_2	374		437	63	55
9	**18f**	MeO	MeO	2- thienyl	BF_2	383		448	65	53
10	**18g**	MeO	H	C_6F_5	BF_2	334	343	435	101	48
11	**18h**	MeO	MeO	C_6F_5	BF_2	334	356	537	203	66
12	**18m**	Py	H	C_6F_5	BF_2	411	410	565	154	53
13	**20b**	MeO	MeO	Me	BPh_2	337	387	453	116	43
14	**21b**	MeO	MeO	Me	$B(C_6F_5)_2$	346	372	440	94	48
15	**22b**	MeO	MeO	Me	$B(C_6F_5)_3$	357	368	465	108	57
16	**19a**	MeO	H	Me	BF_2	355		410	55	36
17	**19b**	MeO	MeO	Me	BF_2	371		415	45	47
18	**19c**	MeO	H	Ph	BF_2	378	372	487	109	55
19	**19d**	MeO	MeO	Ph	BF_2	396		458	62	54
20	**19e**	MeO	H	2- thienyl	BF_2	401		471	70	58
21	**19f**	MeO	MeO	2- thienyl	BF_2	410		480	70	62
22	**18j**	MeO	MeO	$4-O_2N-C_6H_4$	BF_2	370	-	-	-	60

* pyrrolidine-1-yl

3. X-Ray diffraction data for boroisoquinolines 18f and 19c

Indexing was performed from 4 oscillations that were exposed for 60 seconds. The crystal-to detector distance was 127.40 mm. The data were collected at a temperature of 20 + 1 °C to a maximum 2θ value of 143.3°. A total of 180 oscillation images were collected. A sweep of data was done using ω scans from 20.0 to 200.0° in 5.0° step, at χ=0.0° and φ = 0.0°. The exposure rate was 36.0 [sec./ °]. A second sweep was performed using ω scans from 20.0 to 200.0° in 5.0° step, at χ=54.0° and φ = 0.0°. The exposure rate was 36.0 [sec./ °]. Another sweep was performed using ω scans from 20.0 to 200.0° in 5.0° step, at χ=54.0° and φ = 90.0°. The exposure rate was 36.0 [sec./ °]. Another sweep was performed using ω scans from 20.0 to 200.0° in 5.0°step, at χ=54.0° and φ = 180.0°. The exposure rate was 36.0 [sec./ °]. Another sweep was performed using ω scans from 20.0 to 200.0° in 5.0° step, at χ=54.0° and φ = 270.0°. The exposure rate was 36.0 [sec./ °]. Readout was performed in the 0.100 mm pixel mode.

18f **19c**

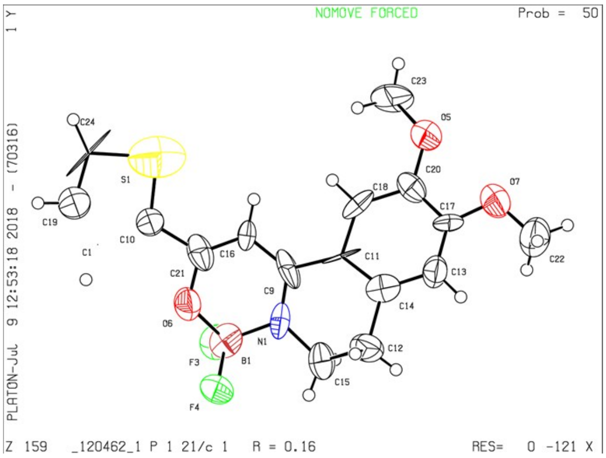

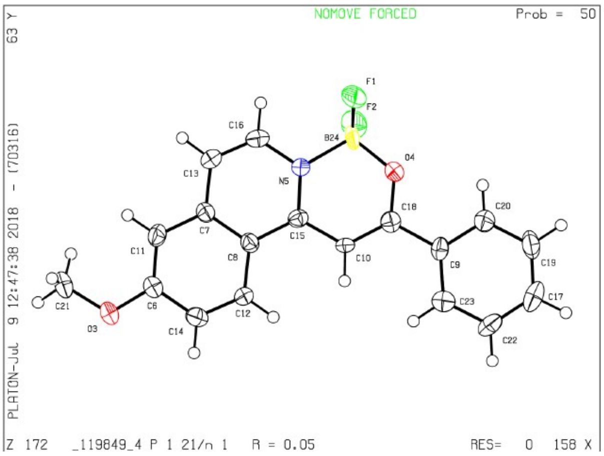

Crystal data and structural refinements for compounds 18f and 19c.

	**18f**	**19c**
**CCDC**	1854625	1854624
**Empirical formula**	$C_{17}H_{16}BF_2NO_3S$	$C_{18}H_{14}BF_2NO_2$
**Formula weight**	363.19	325.12
**Temperature/K**	293	293
**Wavelength (Å)**	1.54187	1.54187
**Crystal Size (mm)**	0.56 x 0.26 x 0.21	0.43 x 0.17 x 0.17
**Crystal system**	monoclinic	monoclinic
**Space group**	P21/c (#14)	P21/n (#14)
**a/Å**	12.1184(6)	7.7350(3)
**b/Å**	8.1766(4)	17.5815(7)
**c/Å**	16.5924(9)	11.0299(4)
**α/°**	90	90
**β/°**	93.697(3)	99.364(2)
**γ/°**	90	90
**$V/Å^3$**	1640.68(14)	1480.01(10)
**Z/D_{calcd} (g/cm^3)**	4/1.470	4/1.459
**μ/cm^{-1}**	21.050	9.326
**h, k, l_{max}**	14, 10, 20	9, 21, 13
**Final R indices [I >2 σ(I)]**	R = 0.1597, R_w = 0.1982	R = 0.0507, R_w = 0.0665
**S**	3.595	6.953
**Max./min. $e^-/Å^{-3}$**	9.94/-20.10	4.22/-4.25

4. $^1H/^{13}C$ NMR spectra

6-Bromo-1-methyl-3,4-dihydroisoquinoline (**16c**)

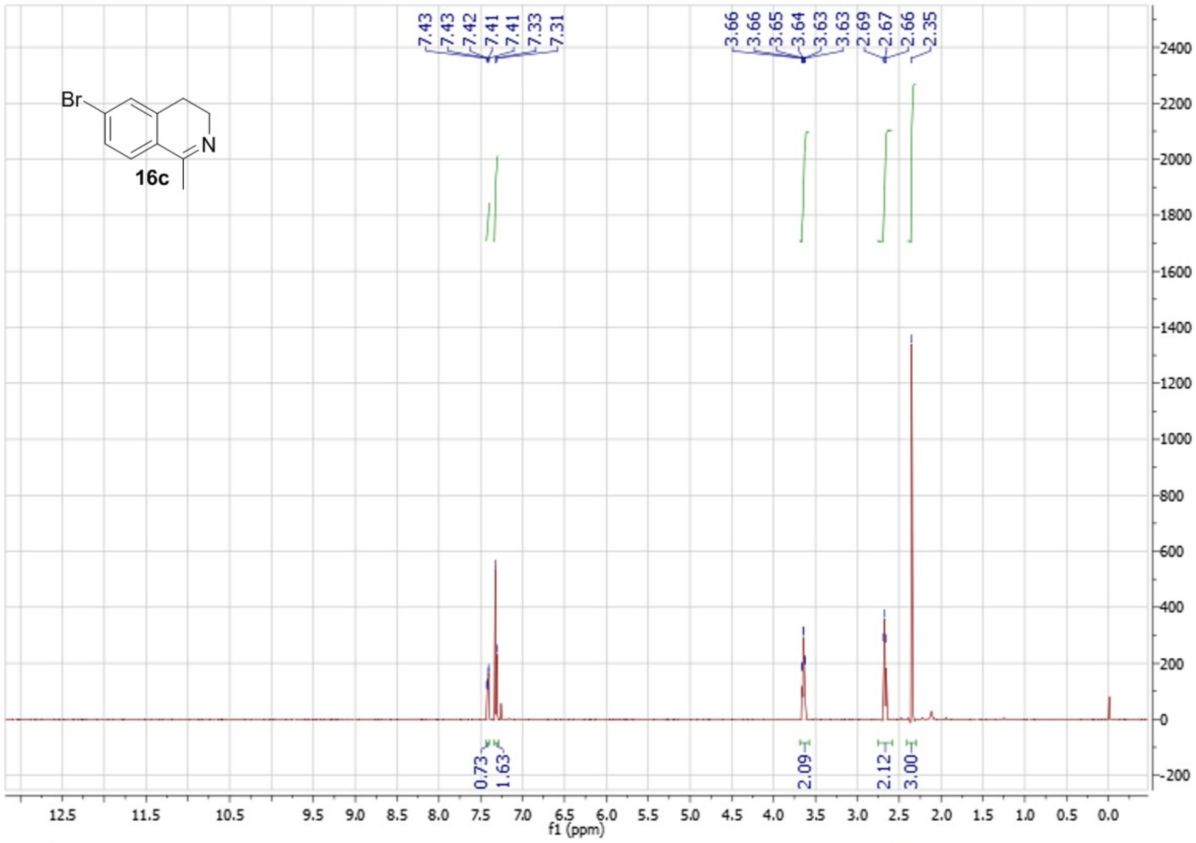
7.43
7.43
7.42
7.41
7.41
7.33
7.31
3.66
3.66
3.65
3.64
3.63
3.63
2.69
2.67
2.66
2.35
Br
N
16c
0.73
1.63
2.09
2.12
3.00
f1 (ppm)

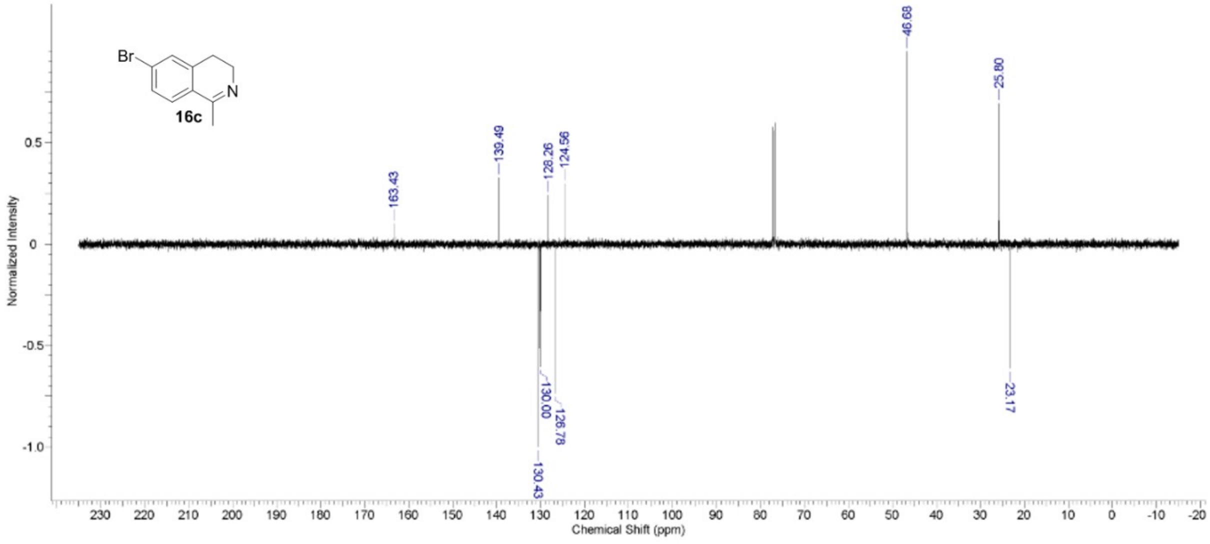
Br
N
16c
163.43
139.49
128.26
124.56
46.68
25.80
130.00
126.78
130.43
23.17
Normalized Intensity
Chemical Shift (ppm)

6-Fluoro-1-methyl-3,4-dihydroisoquinoline (**16d**)

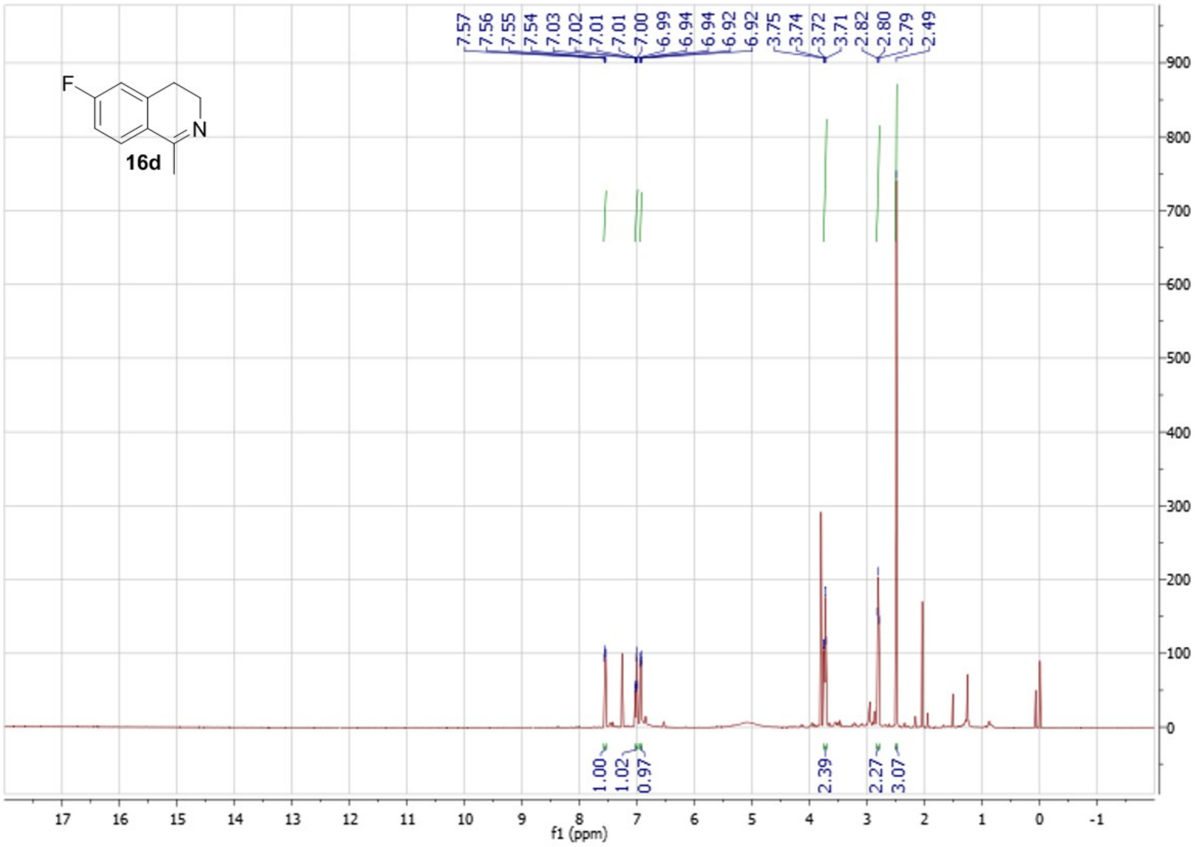

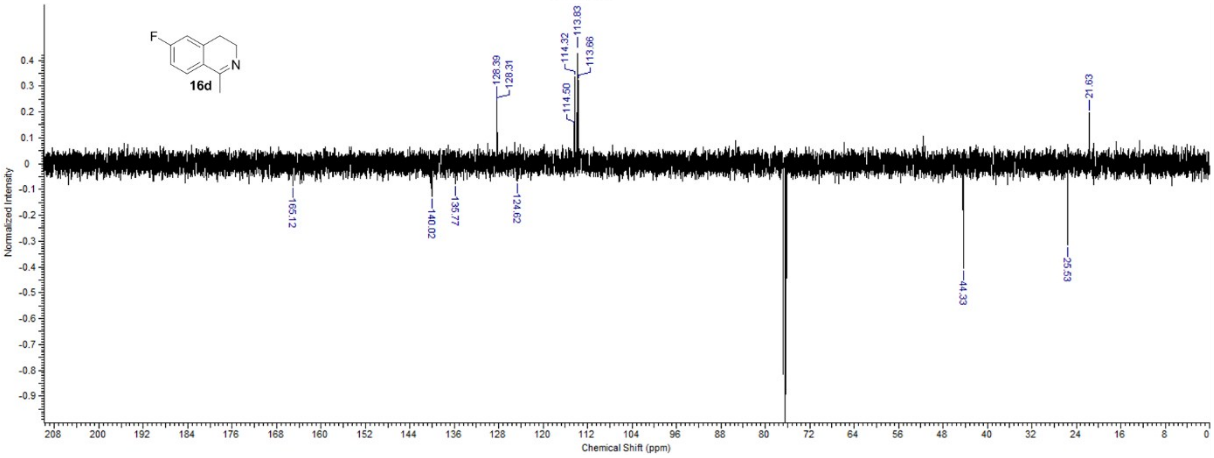

1-methyl-6-(pyrrolidin-1-yl)-3,4-dihydroisoquinoline (**16e**)

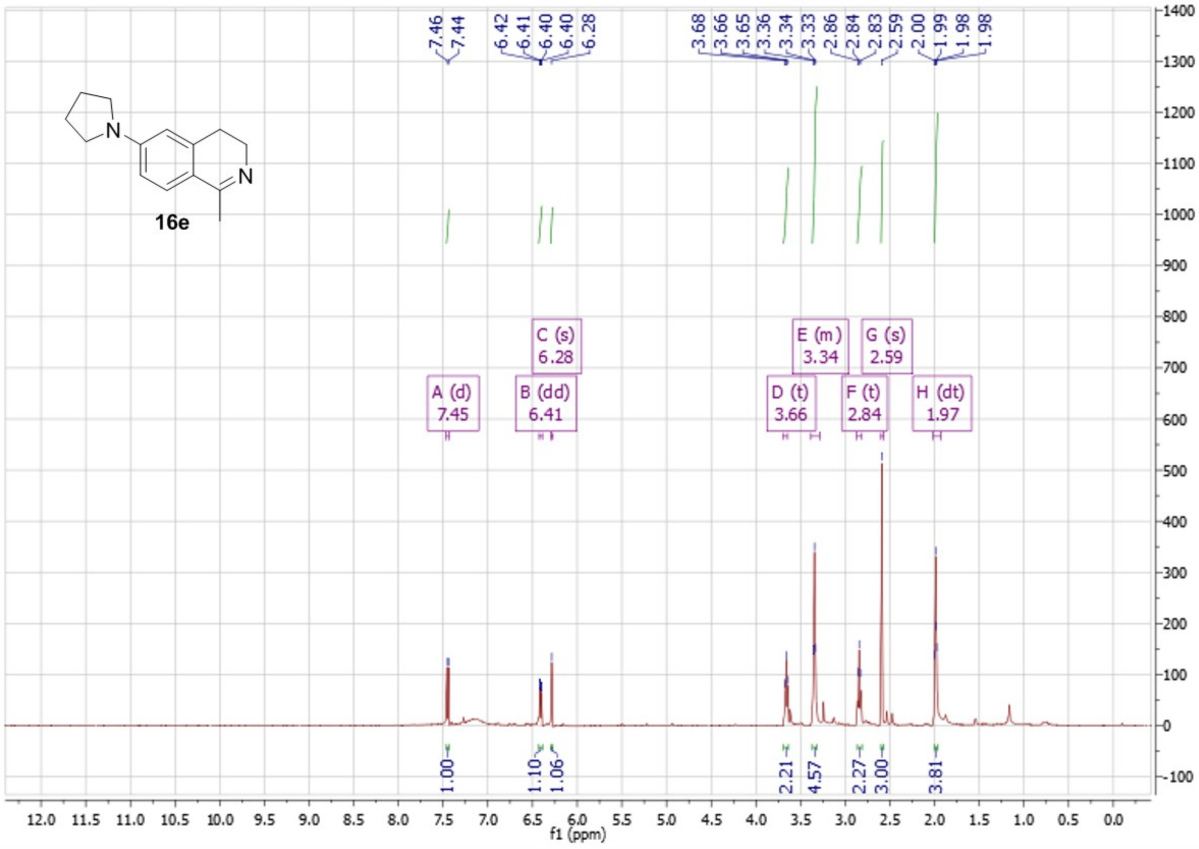

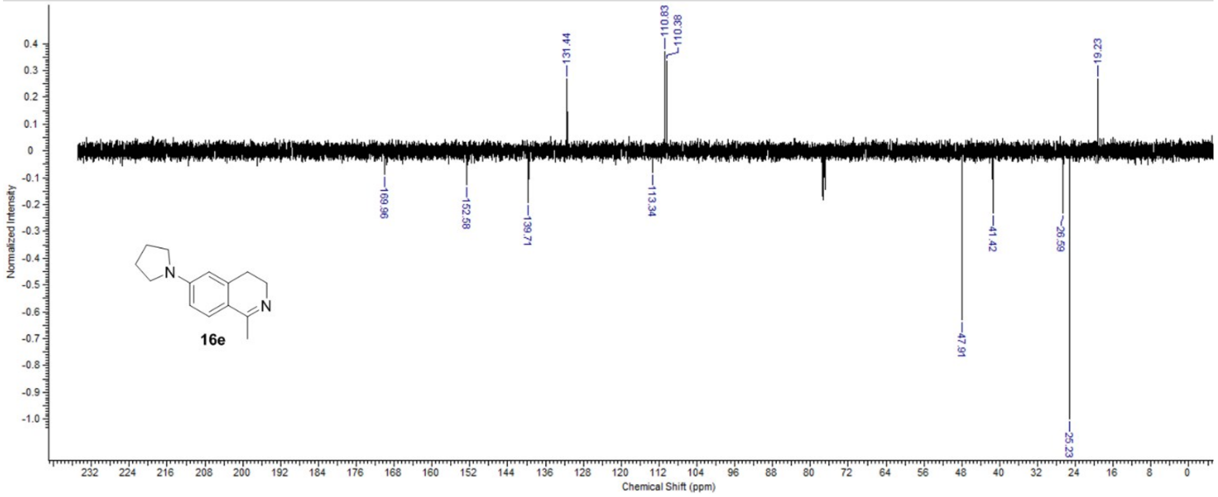

(Z)-2-(6-methoxy-3,4-dihydroisoquinolin-1(2H)-ylidene)-1-(perfluorophenyl)ethan-1-one **(17g)**

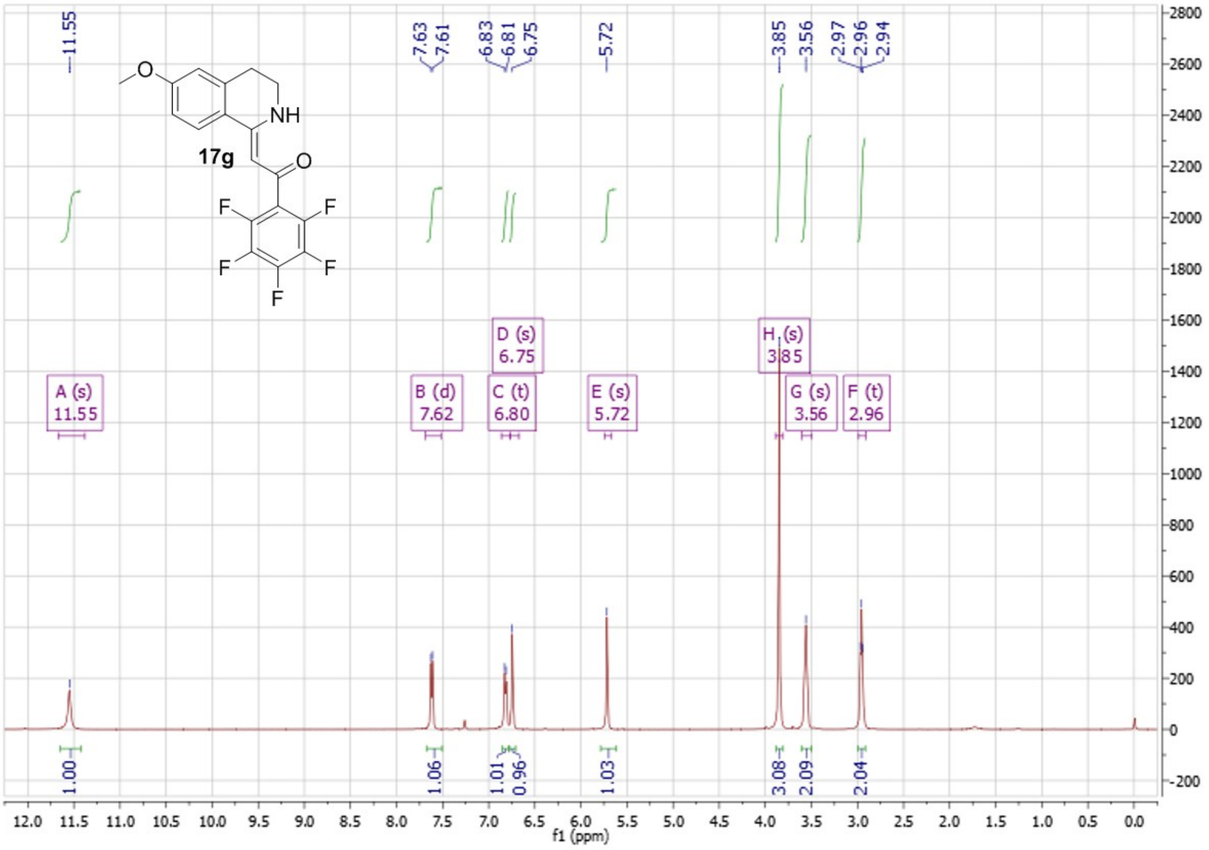

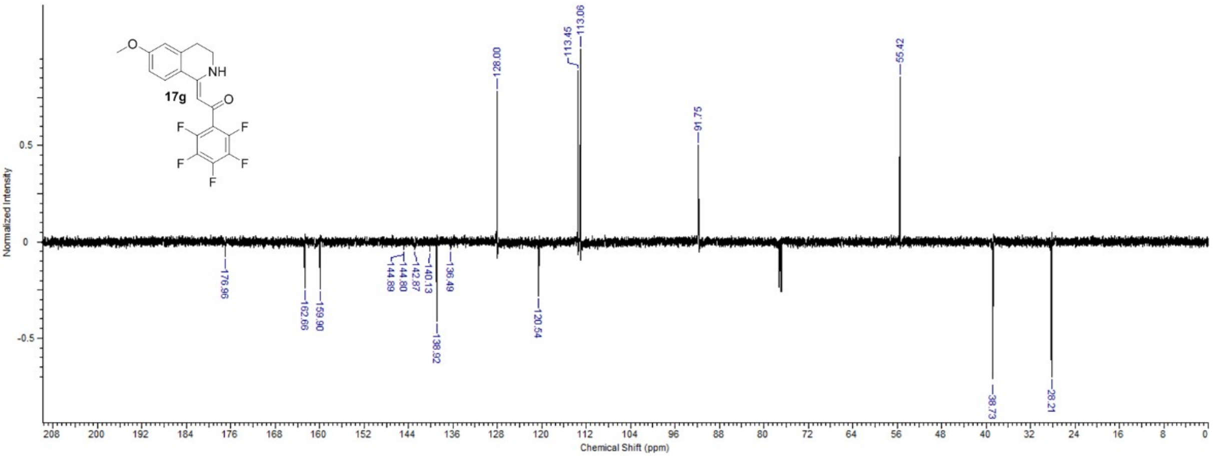

(Z)-2-(6,7-dimethoxy-3,4-dihydroisoquinolin-1(2H)-ylidene)-1-(perfluorophenyl)ethan-1-one **(17h)**

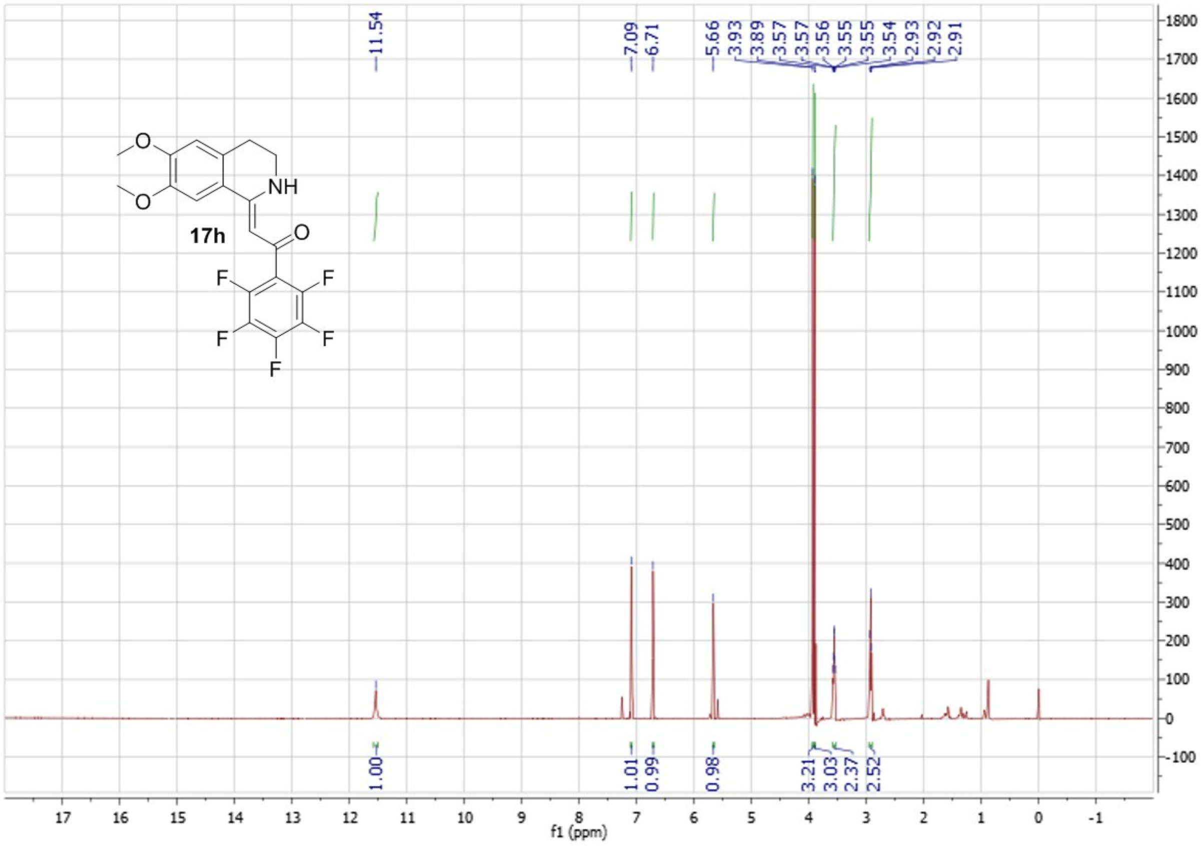

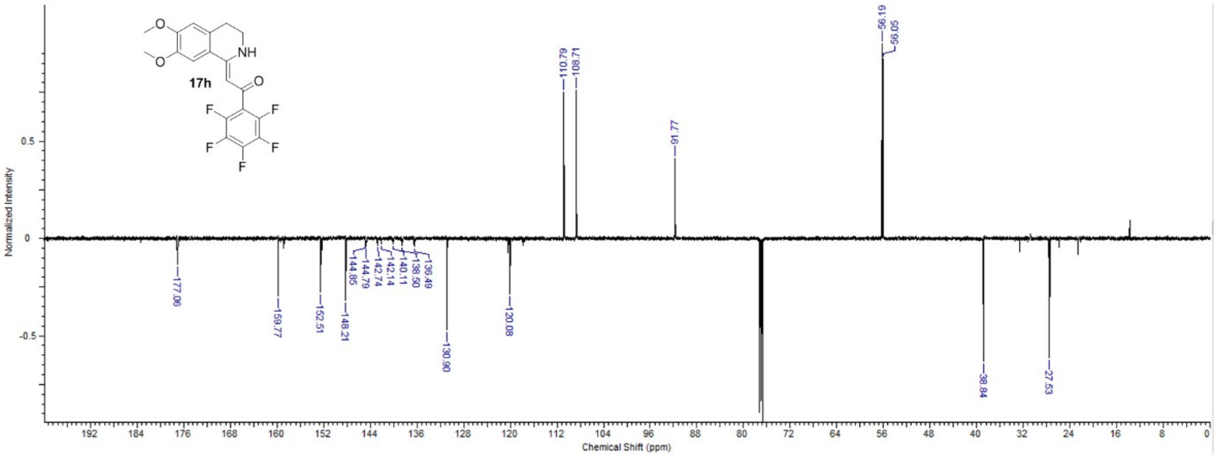

(Z)-1-(6-(pyrrolidin-1-yl)-3,4-dihydroisoquinolin-1(2H)-ylidene)propan-2-one (**17k**)

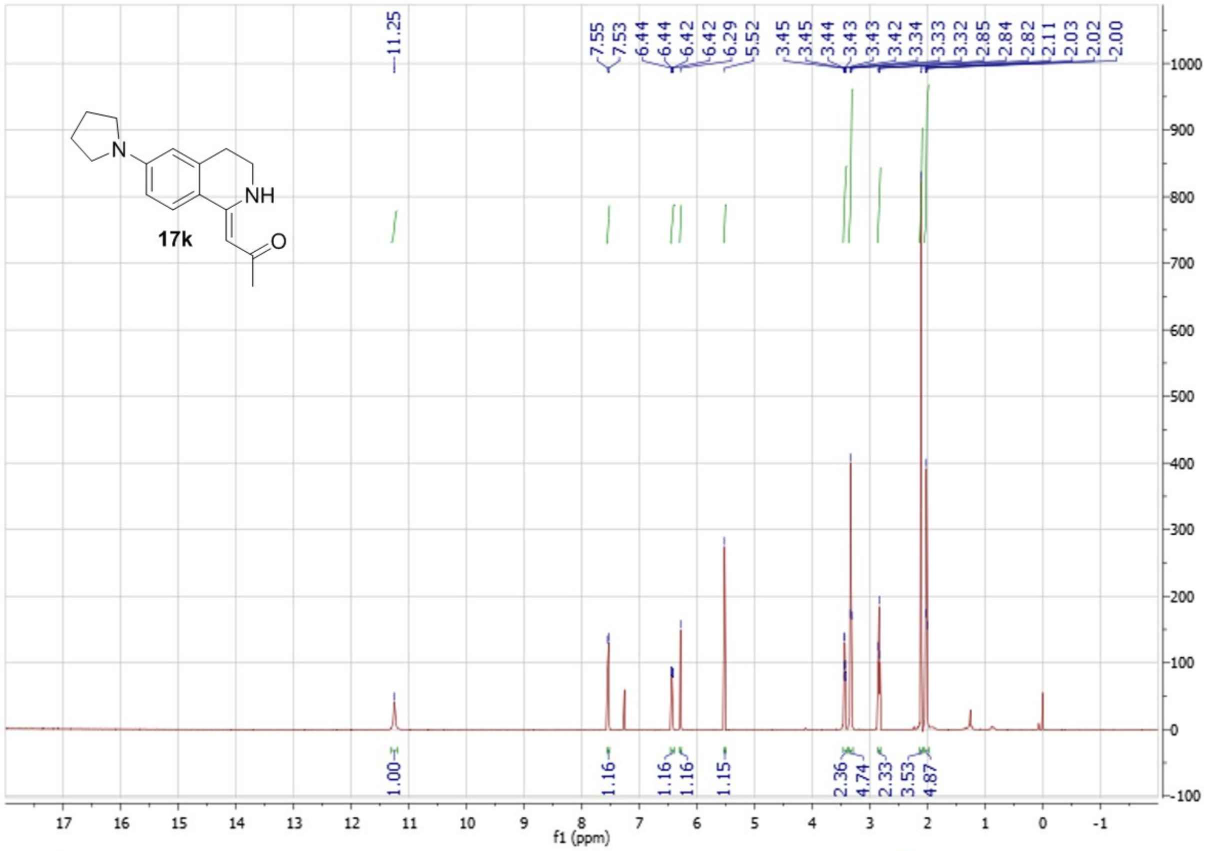

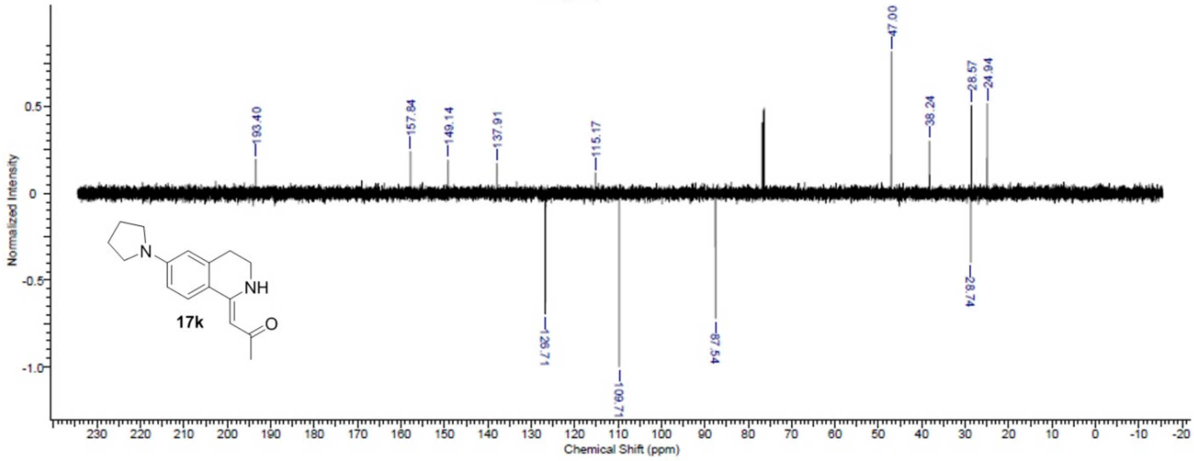

(Z)-1-phenyl-2-(6-(pyrrolidin-1-yl)-3,4-dihydroisoquinolin-1(2H)-ylidene)ethan-1-one (**17l**)

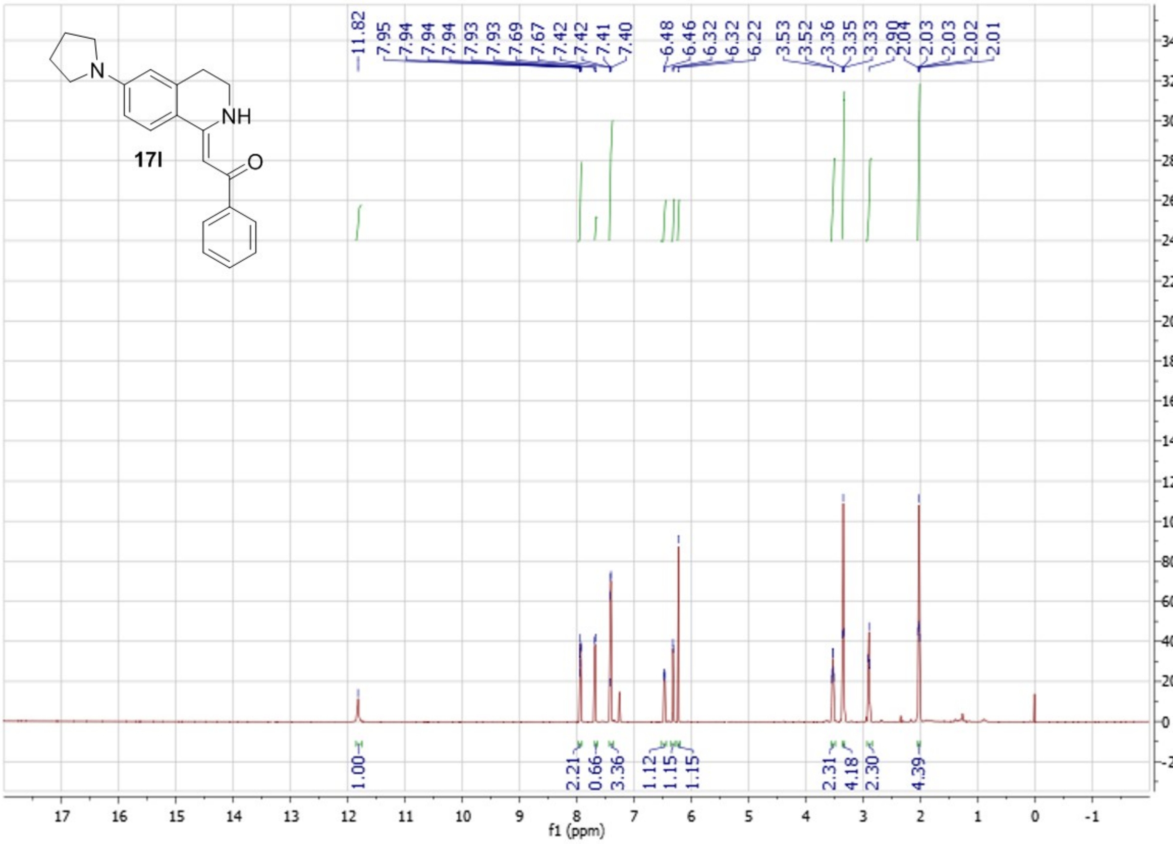

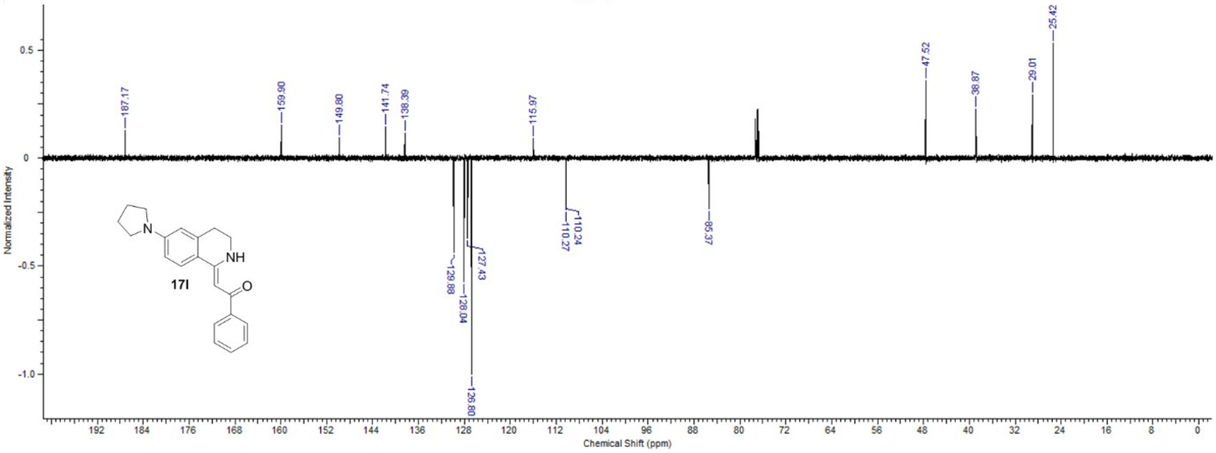

(Z)-1-(perfluorophenyl)-2-(6-(pyrrolidin-1-yl)-3,4-dihydroisoquinolin-1(2H)-ylidene)ethan-1-one (**17m**)

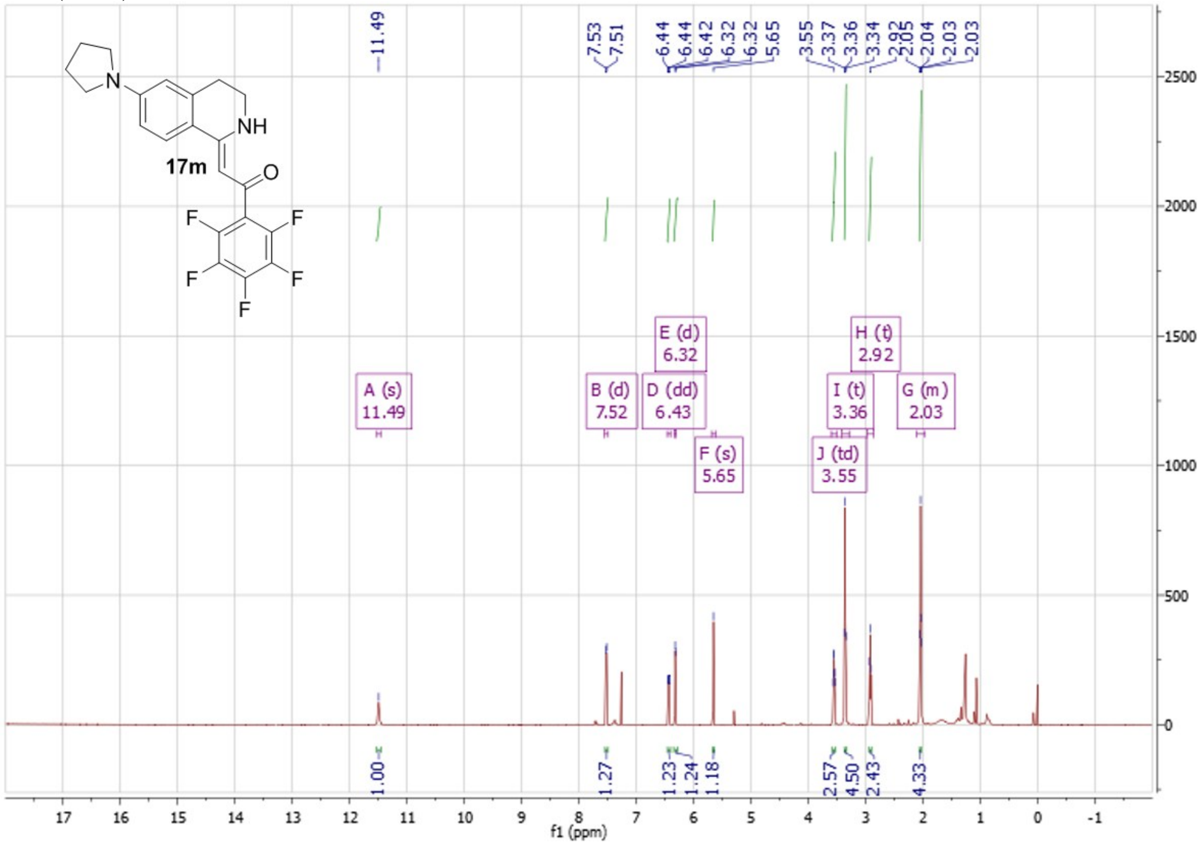

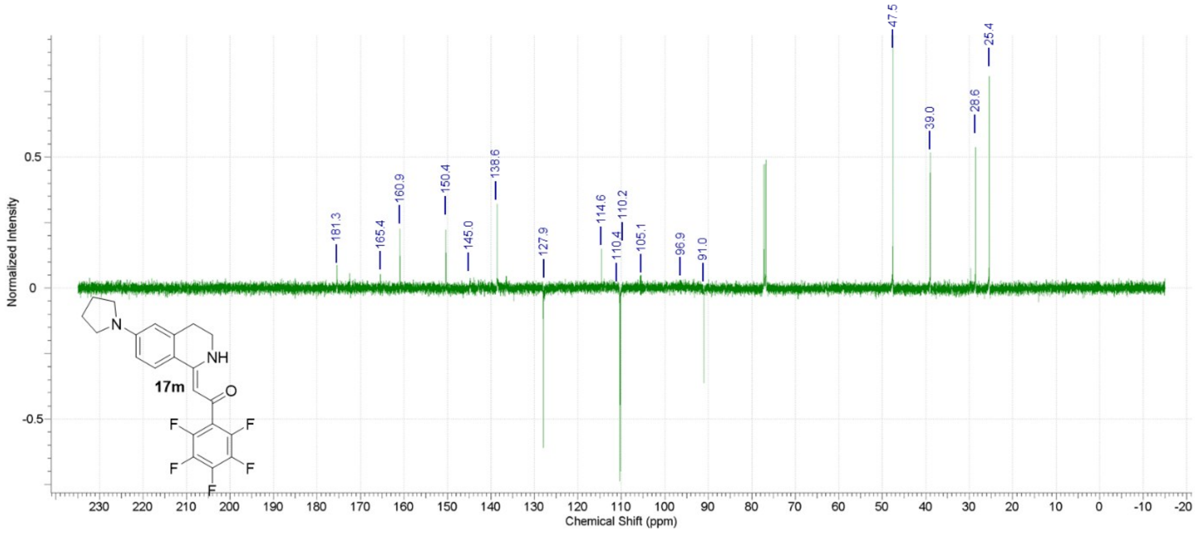

(Z)-1-(2-(difluoroboranyl)-6-methoxy-3,4-dihydroisoquinolin-1(2H)-ylidene)propan-2-one **(18a)**

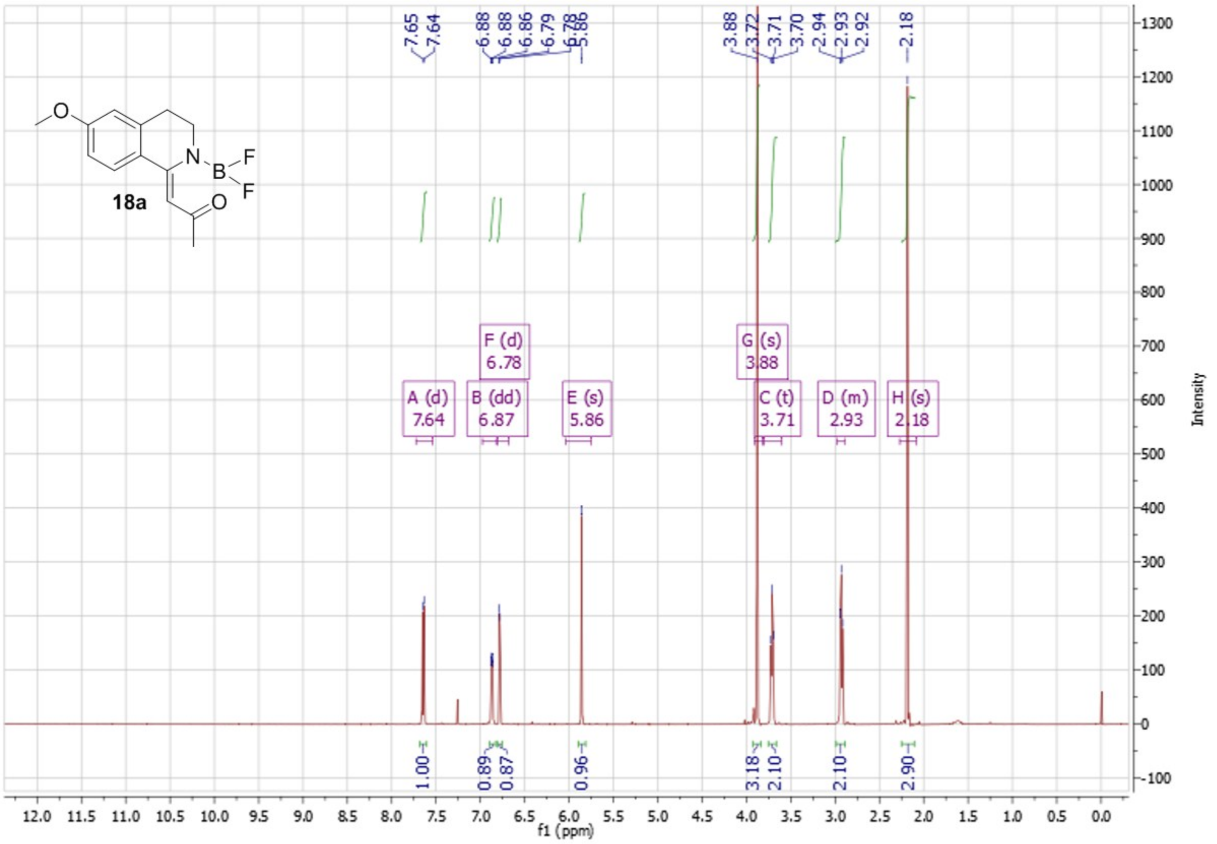

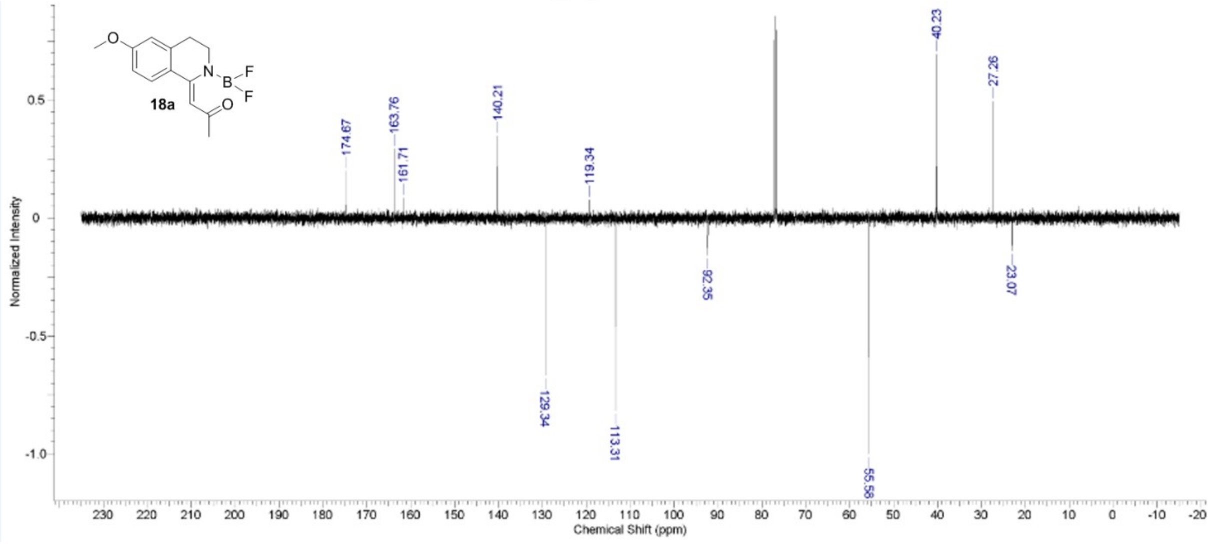

(Z)-1-(2-(difluoroboranyl)-6,7-dimethoxy-3,4-dihydroisoquinolin-1(2H)-ylidene)propan-2-one (**18b**)

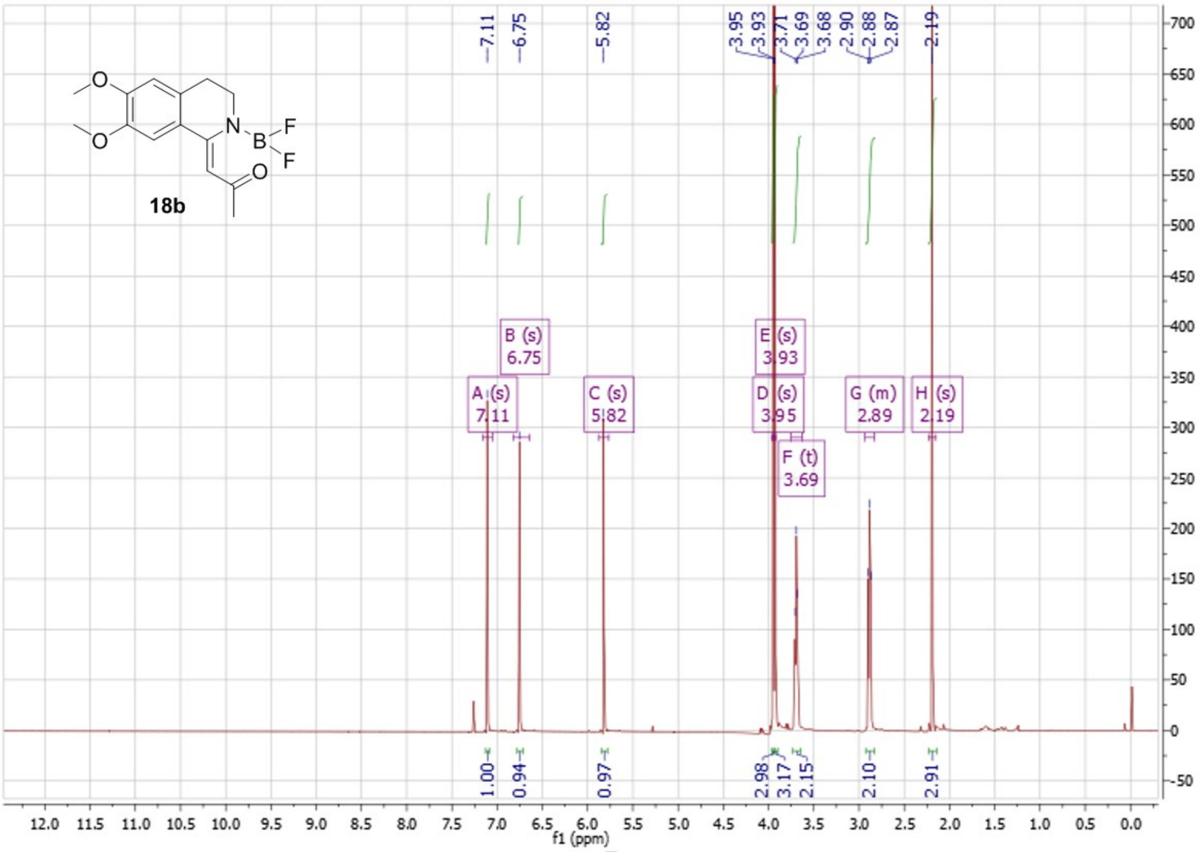

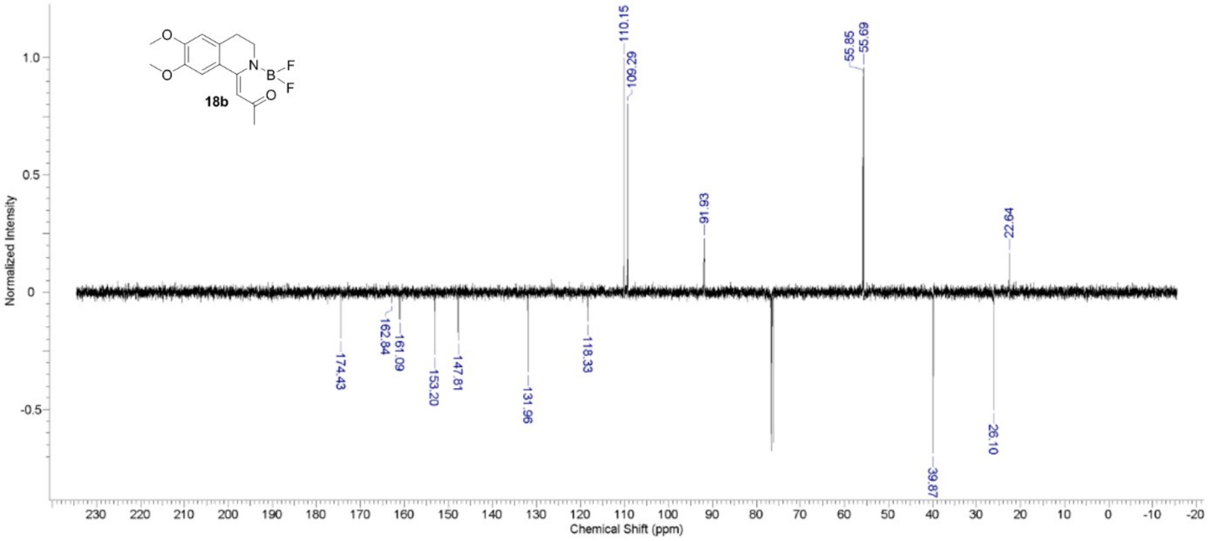

(Z)-2-(2-(difluoroboranyl)-6-methoxy-3,4-dihydroisoquinolin-1(2H)-ylidene)-1-phenylethan-1-one (**18c**)

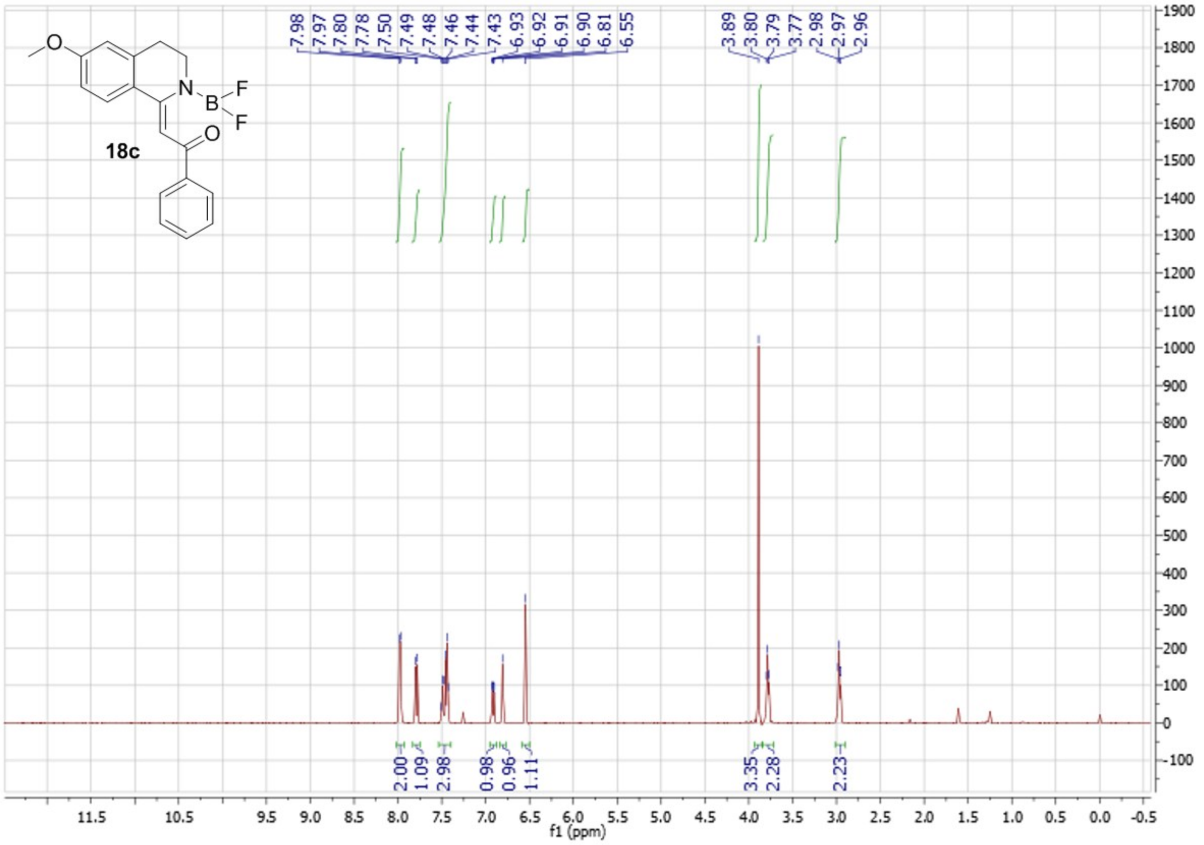

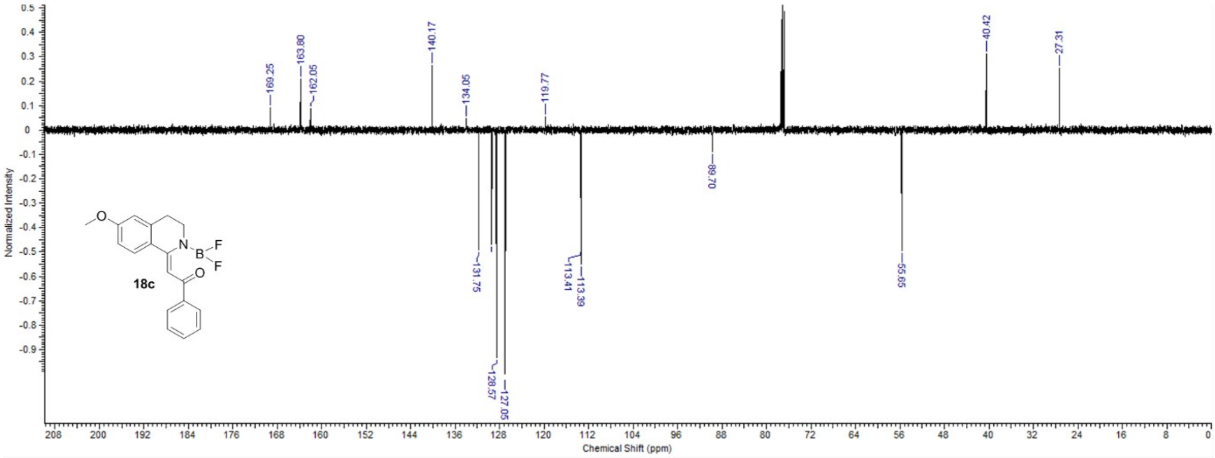

(Z)-2-(2-(difluoroboranyl)-6,7-dimethoxy-3,4-dihydroisoquinolin-1(2H)-ylidene)-1-phenylethan-1-one (**18d**)

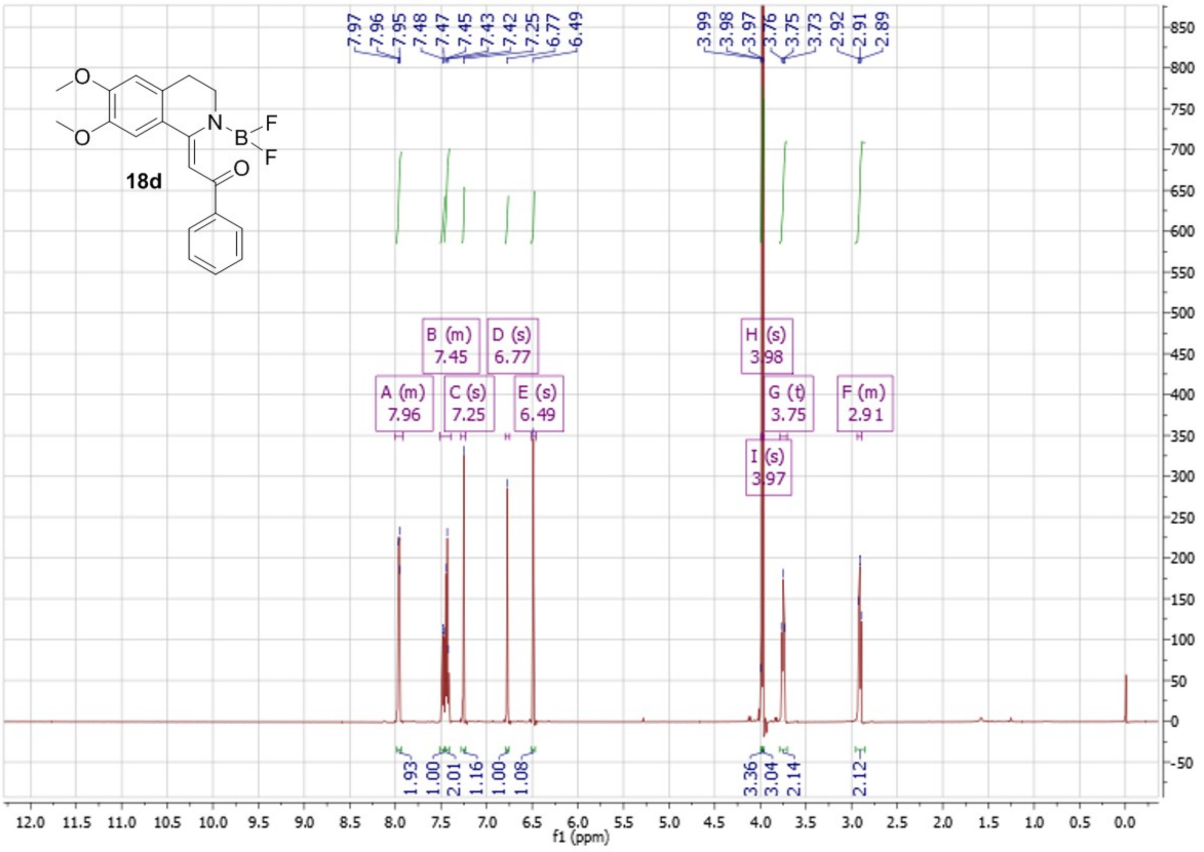

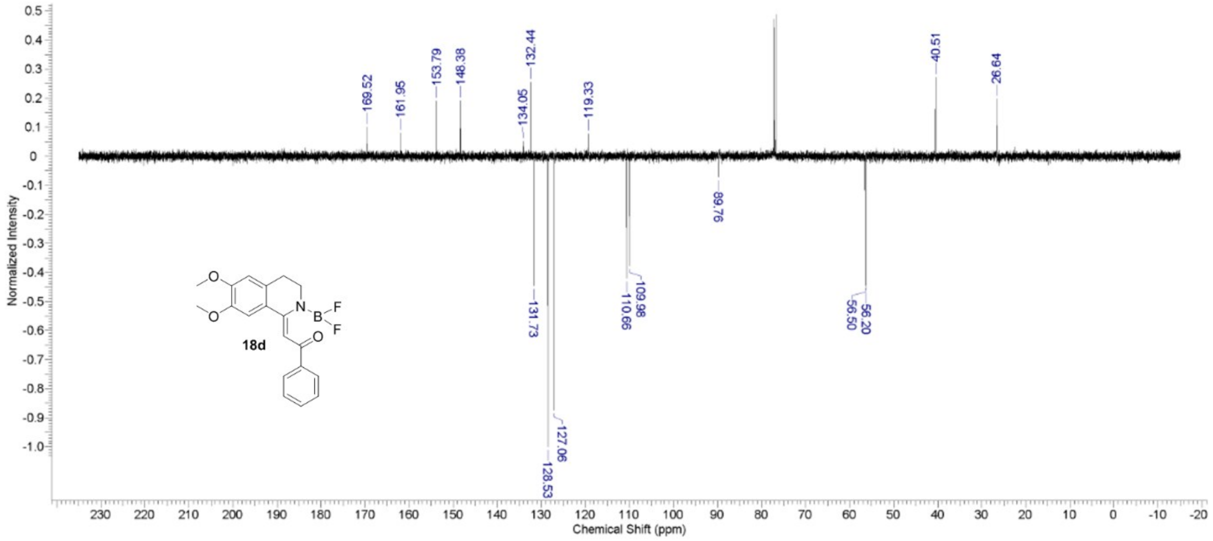

(Z)-2-(2-(difluoroboranyl)-6-methoxy-3,4-dihydroisoquinolin-1(2H)-ylidene)-1-(thiophen-2-yl)ethan-1-one (**18e**)

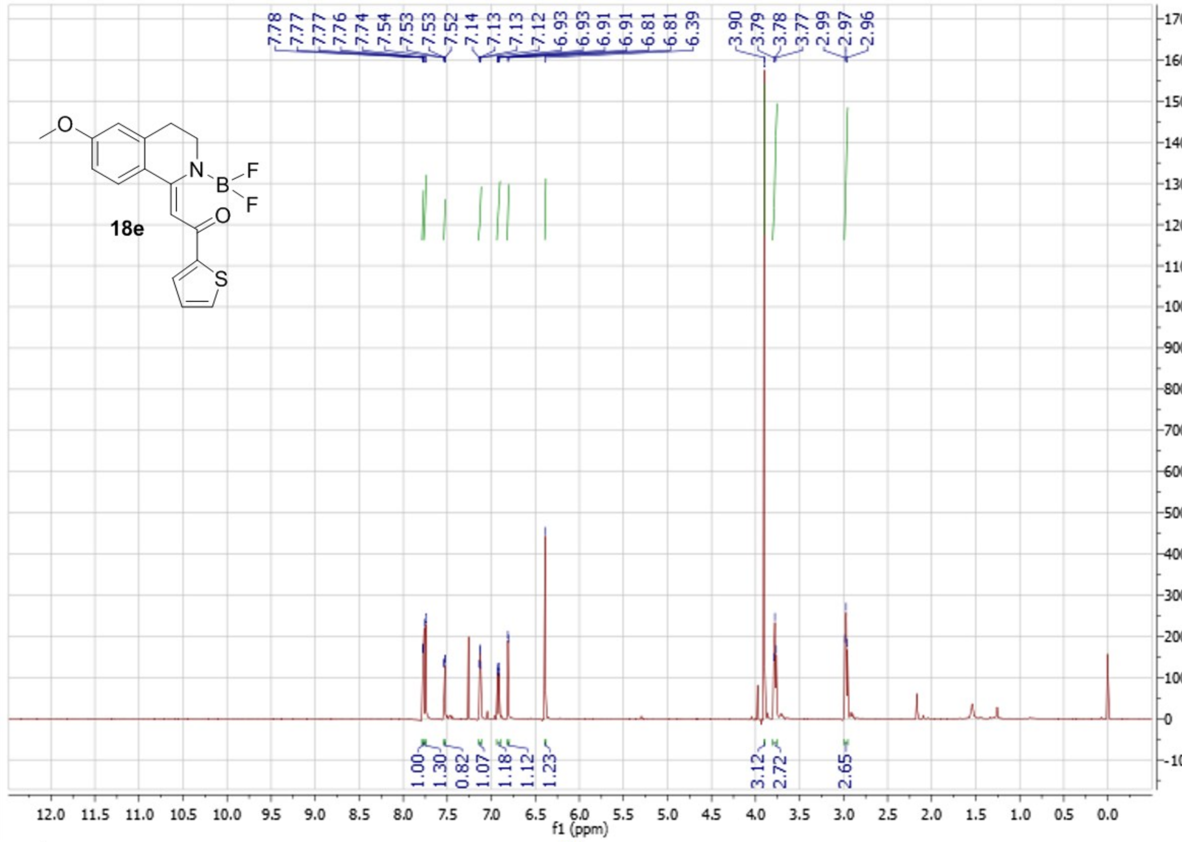

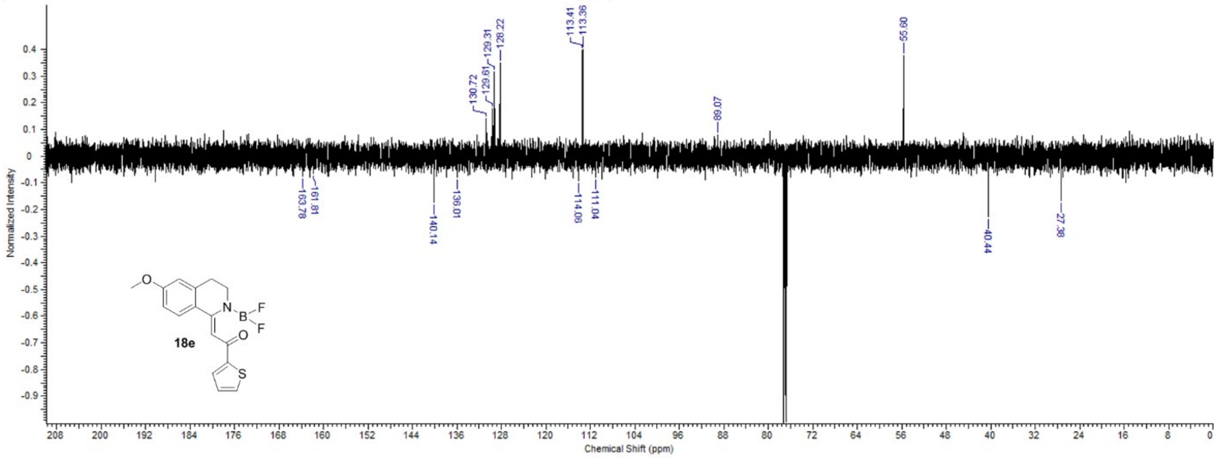

(Z)-2-(2-(difluoroboranyl)-6,7-dimethoxy-3,4-dihydroisoquinolin-1(2H)-ylidene)-1-(thiophen-2-yl)ethan-1-one (**18f**)

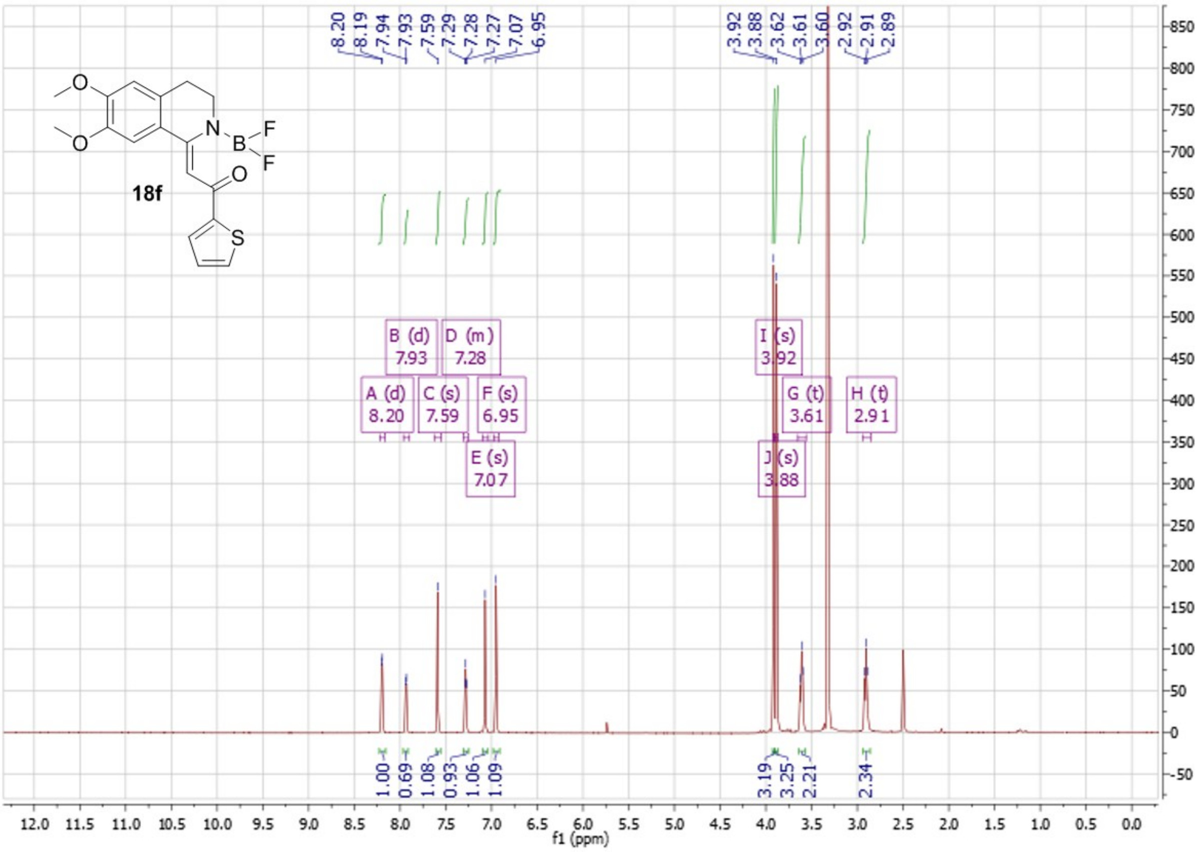

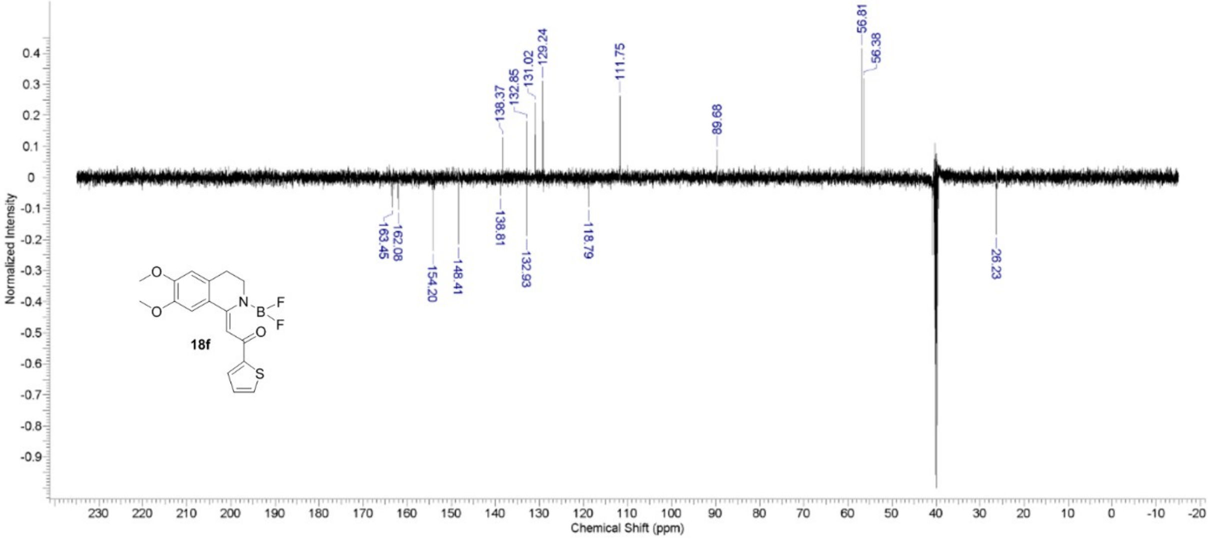

(Z)-2-(2-(difluoroboranyl)-6-methoxy-3,4-dihydroisoquinolin-1(2H)-ylidene)-1-(perfluorophenyl)ethan-1-one (**18g**)

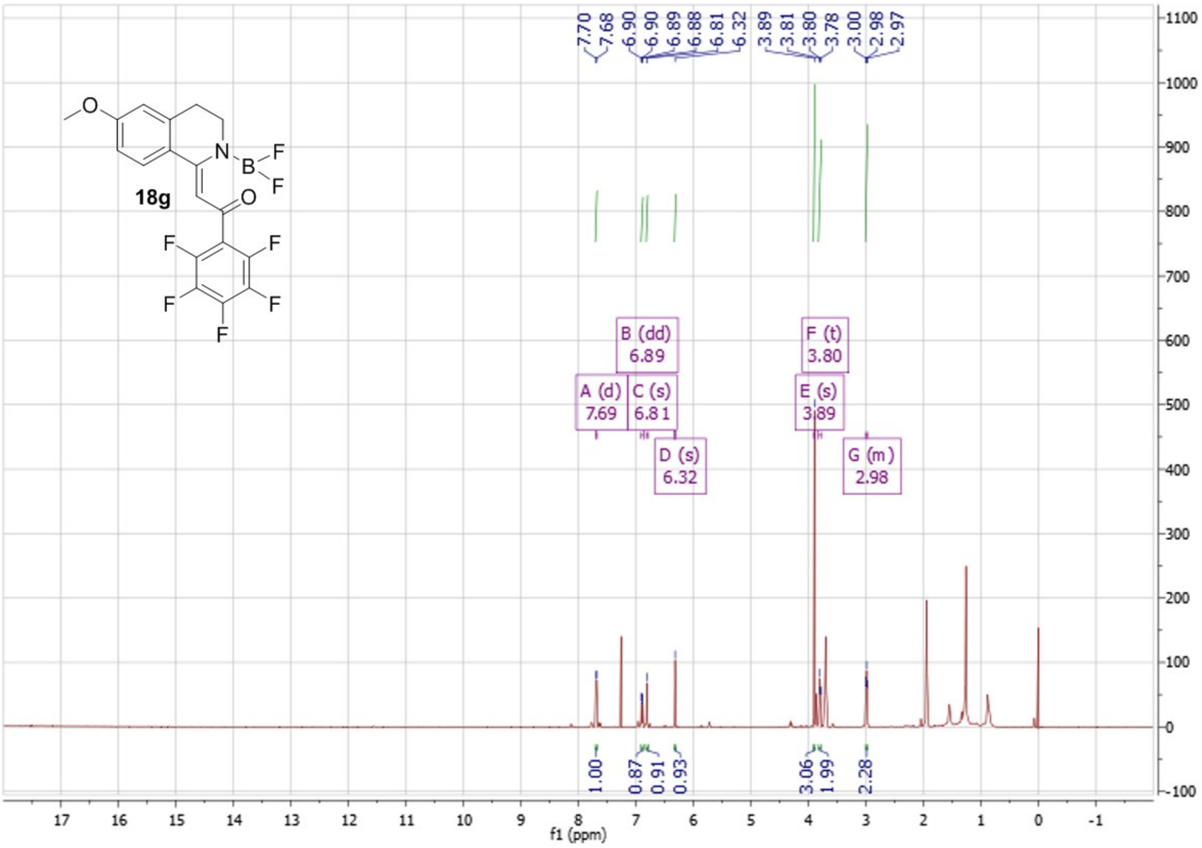

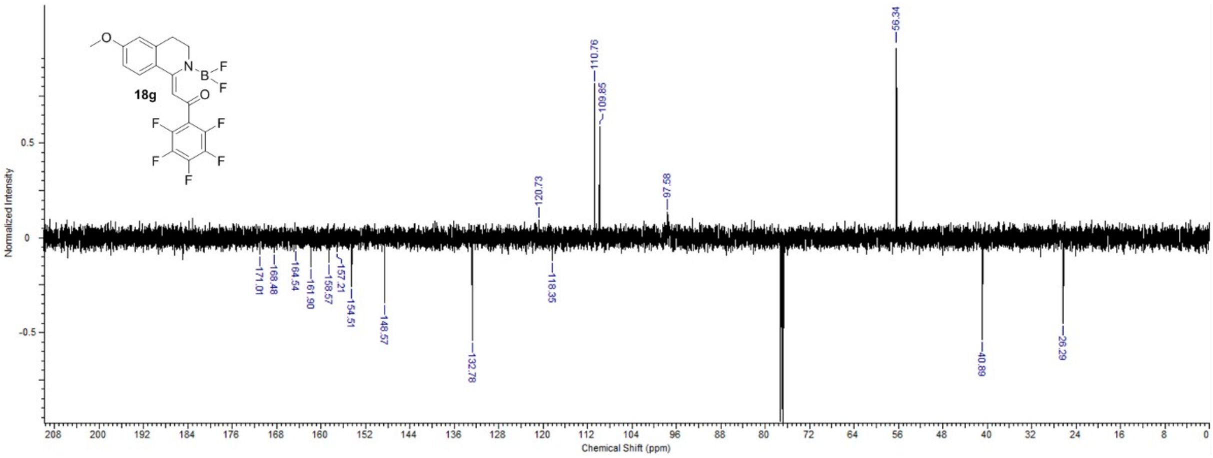

(Z)-2-(2-(difluoroboranyl)-6,7-dimethoxy-3,4-dihydroisoquinolin-1(2H)-ylidene)-1-(perfluorophenyl)ethan-1-one (**18h**)

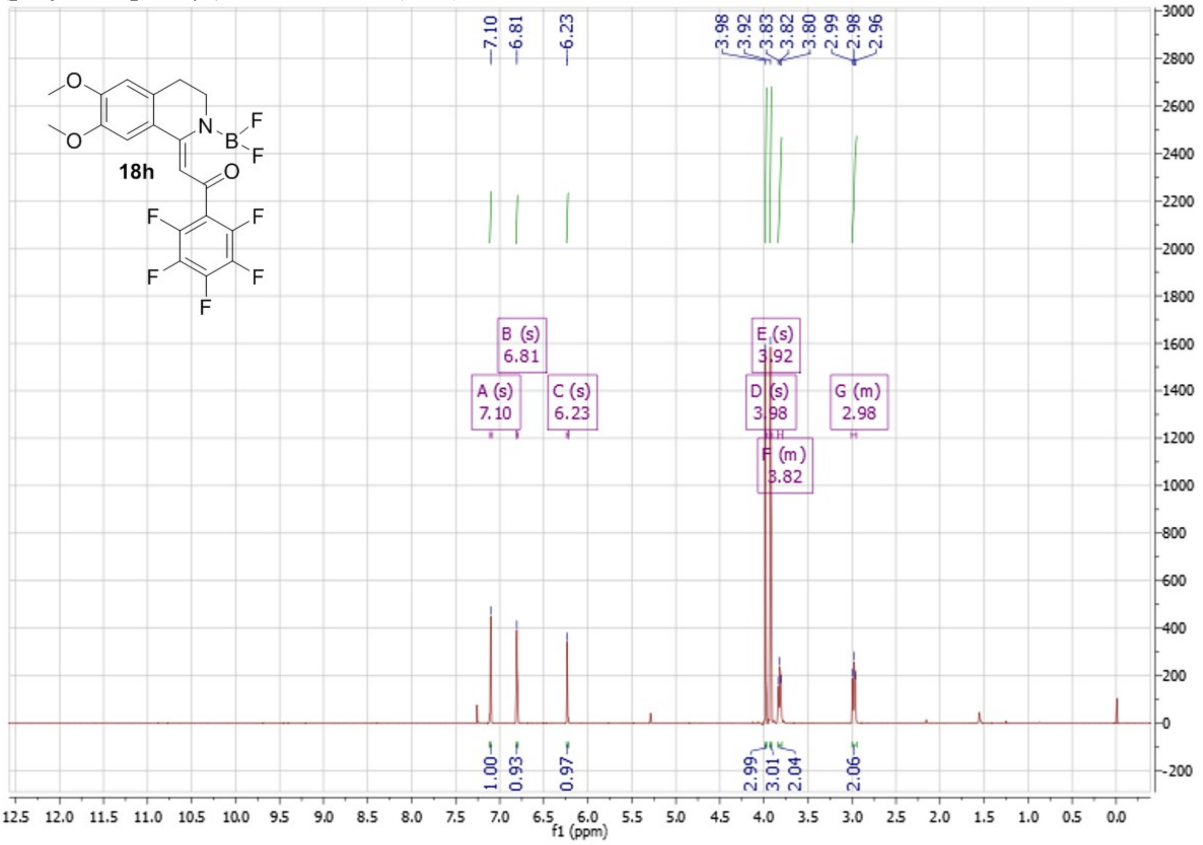

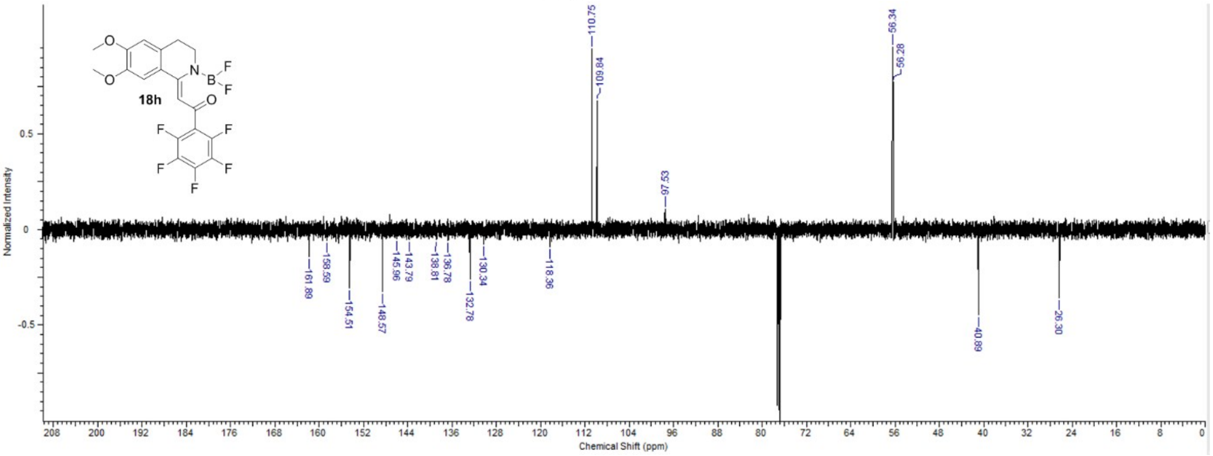

(Z)-2-(2-(difluoroboranyl)-6,7-dimethoxy-3,4-dihydroisoquinolin-1(2H)-ylidene)-1-(4-nitrophenyl)ethan-1-one (**18j**)

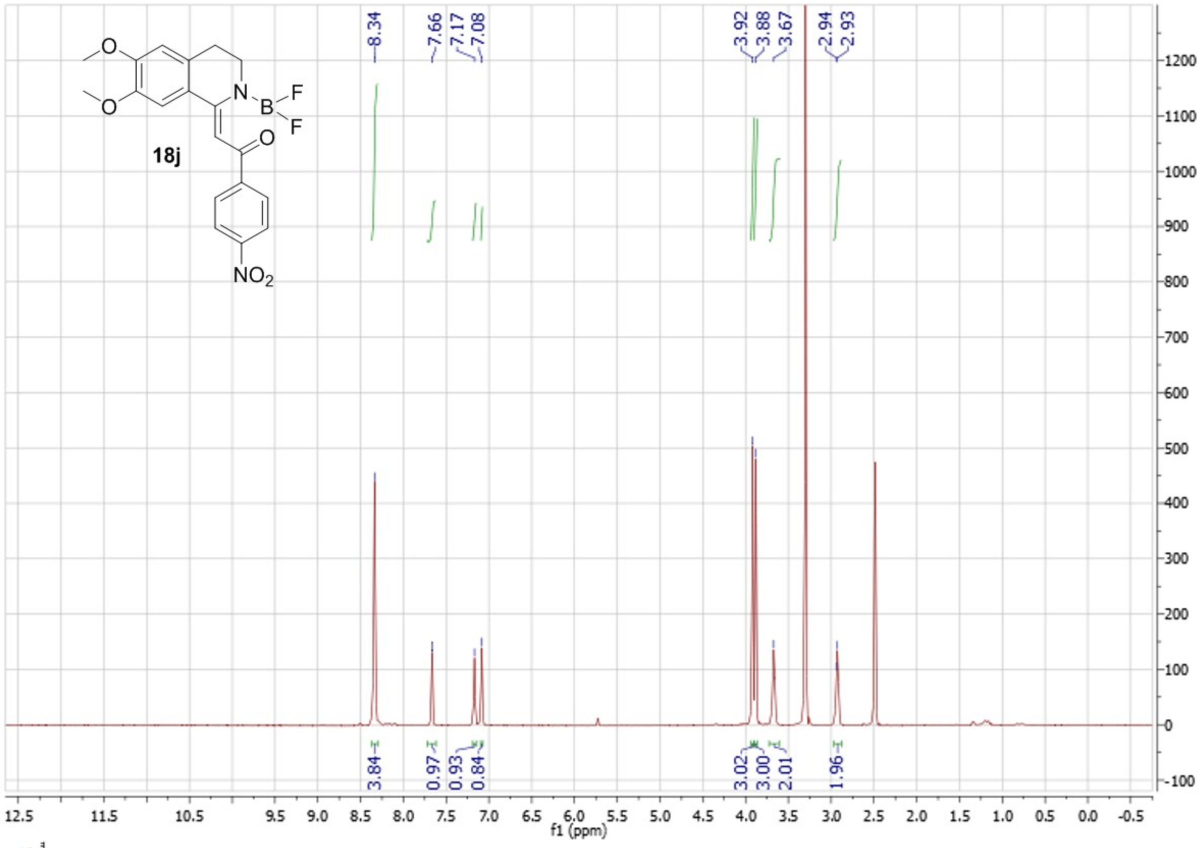

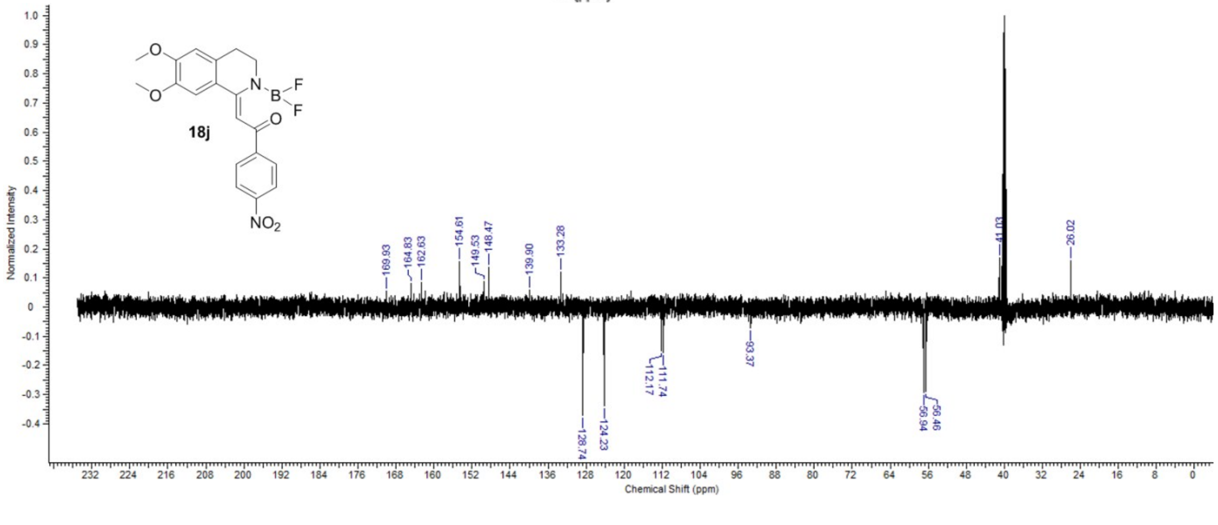

(Z)-1-(2-(difluoroboranyl)-6-(pyrrolidin-1-yl)-3,4-dihydroisoquinolin-1(2H)-ylidene)propan-2-one (**18k**)

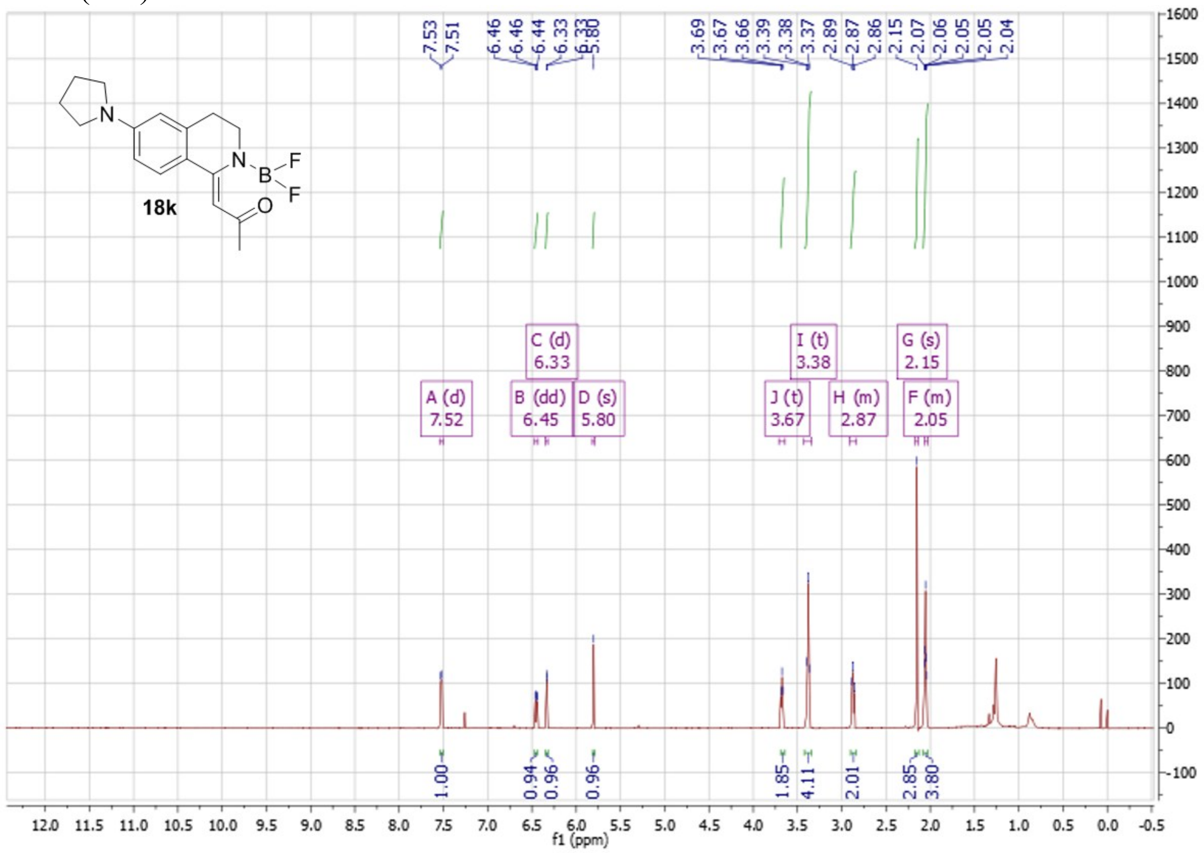

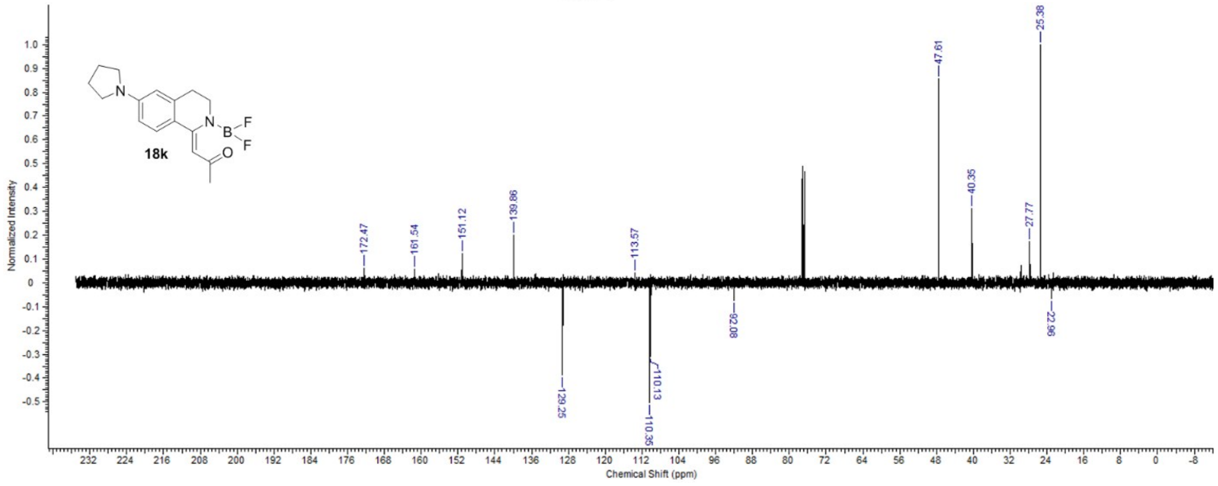

(Z)-2-(2-(difluoroboranyl)-6-(pyrrolidin-1-yl)-3,4-dihydroisoquinolin-1(2H)-ylidene)-1-phenylethan-1-one (**18l**)

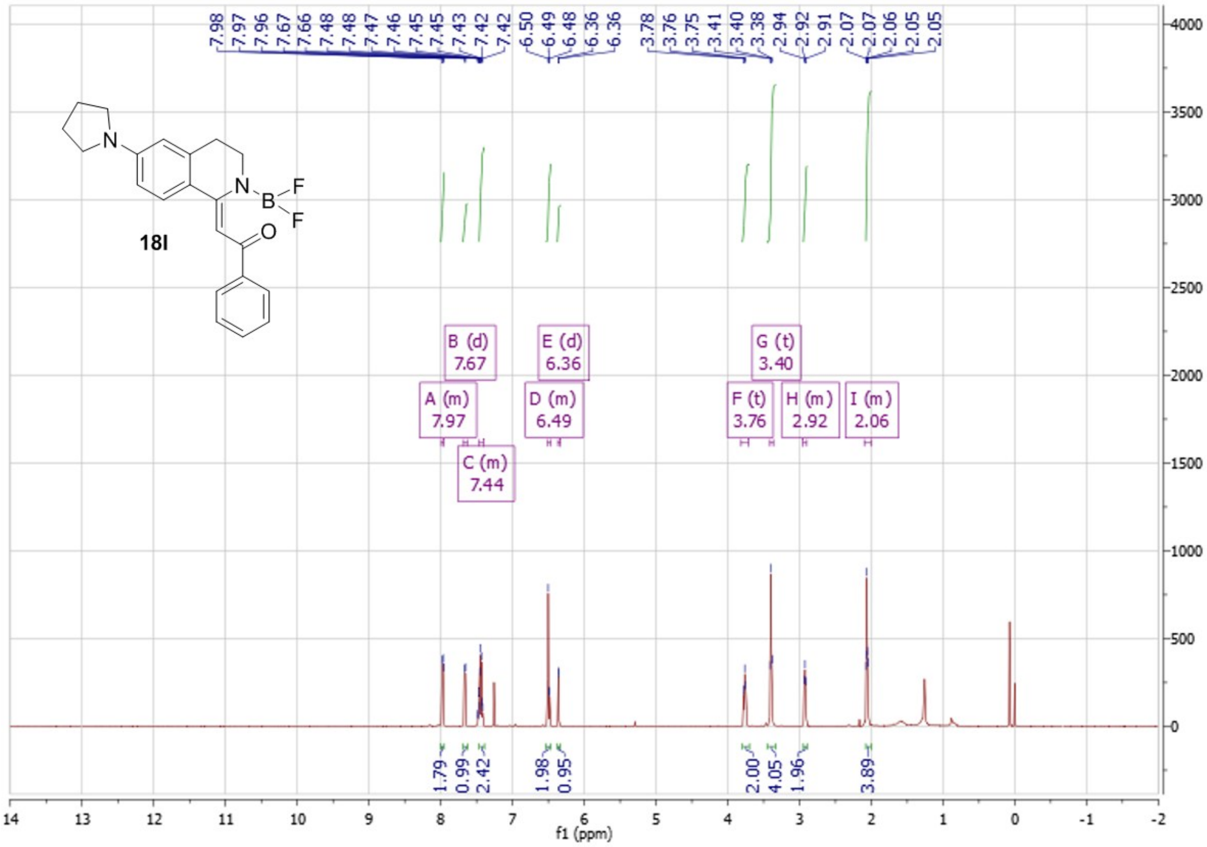

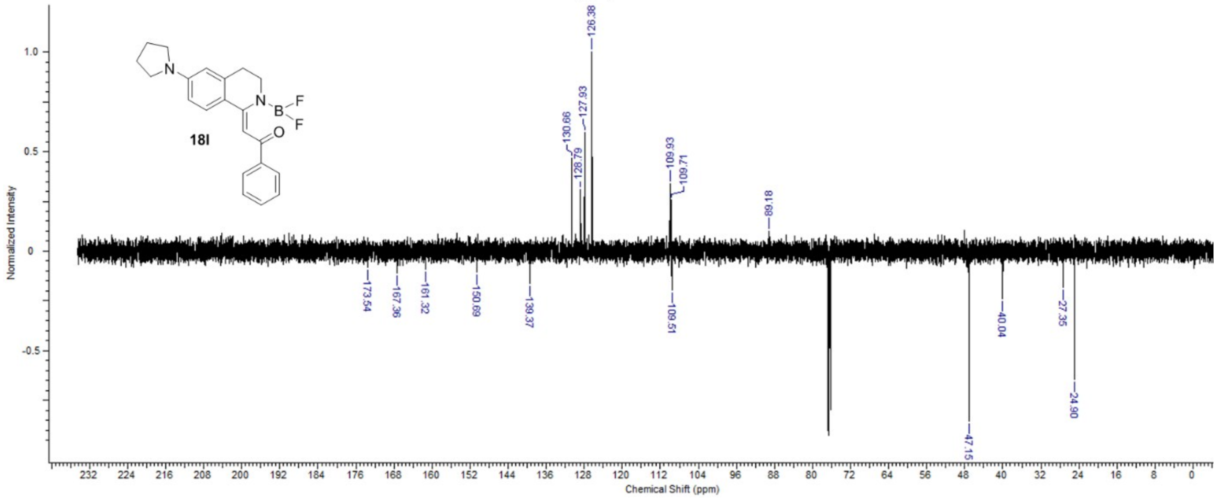

(Z)-2-(2-(difluoroboranyl)-6-(pyrrolidin-1-yl)-3,4-dihydroisoquinolin-1(2H)-ylidene)-1-(perfluorophenyl)ethan-1-one (**18m**)

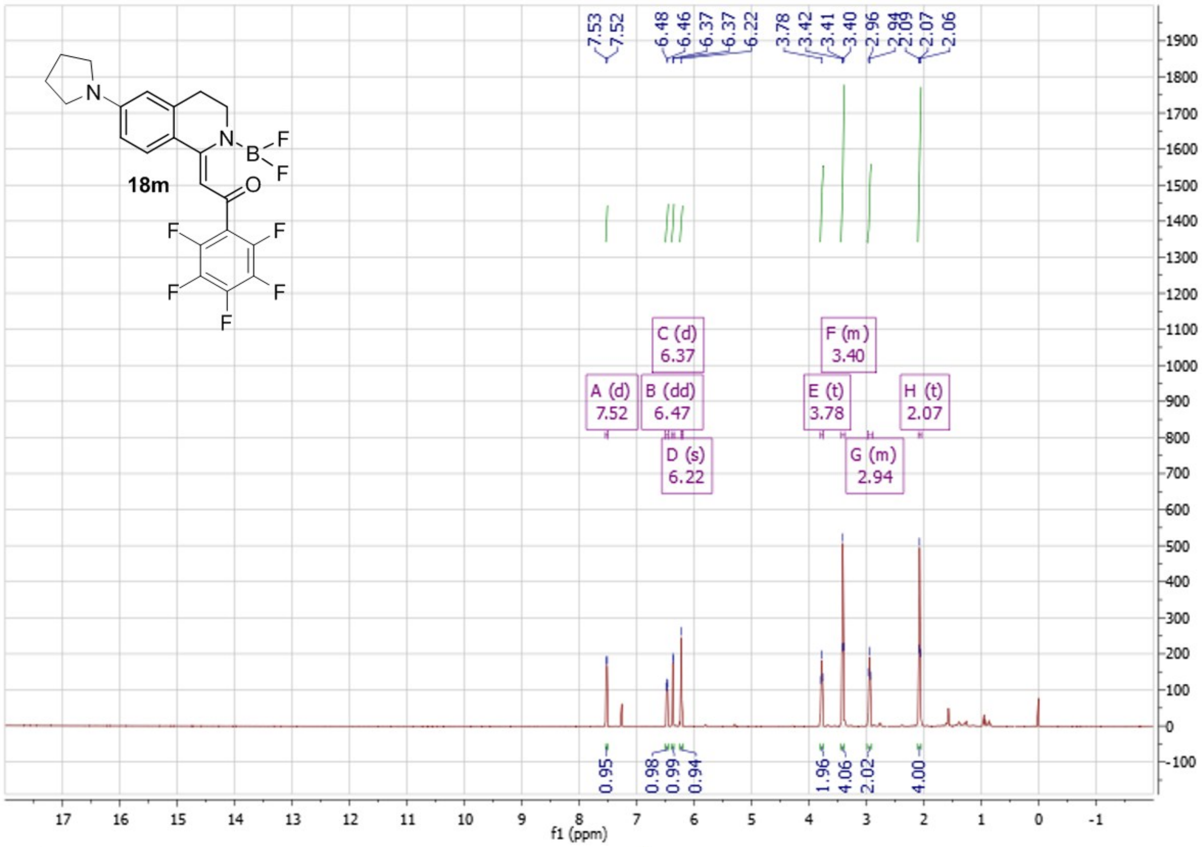

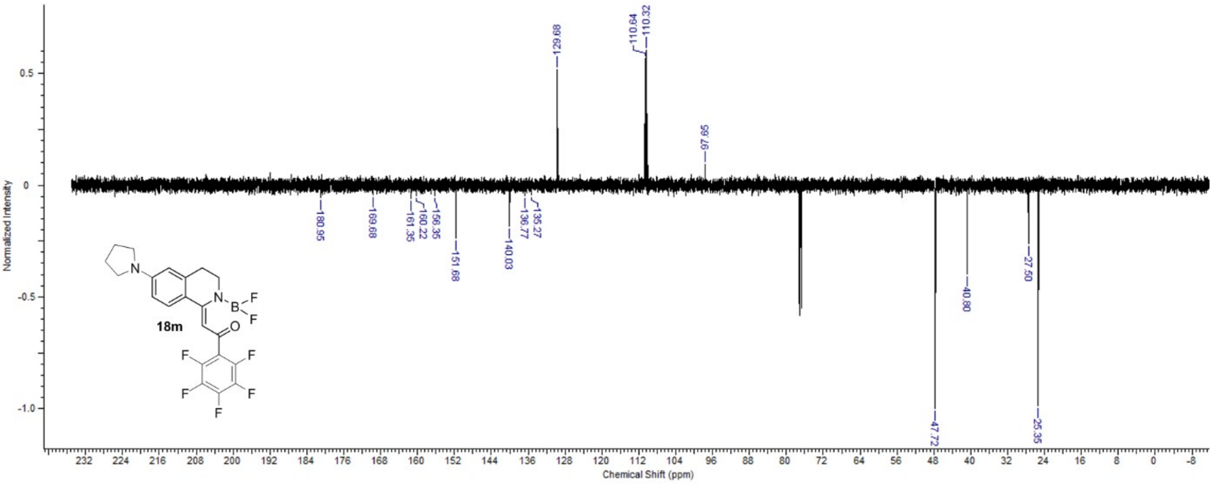

(Z)-1-(2-(difluoroboranyl)-6-methoxyisoquinolin-1(2H)-ylidene)propan-2-one (**19a**)

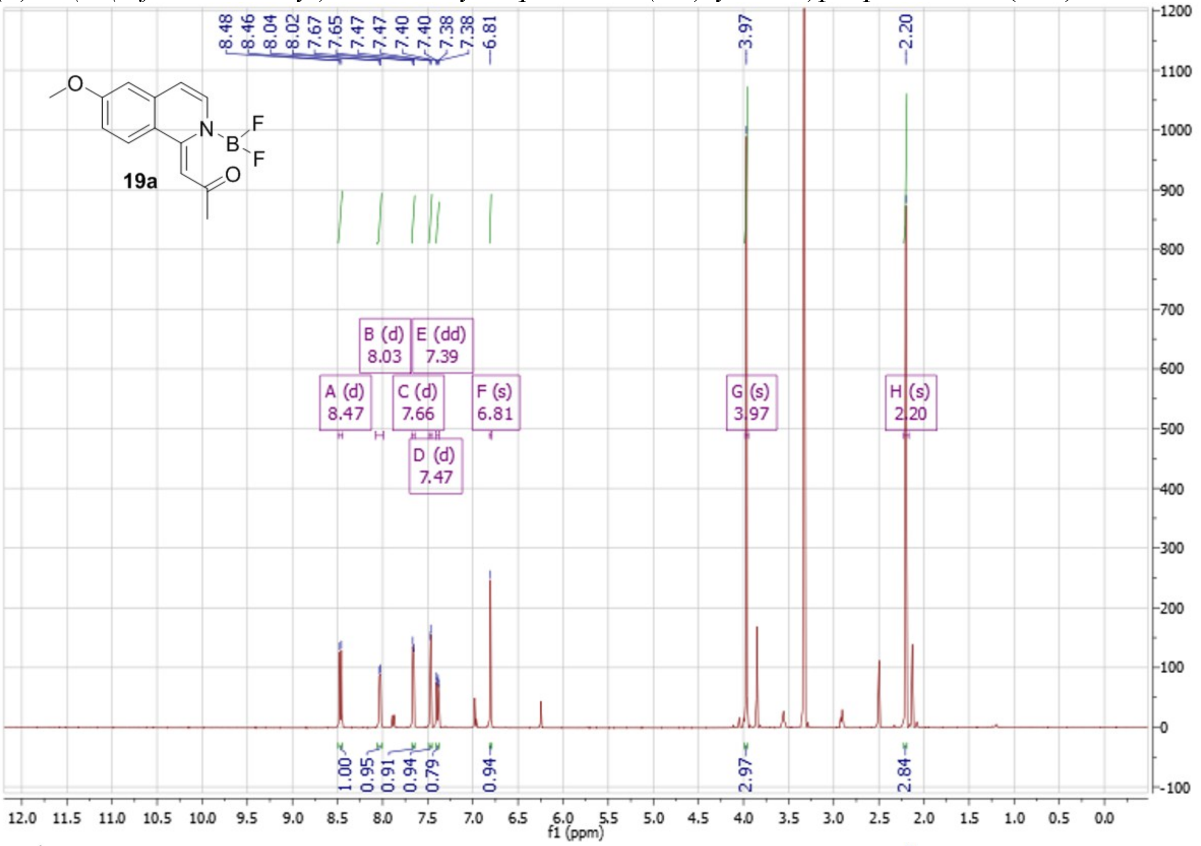

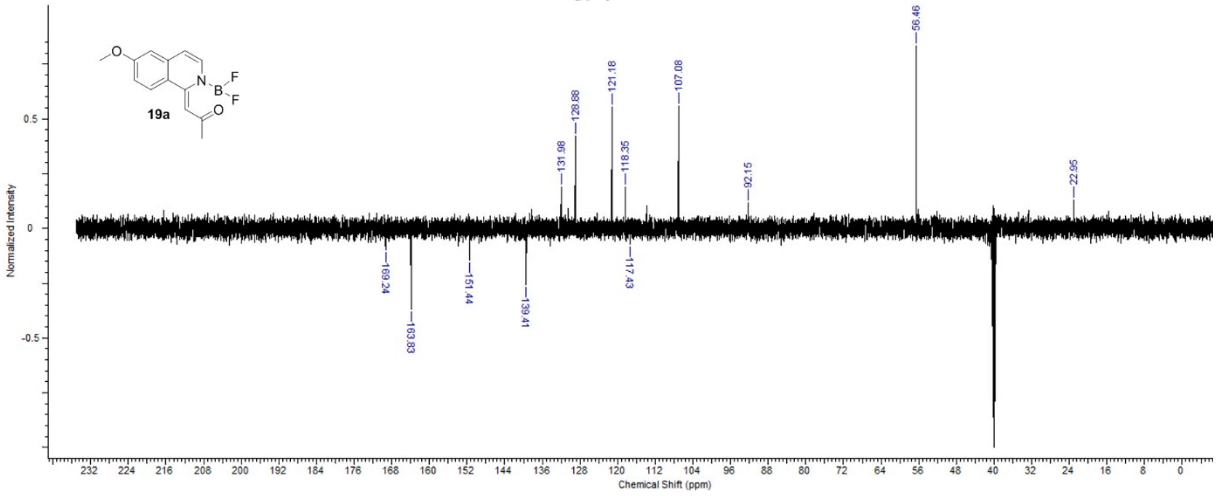

(Z)-1-(2-(difluoroboranyl)-6,7-dimethoxyisoquinolin-1(2H)-ylidene)propan-2-one (**19b**)

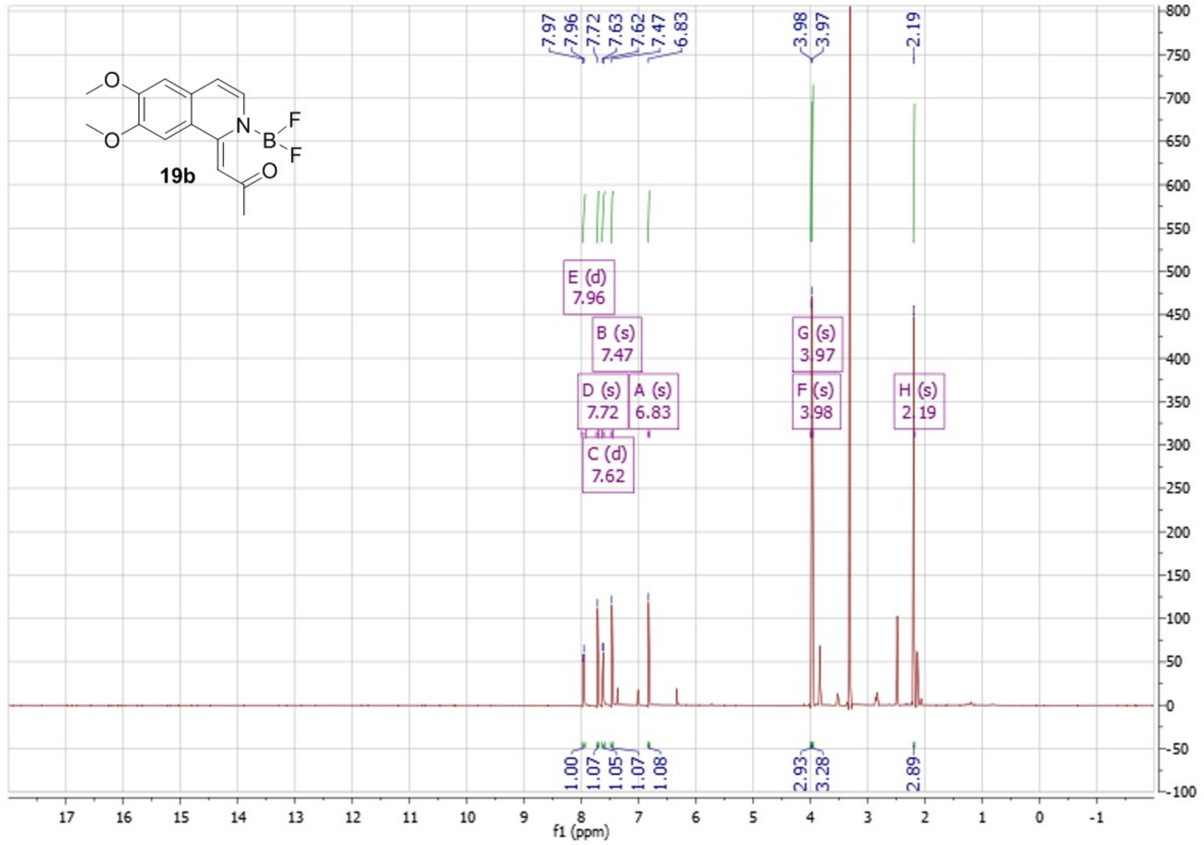

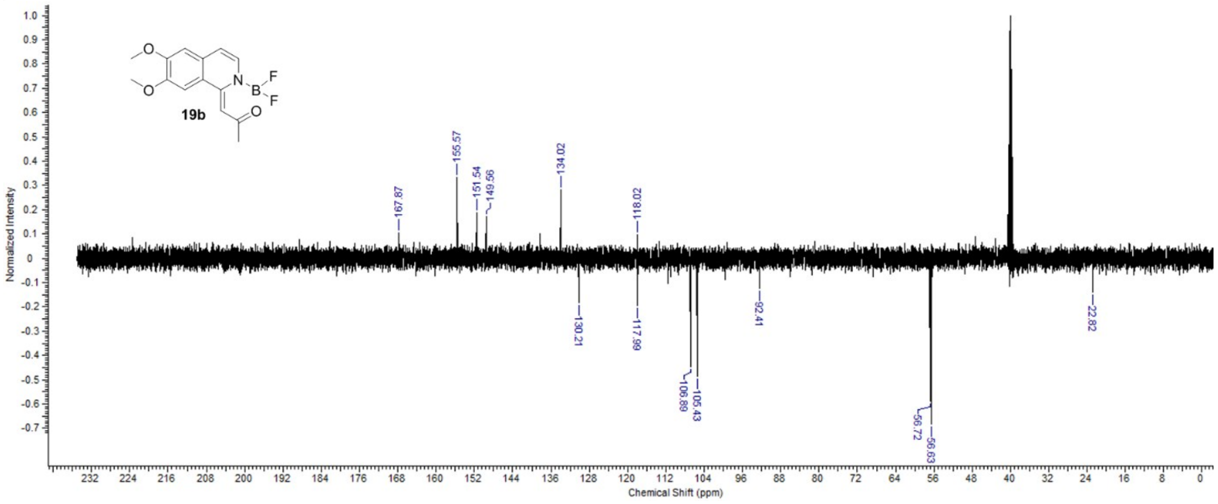

(Z)-2-(2-(difluoroboranyl)-6-methoxyisoquinolin-1(2H)-ylidene)-1-phenylethan-1-one (**19c**)

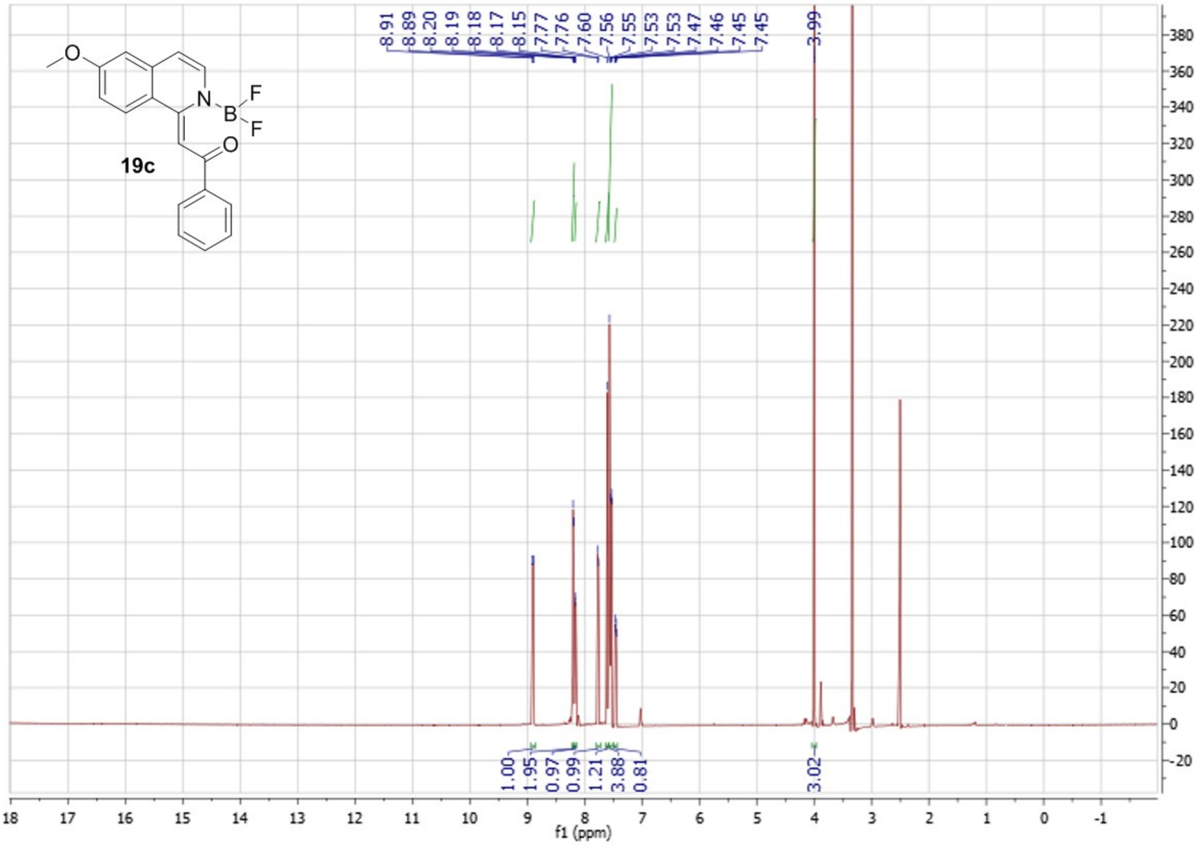

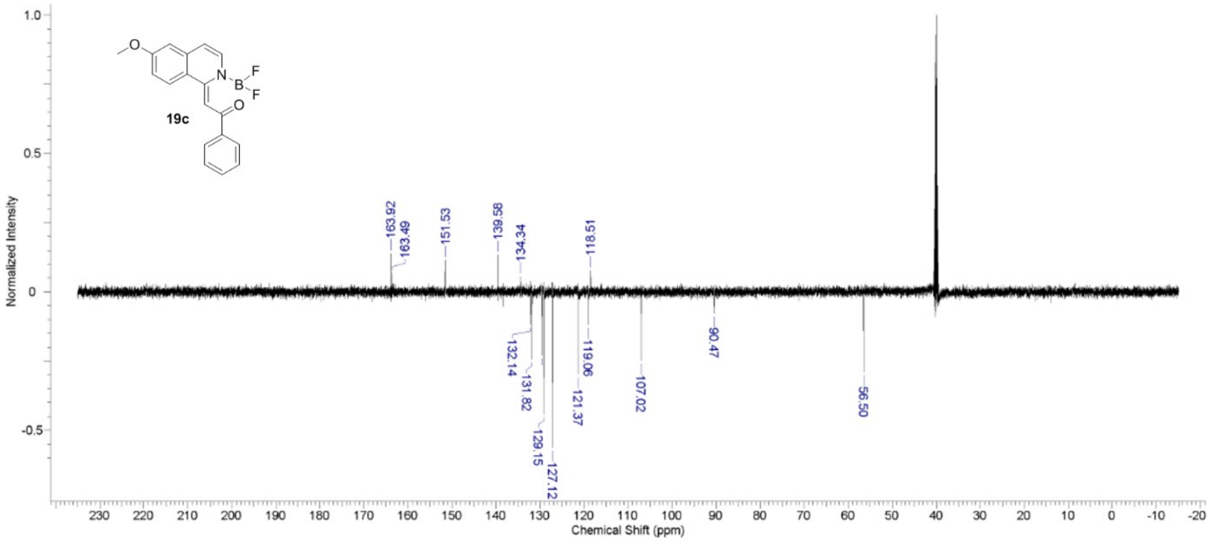

(Z)-2-(2-(difluoroboranyl)-6,7-dimethoxyisoquinolin-1(2H)-ylidene)-1-phenylethan-1-one **(19d)**

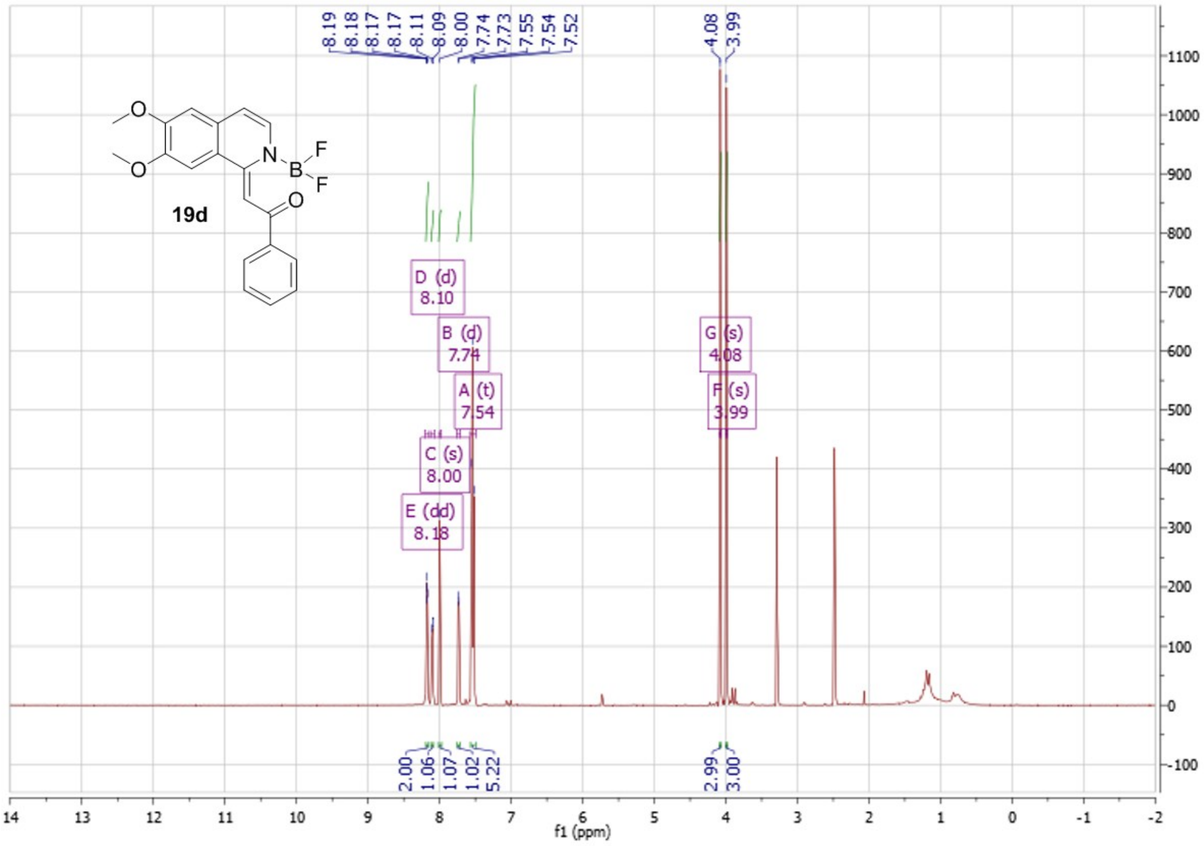

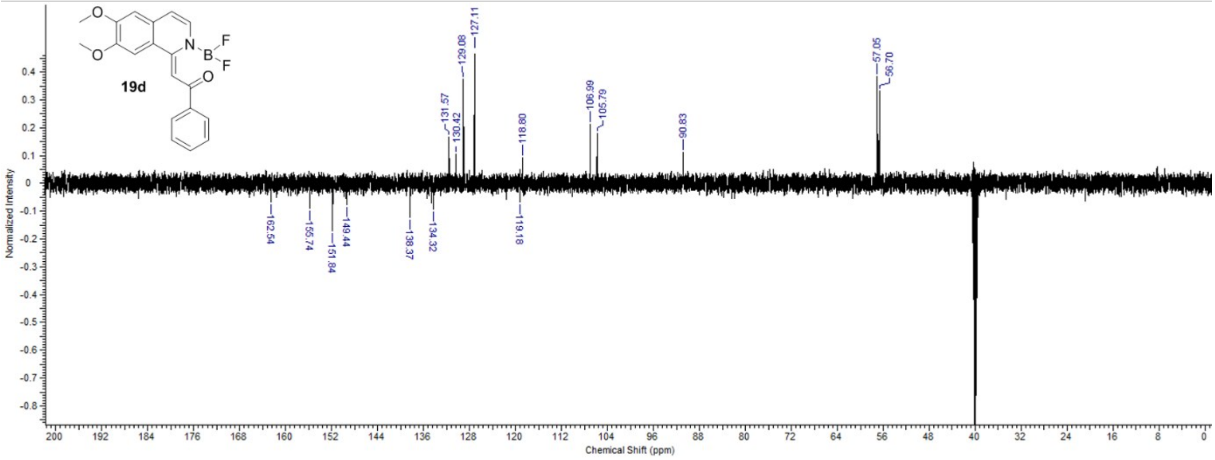

(Z)-2-(2-(difluoroboranyl)-6-methoxyisoquinolin-1(2H)-ylidene)-1-(thiophen-2-yl)ethan-1-one (**19e**)

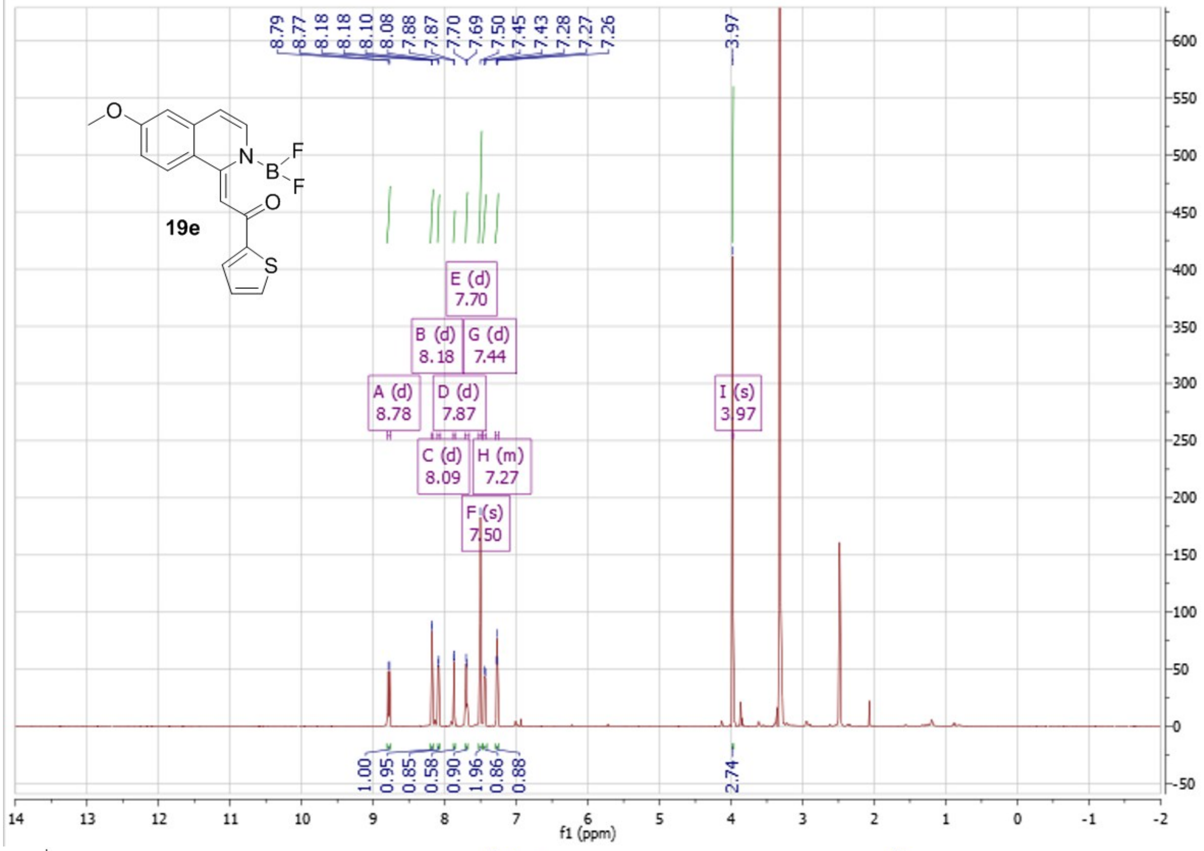

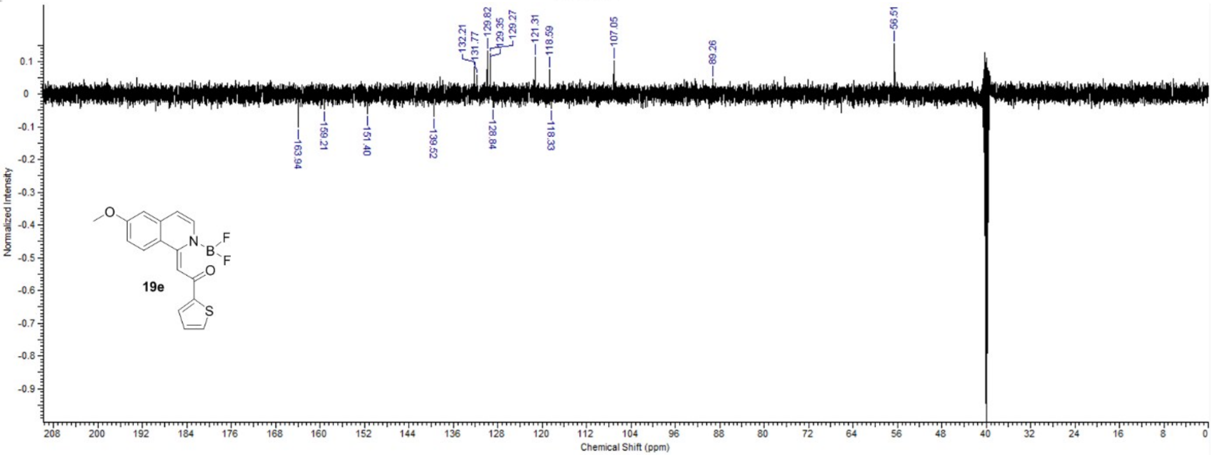

(Z)-2-(2-(difluoroboranyl)-6,7-dimethoxyisoquinolin-1(2H)-ylidene)-1-(thiophen-2-yl)ethan-1-one (**19f**)

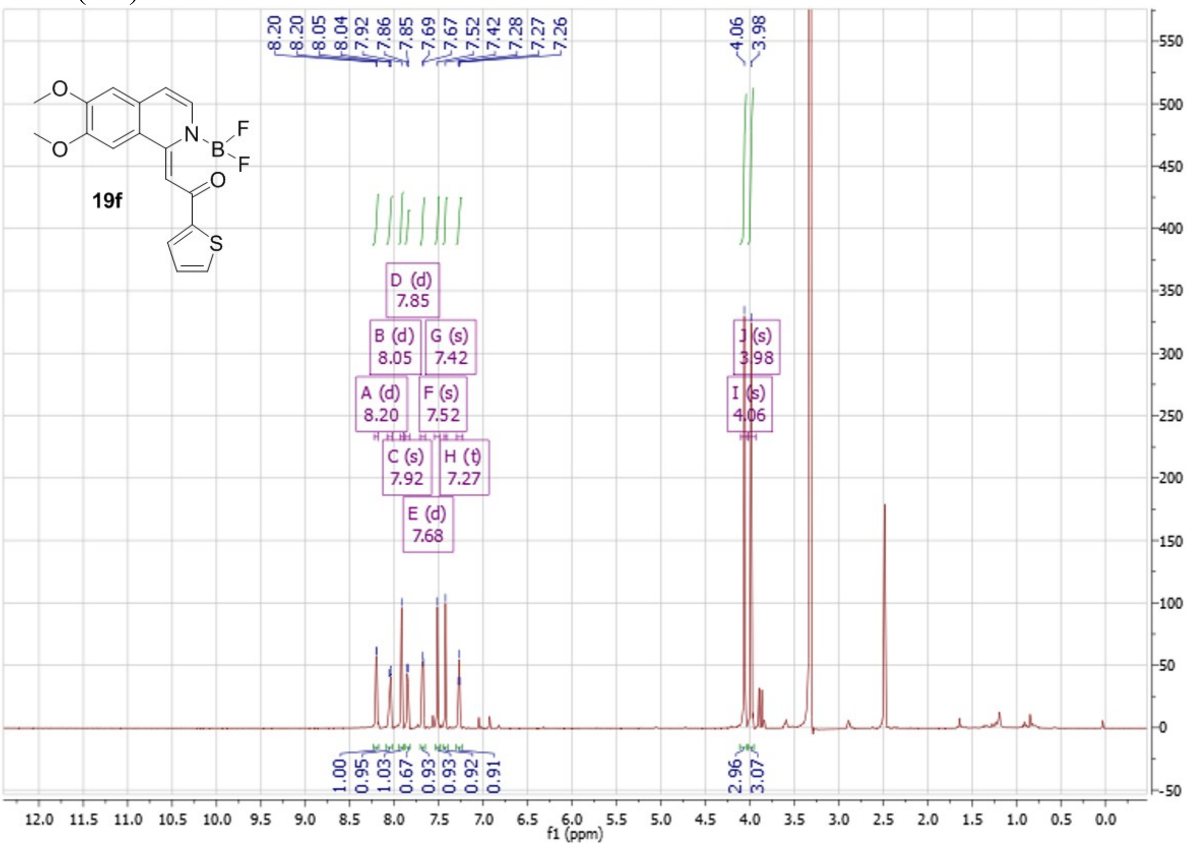

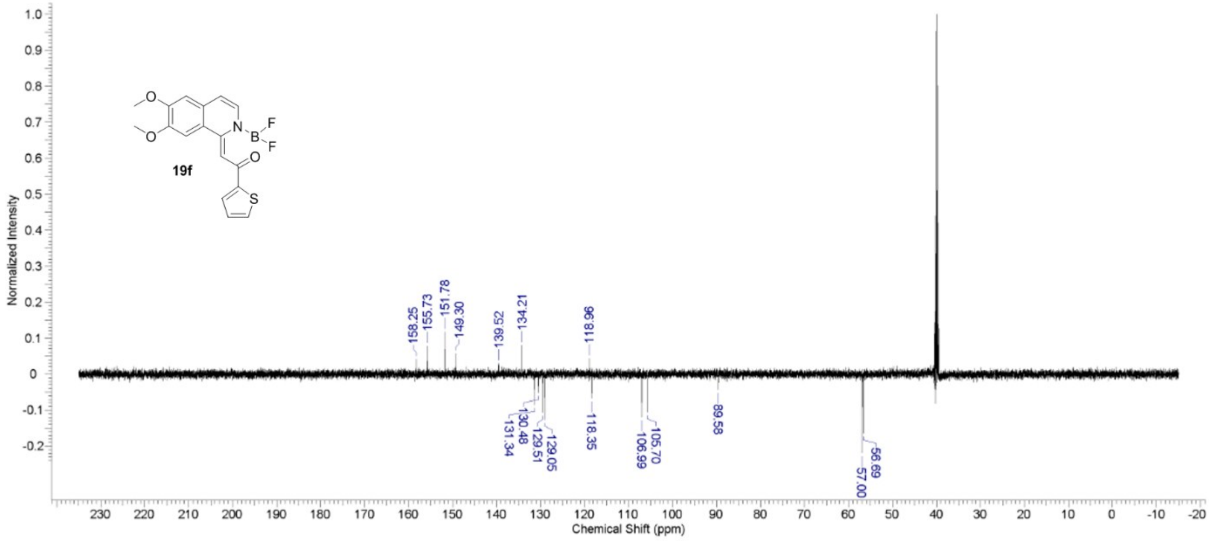

(Z)-1-(2-(diphenylboranyl)-6,7-dimethoxy-3,4-dihydroisoquinolin-1(2H)-ylidene)propan-2-one (**20b**)

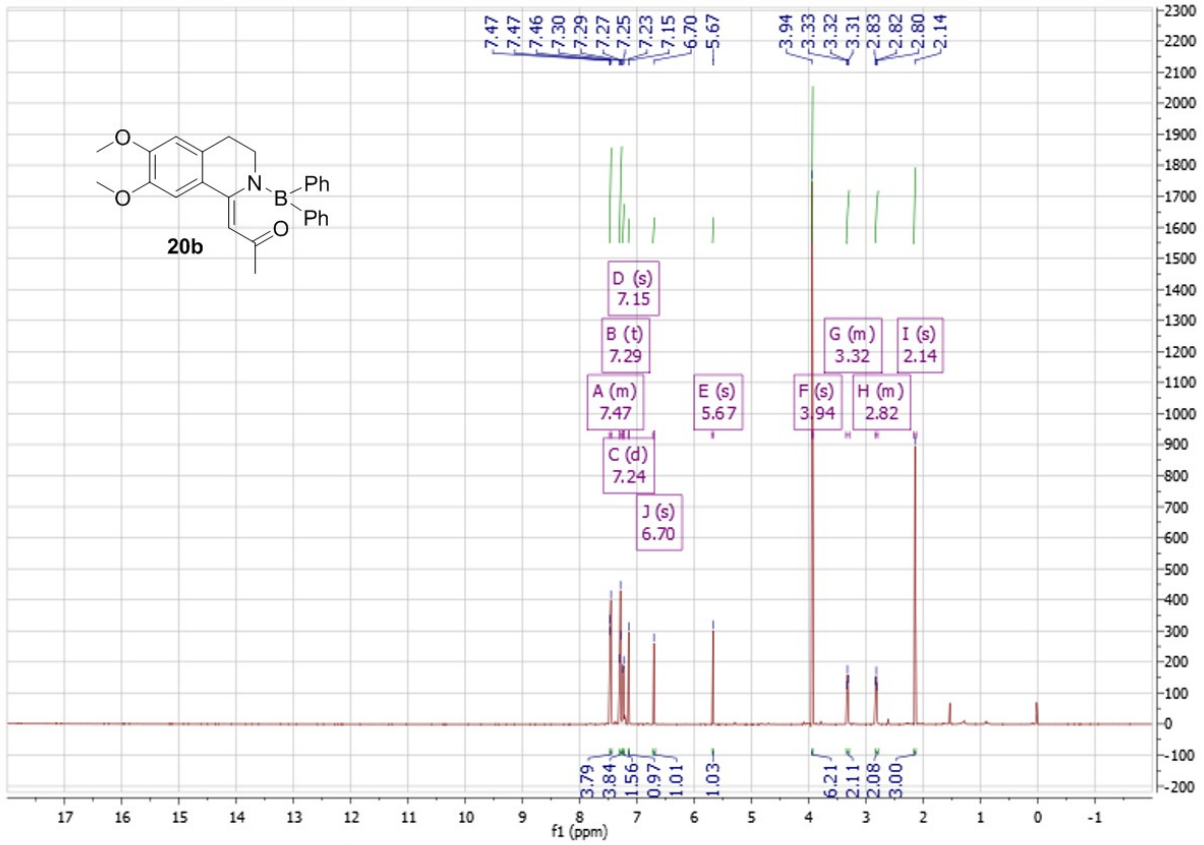

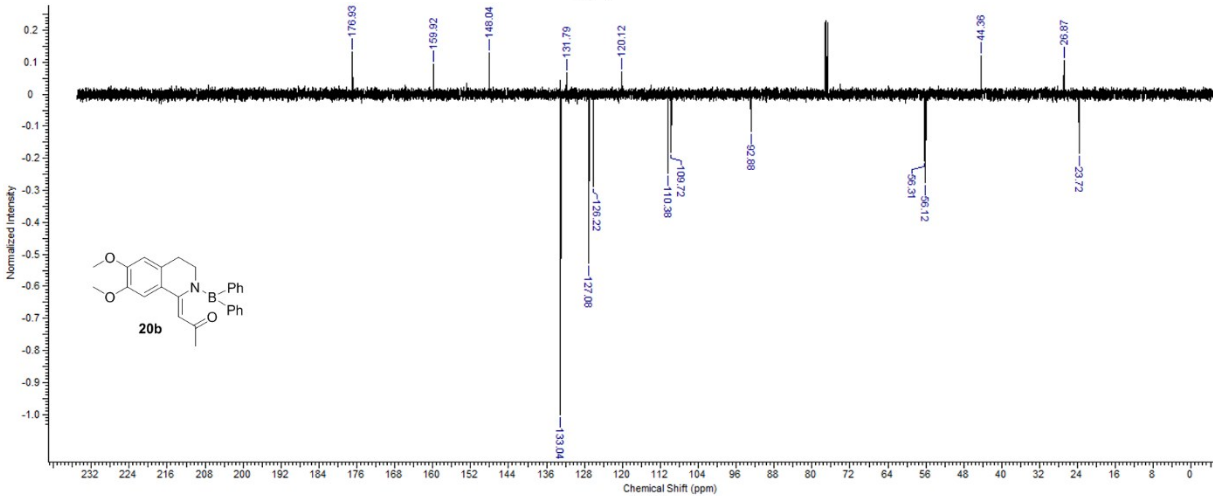

(Z)-1-(6,7-dimethoxy-3,4-dihydroisoquinolin-1(2H)-ylidene)propan-2-one tris(perfluorophenyl)borane salt (**22b**)

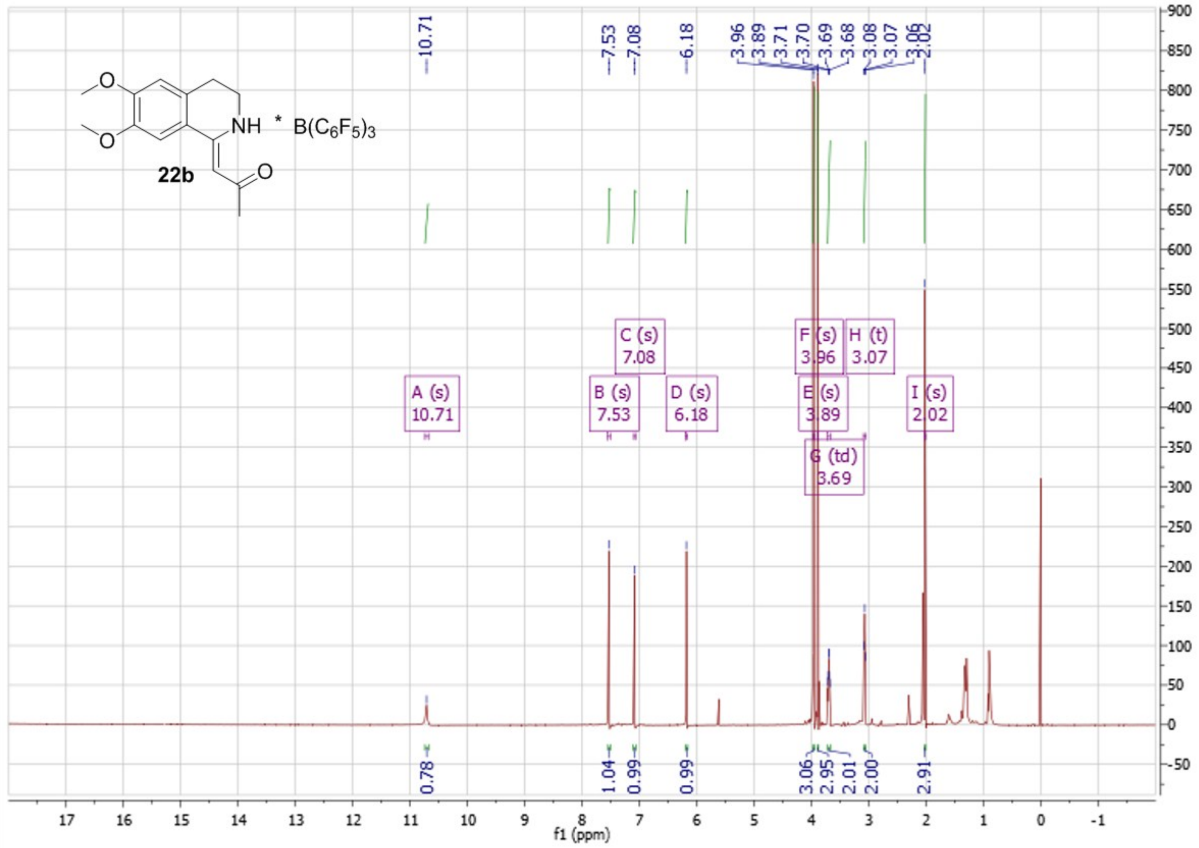

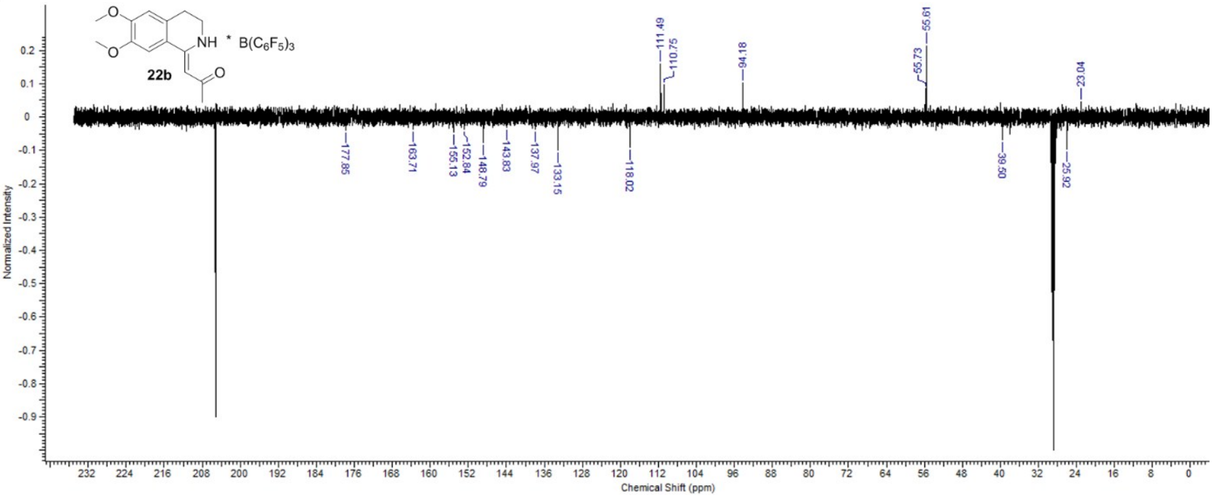

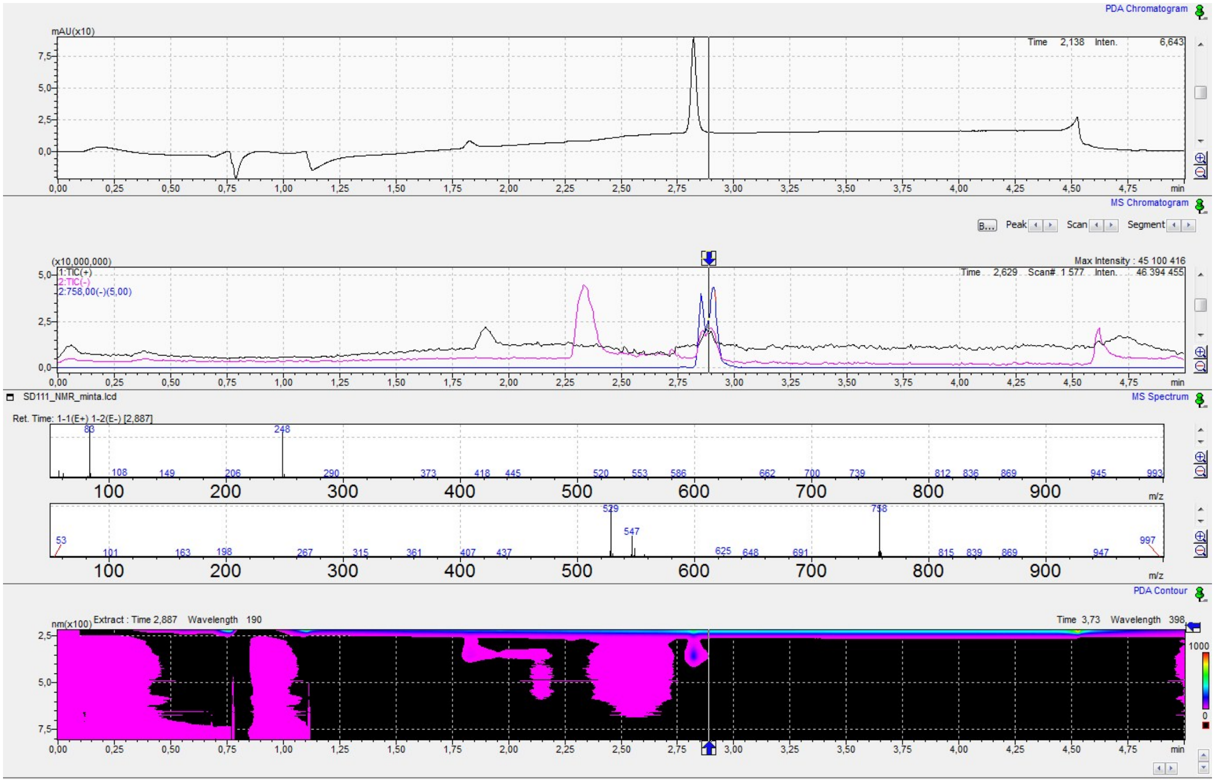

(Z)-1-(2-(bis(perfluorophenyl)boranyl)-6,7-dimethoxy-3,4-dihydroisoquinolin-1(2H)-ylidene)propan-2-one (**21b**)

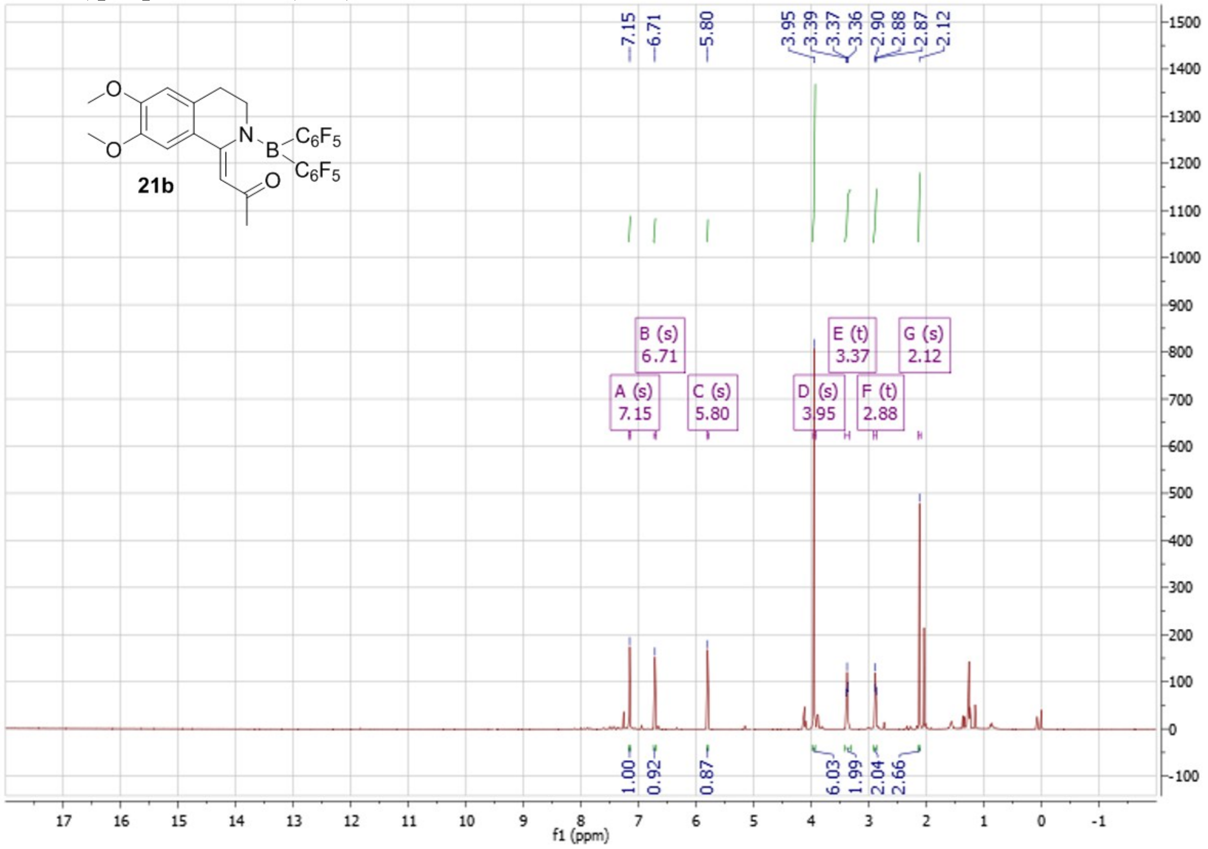

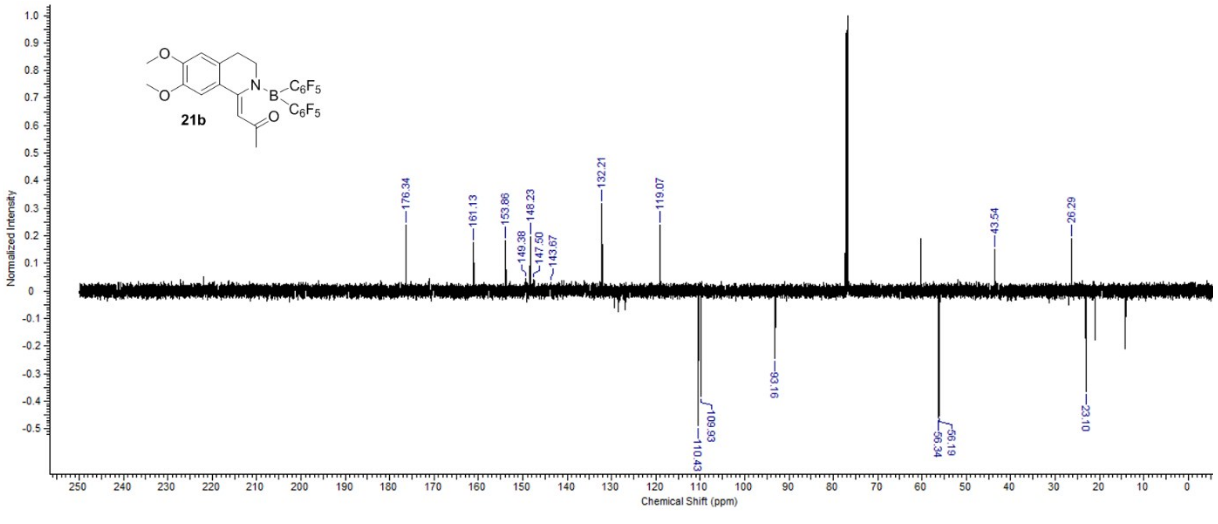

(Z)-2-(2-(bis(perfluorophenyl)boranyl)-6-(pyrrolidin-1-yl)-3,4-dihydroisoquinolin-1(2H)-ylidene)-1-phenylethan-1-one (**21l**)

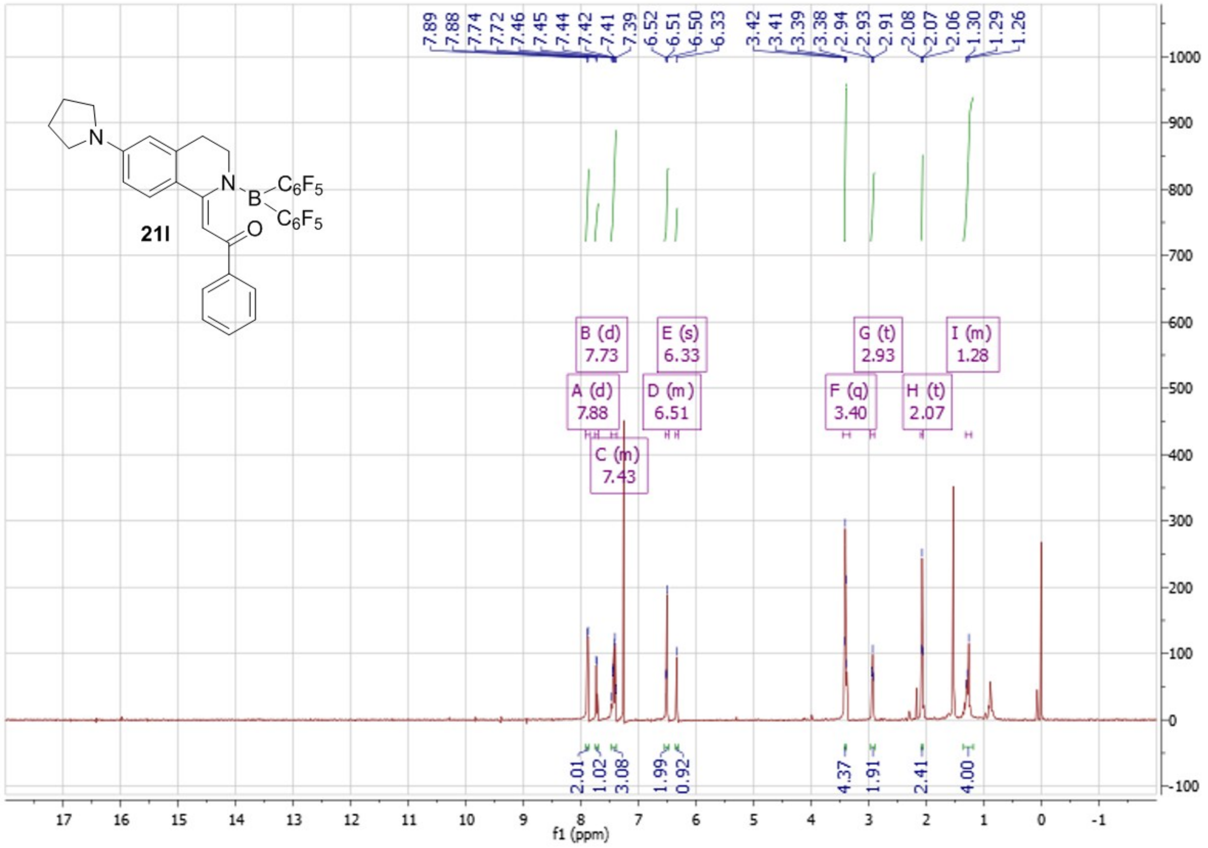

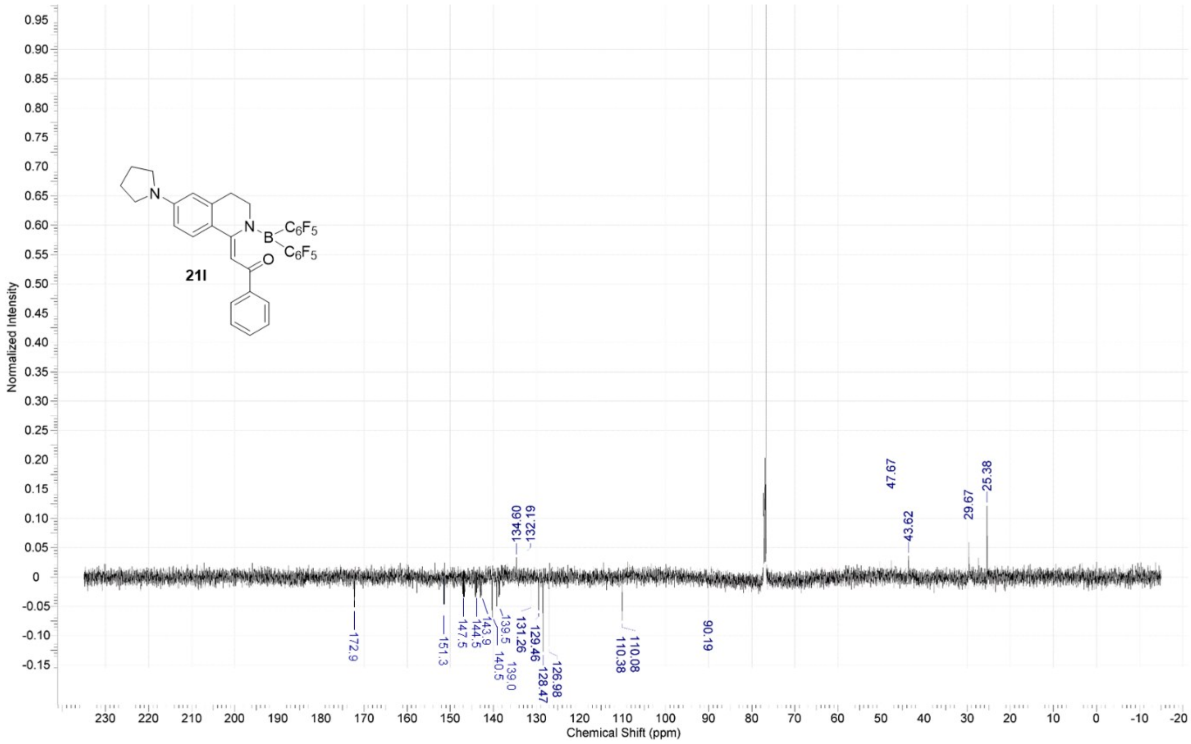

(Z)-N-acetyl-S-(4-(2-(2-(difluoroboranyl)-6,7-dimethoxyisoquinolin-1(2H)-ylidene)acetyl)-2,3,5,6-tetrafluorophenyl)cysteine (**24**)

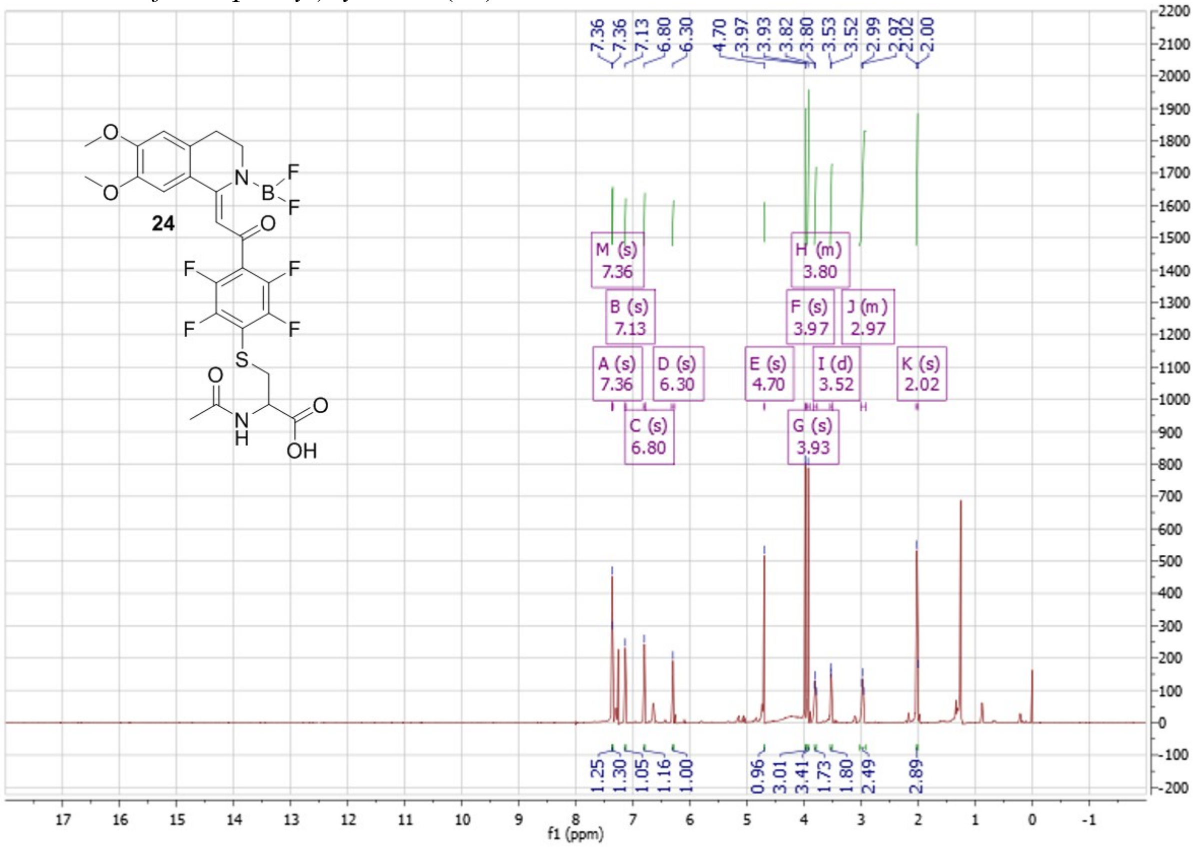

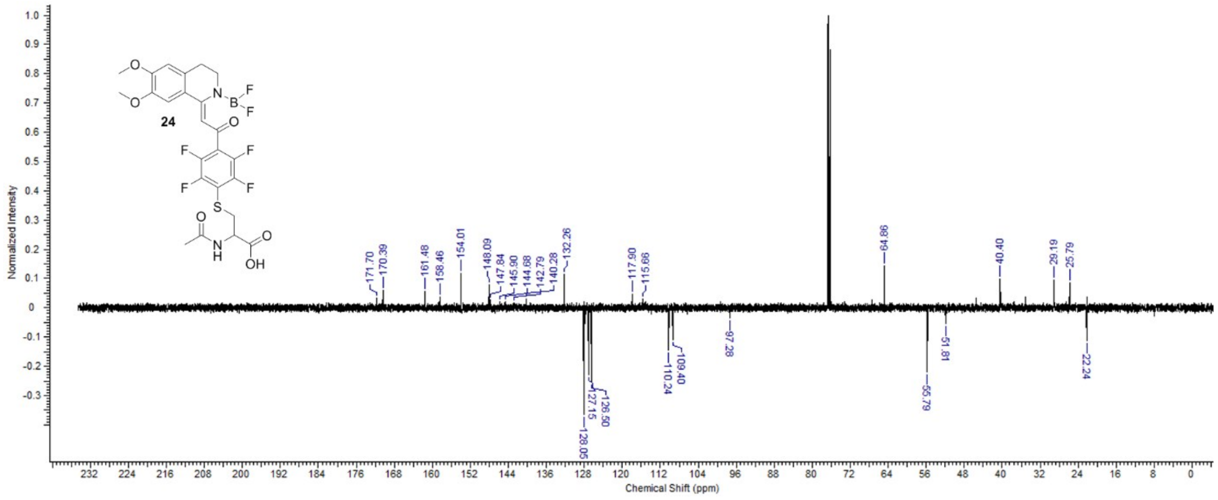

4. References

1 G. Grabner, K. Rechthaler, B. Mayer, G. Köhler and K. Rotkiewicz, *J. Phys. Chem. A*, 2000, **104**, 1365–1376.

2 M. Johannes and K.-H. Altmann, *Org. Lett.*, 2012, **14**, 3752–3755.

3 M. Langlois, B. Bremont, S. Shen, A. Poncet, J. Andrieux, S. Sicsic, I. Serraz, M. Mathe-Allainmat, P. Renard and P. Delagrange, *J. Med. Chem.*, 1995, **38**, 2050–2060.

4 S. Akhter, B. A. Lund, A. Ismael, M. Langer, J. Isaksson, T. Christopeit, H.-K. S. Leiros and A. Bayer, *Eur. J. Med. Chem.*, 2018, **145**, 634–648.

5 J. Václavík, J. Pecháček, B. Vilhanová, P. Šot, J. Januščák, V. Matoušek, J. Přech, S. Bártová, M. Kuzma and P. Kačer, *Catal. Letters*, 2013, **143**, 555–562.

6 R. Soni, F. K. Cheung, G. C. Clarkson, J. E. D. Martins, M. A. Graham and M. Wills,

Org. Biomol. Chem., 2011, **9**, 3290.
7 X. Ning, W. Ruyong and W. Liang, WO2014089324 A1.
8 M. Henrich, T. Weil, S. Müller, V. Kauss and E. Erdmare, WO 2009/095253 A1.
9 F. Haraszti, T. Igricz, G. Keglevich, M. Milen and P. Abranyi-Balogh, *Curr. Org. Chem.*, 2018, **22**, 912–922.
10 O. Barun, P. K. Mohanta, H. Ila and H. Junjappa, *Synlett*, 2000, **2000**, 0653–0657.
